# A Remarkable New Family of Jurassic Insects (Neuroptera) with Primitive Wing Venation and Its Phylogenetic Position in Neuropterida

**DOI:** 10.1371/journal.pone.0044762

**Published:** 2012-09-18

**Authors:** Qiang Yang, Vladimir N. Makarkin, Shaun L. Winterton, Alexander V. Khramov, Dong Ren

**Affiliations:** 1 College of Life Sciences, Capital Normal University, Beijing, China; 2 Institute of Biology and Soil Sciences, Far Eastern Branch of the Russian Academy of Sciences, Vladivostok, Russia; 3 California State Collection of Arthropods, California Department of Food and Agriculture, Sacramento, California, United States of America; 4 Borissiak Paleontological Institute of the Russian Academy of Sciences, Moscow, Russia; University of California, Berkeley, United States of America

## Abstract

**Background:**

Lacewings (insect order Neuroptera), known in the fossil record since the Early Permian, were most diverse in the Mesozoic. A dramatic variety of forms ranged in that time from large butterfly-like Kalligrammatidae to minute two-winged Dipteromantispidae.

**Principal Findings:**

We describe the intriguing new neuropteran family Parakseneuridae fam. nov. with three new genera and 15 new species from the Middle Jurassic of Daohugou (Inner Mongolia, China) and the Early/Middle Jurassic of Sai-Sagul (Kyrgyzstan): *Parakseneura undula* gen. et sp. nov., *P. albomacula* gen. et sp. nov., *P. curvivenis* gen. et sp. nov., *P. nigromacula* gen. et sp. nov., *P. nigrolinea* gen. et sp. nov., *P. albadelta* gen. et sp. nov., *P. cavomaculata* gen. et sp. nov., *P. inflata* gen. et sp. nov., *P. metallica* gen. et sp. nov., *P. emarginata* gen. et sp. nov., *P. directa* gen. et sp. nov., *Pseudorapisma jurassicum* gen. et sp. nov., *P. angustipenne* gen. et sp. nov., *P. maculatum* gen. et sp. nov. (Daohugou); *Shuraboneura ovata* gen. et sp. nov. (Sai-Sagul). The family comprises large neuropterans with most primitive wing venation in the order indicated by the presence of ScA and AA1+2, and the dichotomous branching of MP, CuA, CuP, AA3+4, AP1+2. The phylogenetic position of Parakseneuridae was investigated using a phylogenetic analysis of morphological scoring for 33 families of extinct and extant Neuropterida combined with DNA sequence data for representatives of all extant families. Parakseneuridae were recovered in a clade with Osmylopsychopidae, Prohemerobiidae, and Ithonidae.

**Conclusions/Significance:**

The presence of the presumed AA1+2 in wings of Parakseneuridae is a unique plesiomorphic condition hitherto unknown in Neuropterida, the clade comprising Neuroptera, Megaloptera, Raphidioptera. The relative uncertainty of phylogenetic position of Parakseneuridae and the majority of other families of Neuroptera reflects deficient paleontological data, especially from critical important periods for the order, earliest Triassic and latest Triassic/earliest Jurassic.

## Introduction

The insect order Neuroptera (lacewings) comprises today about 5500 species of 15 families [1]. This revised number of families follows the recent synonymy of Polystoechotidae with Ithonidae [2] and inclusion of Rhachiberothidae as a subfamily of Berothidae [3]. Lacewings were most diverse in the Mesozoic, particularly in the Late Jurassic and Early Cretaceous, with the vast majority of extant and extinct families recorded from deposits of this antiquity [4], [5]. Morphological diversity of Neuroptera in the Jurassic/early Cretaceous was dramatic, with large butterfly-like Kalligrammatidae together with minute two-winged mantispid-like Dipteromantispidae whose hind wings were modified into small haltere-like structures [6], [7]. These and many other unusual groups of Neuroptera are now only known from the fossil record.

In past decade, five remarkable new families were discovered from the Mesozoic of China, i.e., Grammolingiidae, Aetheogrammatidae, Ascalochrysidae, Saucrosmylidae and Dipteromantispidae [7]–[12], and at least one awaits description (see [6]). In the present paper we describe another intriguing new family, Parakseneuridae, comprising large neuropterans with generalized wing venation.

In an attempt to understand the relationship of Parakseneuridae fam. nov. to other Neuroptera we undertook a phylogenetic analysis of morphological scoring for 33 families of extinct and extant Neuropterida combined with DNA sequence data for representatives of all extant families. Earlier attempts at reconstructing Neuroptera phylogeny have ranged from subjective narratives (e.g., [13]–[16]) to more extensive quantitative analyses of morphological data (e.g., [17]–[21]), molecular DNA sequences (e.g., [22], [23]), or a combination of both (e.g., [24]).

Incorporation of morphological data from compression fossil taxa into phylogenetic analyses can be problematic, as the majority of these taxa are represented only by wings. To resolve this problem, it is possible to use a currently accepted hypothesis of phylogeny of extant Neuroptera and incorporate fossil taxa in accordance with their probable relationships to these taxa. Such incorporation of fossil taxa into current phylogeny was done by Grimaldi and Engel [4] based on the phylogeny of extant taxa of Aspöck and colleagues [19], and again by Jepson and Penney [5] who used additional palaeontological data. While not quantitative, this method of amending a previous quantitative result with additional narrative is a useful summary. Winterton and Makarkin [2] took this a step further by conducting a phylogenetic analysis of both fossil and living taxa of Ithonidae, and including DNA sequences for a significant number of living taxa, recovering a robust phylogeny for the entire group. Herein, we present the results of a comparative analysis of characters of all 30 families of Neuroptera, including Parakseneuridae fam. nov., two families of Megaloptera, and one family of Raphidioptera based on the combined morphological and DNA sequence data. Parakseneuridae fam. nov. is described and figured with three new genera and 15 new species from the Jurassic-aged deposits of China and Kyrgyzstan.

## Materials and Methods

### Material

We examined 25 specimens from the locality of Daohugou, Inner Mongolia, China, and nine specimens from the locality of Sai-Sagul, Kyrgyzstan (of the latter, only two are described, the others are fragmentary or poorly preserved). The specimens are housed in the fossil insect collection of the Key Laboratory of Insect Evolution & Environmental Changes, College of Life Sciences, Capital Normal University, Beijing, China, and in Paleontological Institute of the Russian Academy of Sciences, Moscow, Russia, respectively. No specific permits were required for the described field studies.

The specimens were examined under a Leica MZ12.5 dissecting microscope (Leica, Wetzlar, Germany). Line drawings were prepared with CorelDraw 12 graphics software with the aid of Photoshop CS2 (Adobe Systems, Mountain View, CA). All wings are figured with the apex to the right. The photographs were taken by Nikon D100 Digital Camera. Magnified images of parts of the specimens were taken with a Nikon SMZ1000.

### Terminology and Abbreviations

Wing venation terminology broadly follows Kukalová-Peck and Lawrence [25] except treating the median vein. The principal hypotheses on which this terminology is based are: (1) each main vein has anterior and posterior branch (sector) which have no common stem; (2) anterior branch (sector) is convex and posterior is concave [26]. Béthoux [27] who in general accepts this hypothesis throws doubt upon the former point by stating that “the hypothesis of primitively distinct origins of main vein sectors (i.e., main veins not stemmed) needs further demonstrative evidence” (p. 55). Indeed, the median vein in most insect orders has a basal stem (all Palaeoptera and plecopteroid and orthopteroid orders among Neoptera). This condition is considered as apomorphic, as well as a condition found in the blattoid (Blattoneoptera), hemipteroid (Hemineoptera, Paraneoptera) and Endopterygota (Endoneoptera) orders in which MA is hypothesized as completely fused basally with the radius, diverging apically from RP [25], [28]. A plesiomorphic (primitive) condition of M (i.e., not stemmed, forked immediately at the base into MA and MP which then continue as distinct veins) is not found in any insect taxon (including the oldest known). Therefore, the hypothesis of basal fusion of MA and R at least in Neuropterida should be considered as unproven. In this paper, we consider M of Neuropterida to primitively possess a basal stem. In particular, this indirectly implies from principal similarity of the venation of oldest Neoptera and the Permian Neuroptera (cf. e.g., Figure 1A in [29]; Figure 3 in [30]). The anterior branch of the apparent M (which is here named MA) is often concave in Neuroptera. According the hypothesis of Kukalová-Peck [26] MA should be convex. However, the convex AP in the forewing and the concave CuA in the hind wing found in most (if not all) Neuroptera also contradicts the hypothesis of Kukalová-Peck [26]: AP should be concave, and CuA convex. Therefore, the concave MA of Neuroptera is not exceptional.

There are three anal veins in the majority of neuropteran taxa: AA3+4 ( = 1A of Comstock [31]), AP1+2 ( = 2A), and AP3+4 ( = 3A). Nel and colleagues [32] named these three anal veins as AA ( = 1A), AP1 ( = 2A), and AP2 ( = 3A). Kukalová-Peck and Lawrence [25] believe that AA1+2 is lost in all Neuropterida (see Figures 5, 6 in [25]). In Parakseneuridae unlike other Neuropterida, the presumed AA1+2 is present (see below). The problem is that all anal veins are convex. Therefore, the terminology of Kukalová-Peck and Lawrence [25] concerning anal veins should be considered as only preliminary.

Terminology of wing spaces and details of the venation (e.g., subcostal veinlets) follows Oswald [33].

Venation abbreviations used in the text and Figures: AA, Analis Anterior; AP, Analis Posterior; cf, claval flexion fold (line); Cu, Cubitus; CuA, Cubitus Anterior; CuP, Cubitus Posterior; hp, humeral plate; hv, humeral veinlet; M, Media; MA, Media Anterior; mf, median flexion fold (line); MP, Media Posterior; R, Radius; RA, Radius Anterior; RP, Radius Posterior; RP1, proximal-most branch of Radius Posterior; RP2, branch of Radius Posterior distal to RP1; sb, sclerotized bulge; ScA, Subcosta Anterior; ScP, Subcosta Posterior; tg, tegula; vs, unknown veinal structure.

### Phylogenetic Analyses

#### Taxa

Thirty families of Neuroptera, one family of Raphidioptera and two families of Megaloptera were selected for the analyses (Table S3). The status and composition of several fossil families (i.e., Solenoptilidae, Epigambriidae, Glottidiidae, “Cratochrysidae” and Osmylitidae) are not clear yet and these were not included in the analysis as well as at least one undescribed Mesozoic family and the strongly specialized Cretaceous Dipteromantispidae [7]. The family Nymphitidae is invalid as its type genus belongs to Nymphidae (Makarkin & Archibald, ongoing research).

The extensive work on the taxonomy of the majority of fossil families of Neuroptera preceded this paper [34] (and subsequent papers of VM, QY, DR and unpublished materials of VM). This allowed us to use for the analysis only valid and presumably monophyletic families. Archeosmylidae is treated as in [35], Panfiloviidae as in [36], Palaeoleontidae as in [37], Brongniartiellidae as in [38]. Berothidae includes Mesithonidae and Rhachiberothidae [3], [6]. Ithonidae includes Polystoechotidae and Rapismatidae [2]. Mesochrysopidae includes Allopteridae and Tachinymphidae [39]. Myrmeleontidae includes Araripeneuridae but its genera are not used in this analysis. Mesoberothidae probably represents oldest known Berothidae [40], but its genera are not included in the analysis. In Prohemerobiidae, only the heterogeneous genus *Prohemerobius* Handlirsch was used for the analysis. Only extant genera of Psychopsidae and several fossil genera most similar to these (as characterized by Jepson and colleagues [41]) were used for the analysis. In Osmylopsychopidae, only type genus and undescribed taxa from the Triassic Madygen Formation (Kyrgyzstan) were used for the analysis. For all families, unpublished materials of VM (photographs of types and undescribed taxa) were used.

#### Morphological Characters

Forty-four morphological characters were scored for the morphological dataset. The majority of these are characters of the venation, which is often complicated in Neuroptera and highly variable within a family. For this reason, only character states of a presumed family ground-plan were used for the analysis (especially when characters are polymorphic). Each polymorphic (variable) character of the venation was examined within each family to define the putative plesiomorphic (‘primitive’) condition as a subjective determination. As expected, in some cases this was difficult to define due to incomplete fossils or two (or more) conditions equally representative as plesiomorphic in a given family. For example, the arrangement of crossveins in the radial space (Character 27) is highly variable within the order and in both extremes are found in many families. We identified four states: (0) all crossveins are sporadically distributed; (1) most crossveins are sporadically distributed but some form gradate series; (2) most crossveins are arranged in gradate series, but there are some sporadically distributed crossveins; and (3) all crossveins are arranged in one or several gradate series. The state (0) is likely plesiomorphic for the order, and transformation series might be: (0)→(1)→(2)→(3). In the Mesozoic family Mesochrysopidae, the state (0) is not found, but three other are present. Of these, state (1) is found in the genera distributed in the Cretaceous; state (2) is found in the Jurassic genus *Mesochrysopa* Handlirsch and state (3) in three other Jurassic genera. The Cretaceous genera are clearly derived based on other characters, therefore, we used for the analysis state (2) and not (1) presuming that *Mesochrysopa* has the most generalized venation within family and that state (1) represents a reversal in the Cretaceous genera. However, state (3) also may to be the most ‘plesiomorphic’ condition in this family as it is found in the oldest (Early Jurassic) genus. Descriptions of character states of morphological characters are given in Table S1.

#### Phylogenetic Analyses

DNA sequences for two ribosomal genes (16S and 18S rDNA) and two protein-encoding genes (cytochrome oxidase I (COI) and the CPSase region of carbamoyl-phosphate synthetase-aspartate transcarbamoylase-dihydroorotase (CAD)) were retrieved from Genbank (Table S2). Sequences were aligned following [24] using a combination of automated and manual alignment methods based on the particular locus. Alignment ambiguous sections and introns in protein encoding gene were deleted prior to analysis. Sequence data for all extant families were then combined with morphological scoring for all valid extant and extinct families (see Table S3) and parsimony analyses conducted using PAUP*4.0b10 [42] using a heuristic tree search protocol with 30 replicate random addition sequences and tree bisection and reconnection (TBR). Sequence lengths, average base frequencies and sequence divergences reflect those obtained in [24]. All characters were equally weighted and unordered, with 657 being parsimony-informative.

### Institutional Abbreviation

**CNUB**, Capital Normal University, Beijing, China; **PIN**, Paleontological Institute of the Russian Academy of Sciences, Moscow, Russia.

### Nomenclatural Acts

The electronic version of this document does not represent a published work according to the International Code of Zoological Nomenclature (ICZN), and hence the nomenclatural acts contained in the electronic version are not available under that Code from the electronic edition. Therefore, a separate edition of this document was produced by a method that assures numerous identical and durable copies, and those copies were simultaneously obtainable (from the publication date noted on the first page of this article) for the purpose of providing a public and permanent scientific record, in accordance with Article 8.1 of the Code. The separate print-only edition is available on request from PLOS by sending a request to PLOS ONE, Public Library of Science, 1160 Battery Street, Suite 100, San Francisco, CA 94111, USA along with a check for $10 (to cover printing and postage) payable to “Public Library of Science”.

The online version of the article is archived and available from the following digital repositories: PubMedCentral (www.pubmedcentral.nih.gov/) and LOCKSS (http://www.lockss.org/lockss/). In addition, this published work and the nomenclatural acts it contains have been registered in ZooBank, the proposed online registration system for the ICZN. The ZooBank LSIDs (Life Science Identifiers) can be resolved and the associated information viewed through any standard web browser by appending the LSID to the prefix “http://zoobank.org/”. The LSID for this publication is: urn:lsid:zoobank.org:pub: 5E2032B8-F478-4D37-A22F-A2FC24F23BBE.

## Localities

### Daohugou

Daohugou Village is located in Shantou Township, Ningcheng County, Inner Mongolia, China (see map in [43]). The fossil-bearing beds consist of intercalated, fine-grained lacustrine deposits and fine volcanic ash that unconformably overlay pre-Cambrian rocks [44]. There is no agreement about the fossil-bearing stratigraphic sequence at Daohugou due to strong tectonic activities and the subsequent folding of sediments [45]. The majority of authors believe that the volcanic rocks of the Tiaojishan Formation overlay the fossil-bearing beds [43], [46]–[49]. However, some authors consider this sequence to be overturned and believe that the volcanic rocks underlay the fossil-bearing beds [45], [50], [51]. Accordingly, the age of these fossil-bearing beds has been considered differently: Middle Jurassic (Bathonian) [49]; late Middle Jurassic to early Late Jurassic (Callovian to Oxfordian) [52], [53]; Late Jurassic (Oxfordian to Kimmeridgian) [54]; Late Jurassic or younger [50].

The age of the volcanic rocks overlying the fossil-bearing beds in the Daohugou area ranges from 159.8±0.8 to 165±2.5 Ma based on ^40^Ar/^39^Ar and SHRIMP ^206^Pb/^238^U dating [43], [46], [50], [51], [55]. The age of one layer of tuffs within the fossil-bearing beds is 165±1.2 Ma by SHRIMP ^206^Pb/^238^U dating [55]. Based on these data, we estimate the age of the fossil-bearing beds at Daohugou to be Bathonian to Callovian (Middle to late Middle Jurassic), and consider these strata as belonging to the Jiulongshan Formation. This is supported by paleontological evidence from conchostracans and insects [44], [56].

The surrounding gymnosperm forests were dominated by Ginkgopsida, Coniferopsida, Lycopods, Sphenopsida, Filicopsida, Cycadopsida [57]. The climate was humid and warm-temperate [58].

The Daohugou beds contain a diverse fauna composed of complete specimens of 19 insect orders, including the three orders of Neuropterida [59], [60], spiders [61], freshwater conchostracans [62], salamanders [63], feathered dinosaurs [64], pterosaurs [65], and mammals [66]. Neuroptera examined include approximately 2000 specimens in at least 15 families. The Osmylidae are most abundant and diverse among neuropterans. Chrysopidae, Grammolingiidae, Saucrosmylidae, Ithonidae, Kalligrammatidae, Psychopsidae, Osmylopsychopidae and Parakseneuridae are common. Berothidae, Mantispidae, Panfiloviidae, Nymphidae, Brongniartiellidae and Mesochrysopidae are rare (VM, QY, pers. obs.).

### Sai-Sagul

Several sites of the Sogul Formation with similar geology and lacking coal accumulation are known as the Say-Sagul locality [ = Shurab 3; = Svodovoe Ruslo]. It is situated in 12 km SW of Shurab in Batken District, Osh Region, Kyrgyzstan. The age of these lacustrine deposits is unclear. It is thought to be the Early-Middle Lias ( = early to middle Early Jurassic) based on paleobotanical data [67], and the late Early Jurassic to the early Middle Jurassic based on the insect assemblage [68]. This territory was south-western part of long Jurassic lake (about 50 km) located near the northern coast of the tropical Tethys Ocean. The surrounding area was covered with wet and warm ginkgoaceous and cycadaceous forests, and apparently characterized by a humid climate.

Fourteen insect orders were recorded from this locality [68]. Neuroptera are represented by 240 specimens, but none were hitherto described. “The abundance of myrmeleontoid-like neuropterans (Ponomarenko, personal communication)” has been reported only [68]. Five neuropteran families are preliminarily identified: Osmylopsychopidae (most abundant neuropterans), Panfiloviidae, Grammolingiidae, Osmylidae, and Parakseneuridae (AK, pers. obs.).

## Results

### Systematic Paleontology

Insecta Linnaeus, 1758

Neuroptera Linnaeus, 1758

### Parakseneuridae fam. nov

urn:lsid:zoobank.org:act:694338EB-2AC3-4CA4-BE7A-2774A61EFC43

#### Type Genus

*Parakseneura* gen. nov.

#### Diagnosis

Large neuropterans (forewing 50–75 mm long) with the following character states: labial palpi stout, relatively short; antennae stout, filiform, apparently much shorter than forewing length; two tibial spurs straight, shorter than basitarsus; claws big, strongly curved; in both wing, humeral veinlet well-developed, strongly recurrent, branched; presumed ScA short, fused with ScP within humeral area; membrane covered with dense, long hairs; RA (or ScP+RA) entering margin well before wing apex; subcostal crossveins numerous; radial crossveins irregularly spaced, not forming gradate series; in the forewing, MP, CuA, CuP dichotomously branched; presumed AA1+2 very short (found in *Parakseneura* gen. nov.); AA3+4, AP1+2, AP3+4 deeply forked; in hind wing, presumed AA1+2 very short (found in *Pseudorapisma* gen. nov.); proximal half of hind wings considerably wider than distal.

#### Occurrence

Middle Jurassic of Dauhugou (Inner Mongolia, China); the Early/Middle Jurassic of Sai-Sagul (Kyrgyzstan).

#### Genera Included

Three genera: *Parakseneura* gen. nov., *Shuraboneura* gen. nov., *Pseudorapisma* gen. nov.

#### Comments

These three genera share similar size and wing venation (e.g., MP, CuA, CuP, AA3+4, and AP1+2 are dichotomously branched; the humeral veinlet is well-developed and strongly recurrent; ScA is present; the nygmata is absent; crossveins are sporadically arranged). Such a combination of character states is known only in a few genera of Kalligrammatidae with generalized venation (e.g., *Protokalligramma* Yang *et al*. [69]). However, these genera is easily distinguished from genera of Kalligrammatidae by other characters, e.g., the presence of the presumed AA1+2 in both wings and the basal sinuate crossvein r-m in the hind wing; relatively scarce crossveins; short palpi. Therefore, the creation of a new family for these three genera is fully justifying.

### Parakseneura Yang, Makarkin & Ren, gen. nov

urn:lsid:zoobank.org:act:D8EF29D6-7593-469B-AEDB-33F62CAF8544

#### Type Species

*Parakseneura nigromacula* sp. nov.

#### Diagnosis

**Figure 1:**
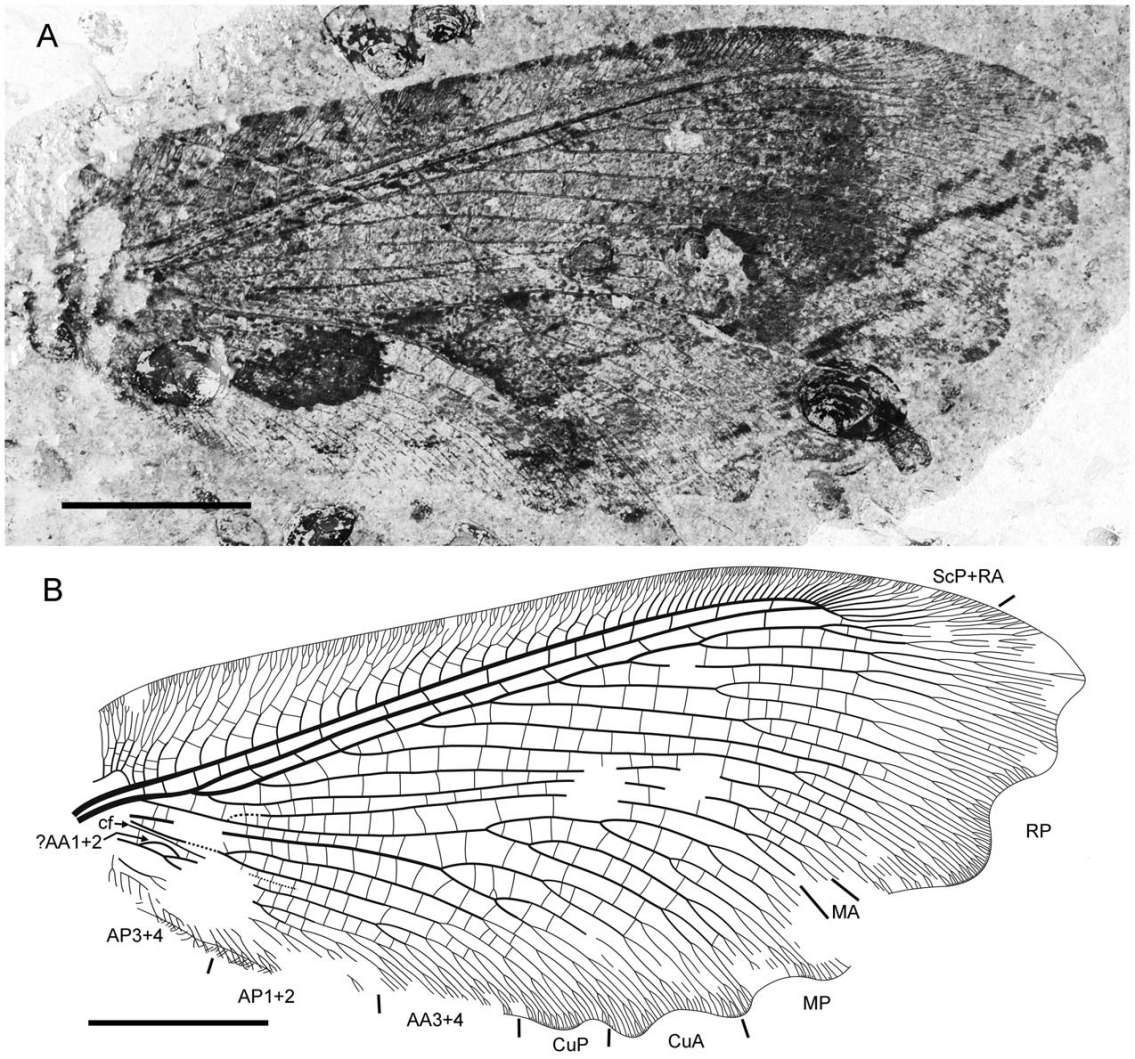
*Parakseneura nigromacula* gen. et sp. nov., holotype CNU-NEU-NN2011009. A, photograph; B, drawing of the forewing venation. Scale bar 10 mm.

In forewing, outer margin undulate (smooth in *Shuraboneura*, *Pseudorapisma*); ScP, RA distally fused (separate in *Shuraboneura*, *Pseudorapisma*); presumed AA1+2 present, very short, fused with AA3+4 forming basal ‘loop’ (Fig. 1, labeled *?AA1*+*2*) (absent in *Pseudorapisma*); in hind wing, basal sinuate crossvein between R and M systems present (absent in *Pseudorapisma*).

#### Species Included

Eleven species: *Parakseneura nigromacula* gen. et sp. nov., *P. undula* gen. et sp. nov., *P. albomacula* gen. et sp. nov., *P. curvivenis* gen. et sp. nov., *P. nigrolinea* gen. et sp. nov., *P. albadelta* gen. et sp. nov., *P. cavomaculata* gen. et sp. nov., *P. inflata* gen. sp. et nov., *P. metallica* gen. et sp. nov., *P. emarginata* gen. et sp. nov., *P. directa* gen. et sp. nov. Also four undetermined specimens are described below as *Parakseneura* sp. inted. A to D.

#### Occurrence

Middle Jurassic (Jiulongshan Formation) of Daohugou (Inner Mongolia, China).

#### Etymology

From the Greek *paraksenos*, strange, and Neuroptera, in reference to unusual morphology of these neuropterans. Gender feminine.

#### Remarks

The forewing venation of different species of the genus is rather similar, but their color pattern strongly varies. Therefore, we mainly use color pattern of the forewing to diagnose the species. Great morphological diversity of hind wings (especially their shape) indicates that the genus is indeed represented by many species. Unfortunately, no complete, articulated fore- and hind wings are preserved; some ‘forewing’ and ‘hind wing’ species may belong to the same species. The ‘hind wing’ species are diagnosed mainly by their wing shape.

### Parakseneura nigromacula Yang, Makarkin & Ren, sp. nov

urn:lsid:zoobank.org:act:FC28DA6A-4A9C-4248-AB93-F65C262D2A4B

#### Diagnosis

Forewing differs from that of other species by specific color pattern including white area in cubital and anal spaces, whose boundaries are not distinctly visible or rounded; hind wing unknown.

#### Description

*Holotype CNU-NEU-NN2011009* (Fig. 1). Forewing 56 mm long as preserved (estimated compete length about 58 mm), 25 mm wide. Costal margin slightly incurved medially, smoothly curved backward apically; outer margin, distal part of hind margin strongly undulate. Trichosors prominent along preserved portions of outer, hind margins. Hairs on membrane cover entire wing, very dense, longer in basal part. Costal space dilated basally, markedly narrowed apically. All subcostal veinlets dichotomously forked; distal subcostal veinlets much more closely spaced than basal. Humeral veinlet well-developed, strongly recurrent, branched. One to three crossveins between 27 proximal subcostal veinlets (including branches of humeral veinlet), forming regular gradate series distally. Subcostal space moderately broad, with widely and quite regularly spaced crossveins. ScP+RA rather long, rather strongly curved toward RP, with four branches; enter margin well before wing apex. RA space rather narrow, nearly as wide as subcostal space; with widely, regularly spaced oblique crossveins. RP with 9 branches, each dichotomously branched distally; RP1 originated near to origin of RP; proximal part of distal branches nearly parallel to hind margin, those of RP1–RP3 directed at some angle to hind margin (divergent). M forked distal to origin of RP1. MA running nearly parallel to RP1, arched, dichotomously branched distally. Anterior trace of MP running parallel to MA; with three deeply dichotomously branches, forked at the middle of the wing. Cu dividing into CuA and CuP very near to wing base (fork not preserved). CuA dichotomously branched; proximal-most fork of CuA somewhat proximal to proximal-most branch of MP. CuP long, dichotomously branched, its proximal-most fork slightly distal to fork of M; next forks of CuP slightly distal to proximal-most fork of CuA. Presumed AA1+2 present, short, fused with A3+4 forming basal anal ‘loop’. AA3+4 long, deeply forked near to wing base, well proximal to proximal-most fork of CuP; two primary branches dichotomously forked distally. Claval fold distinct basally. AP1+2 probably pectinately branched (incompletely preserved). AP3+4 probably pectinate (poorly preserved). Crossveins between stem of RP and posterior trace of AA3+4 relatively dense, irregular, not forming gradate series; absent in area of end-twigging. Wing color in general marmoraceous, variegated with dark and pale areas; undulate narrow strips near outer margin; broad transverse brown band in radial space; proximal dark brown spot in cubital, anal spaces.

**Figure 2:**
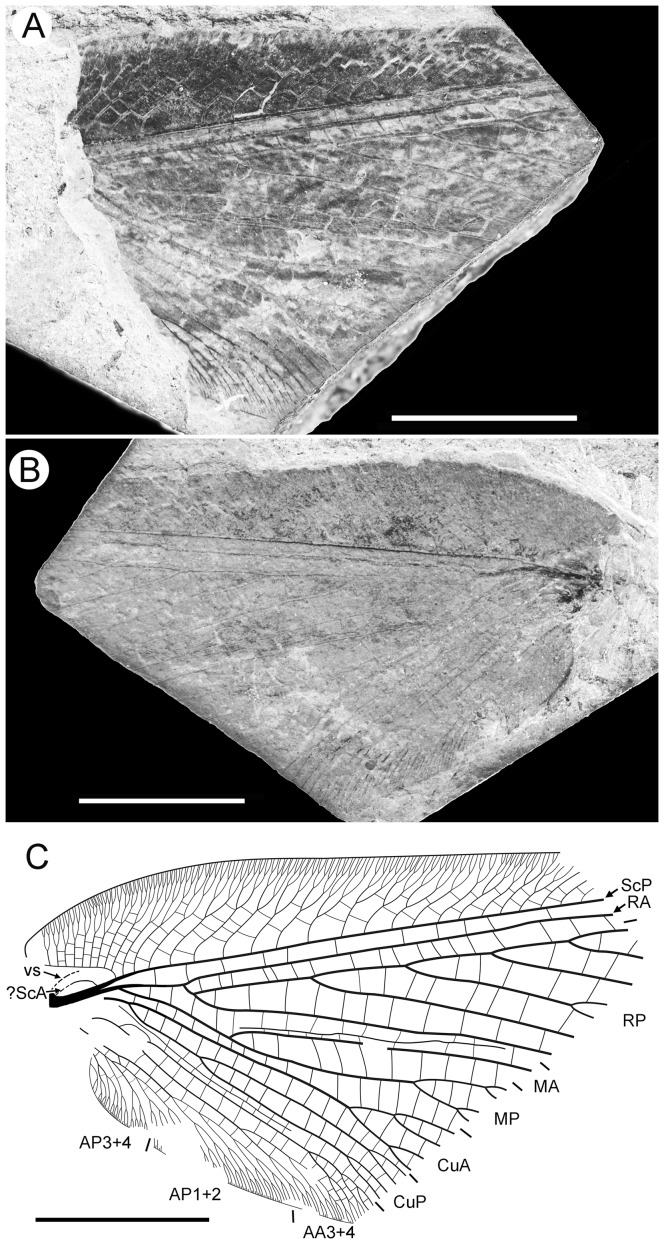
*Parakseneura nigromacula* gen. et sp. nov., specimen CNU-NEU-NN2011026PC. A, part; B, counterpart; C, drawing of the forewing venation. Scale bar 10 mm.

*Specimen CNU-NEU-NN2011026PC* (Fig. 2). Forewing 36 mm long as preserved (estimated complete length about 60 mm). Trichosors present along hind margin, absent along costal margin. Hairs on membrane dense, short (dense, long in costal space). Costal space broad, dilated basally. All preserved subcostal veinlets dichotomously forked. Humeral veinlet well-developed, strongly recurrent and branched, with at least 10 branches. One to five crossveins between proximal subcostal veinlets (including branches of humeral veinlet), not forming regular gradate series. Presumed ScA present, appearing as veinal structure anterior to sclerotized bulge; poorly preserved unknown veinal structure present anterior to presumable ScA. Subcostal space moderately broad, with quite regularly spaced crossveins. RA space slightly wider that subcostal space; with numerous, relatively closely-spaced crossveins. RP with five preserved branches, RP3 deeply forked. RP1 originated near origin of RP. M forked far distal to origin of RP1. MA running nearly parallel to RP1. MP dichotomously branched (preserved part). Cu dividing into CuA and CuP near to wing base. CuA nearly straight before branching, dichotomously branched distally; proximal-most fork of CuA conspicuously proximal to proximal-most fork of MP. CuP long, deeply forked (slightly proximal to fork of M), each branch dichotomously branched (conspicuously distal to proximal-most fork of CuA). Presumed AA1+2 present, short, fused with A3+4 forming basal anal ‘loop’. AA3+4 long, forked relatively far to wing base, well distal to primary fork of CuP; each primary branch dichotomously branched distally. Claval fold distinct. AP1+2 pectinately branched, with dichotomous branches. AP3+4 forked very near wing base; anterior branch dichotomous. Crossveins posterior to stem of RP relatively dense, irregular, not forming gradate series; absent in area of end-twigging. Wing color in general marmoraceous, variegated with dark and pale areas; costal space dark brown; dark brown spot in cubital, anal spaces.

#### Material Examined

Holotype CNU-NEU-NN2011009, an almost complete forewing. Specimen CNU-NEU-NN2011026PC, an incomplete forewing. Both are deposited in CNUB.

#### Occurrence

Middle Jurassic, Bathonian/Callovian, Jiulongshan Formation; Daohugou Village, Shantou Township, Ningcheng County, Inner Mongolia, China.

#### Etymology

From Latin *niger*, black, and *macula* (noun), spot, in reference to large black spot in the forewing.

#### Remarks

The specimen CNU-NEU-NN2011026PC is assigned to this species preliminary because of similar color pattern, unfortunately poorly preserved.

### Parakseneura undula Yang, Makarkin & Ren, sp. nov

urn:lsid:zoobank.org:act:04BECA2C-82BB-40B1-A31E-ADD0E4A4F3CA

#### Diagnosis

Forewing differs from that of other species by posterior portion of wing without large white areas; costal margin of hind wing curved backward after fusion of ScP, RA and running before wing apex as straight line.

#### Description

**Figure 3:**
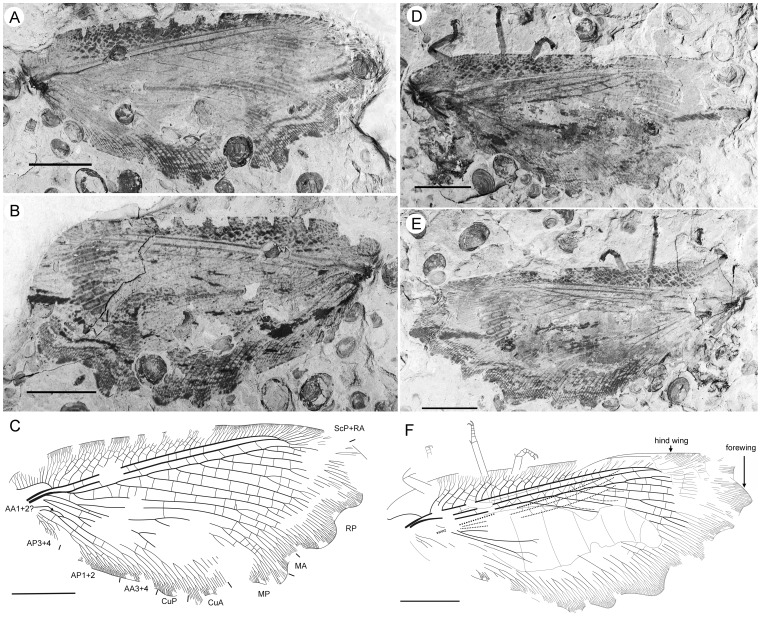
*Parakseneura undula* gen. et sp. nov., holotype CNU-NEU-NN2011030PC (A–C) and paratype CNU-NEU-NN2011031PC (D–F). Photographs of part (A, C), and counterpart (B, E); drawings of the specimen as preserved (C, F). Scale bar 10 mm.

*Holotype* CNU-NEU-NN2011030PC (Figs. 3A–C). Forewing about 57 mm long, about 28 mm wide; moderately broad (length/width ratio 2.04); outer margin, distal hind margin strongly undulate. Trichosors prominent along entire margin (less distinct or absent along proximal part of costal margin). Hairs on membrane not preserved. Costal space dilated at 1/5 proximal length, slightly narrowed basally, markedly narrowed apically. All subcostal veinlets dichotomously branched. Humeral veinlet well-developed, strongly recurrent, heavily branched (at least 12 branches, each forked). One to four crossveins between 20 proximal subcostal veinlets, not forming regular gradate series. Subcostal space moderately broad, with widely-spaced crossveins. ScP, RA distally fused far from wing apex; ScP+RA curved toward RP, with four long branches; enter margin well before wing apex. RA space rather narrow, nearly as wide as subcostal space; with several widely-spaced crossveins, oblique distally. RP with 9 branches, each dichotomously branched distally; RP1 originated near to origin of RP. All branches of RP nearly parallel to hind margin except RP1 directed at some angle to hind margin (divergent). M probably not fused with R basally; forked slightly distal to origin of RP1. MA running parallel to RP1, slightly arched, dichotomously branched distally. Anterior trace of MP running parallel to MA, its proximal-most branch originated slightly proximal to mid-length; branching poorly preserved. Crossveins between stem of RP, posterior trace of MP quite scarce, irregular, mostly widely-spaced, not forming gradate series; absent in area of end-twigging. Cu dividing into CuA and CuP very near to wing base. CuA probably dichotomously branched (branching poorly preserved); proximal-most fork of CuA somewhat proximal to proximal-most branch of MP. CuP dichotomously branched, its primary fork slightly distal to fork of M; next distal forks of CuP slightly distal to proximal-most fork of CuA. Presumed AA1+2 present, short, fused with AA3+4 forming basal anal ‘loop’. AA3+4 long, forked near to wing base, proximal to proximal-most fork of CuP; primary branches parallel to each other, dichotomously forked distally. AP1+2 pectinately branched, with five dichotomous branches. AP3+4 rather short, forked at wing base, each branch dichotomously branched. Wing color in general pale with brown marmoraceous-like pattern in costal space, near outer, hind margins; undulate narrow strips near outer margin; broad transverse light brownish band in radial space proximally prolonged into longitudinal strip; several proximal dark brown spots in cubital, anal spaces.

*Paratype* CNU-NEU-NN2011031PC (Figs. 3D–F). All legs relatively short, with strong curved claws; mid-, hind-legs with short tibial spurs (one preserved on each leg). Abdomen very poorly preserved; no details visible.

Forewing 61 mm long, 25 mm wide, slightly narrower than that of the holotype (length/width ratio 2.44). Preserved venation as in the holotype. Wing apex preserved, sub-acute. Wing pattern very similar to that of the holotype.

Hind wing almost entirely overlapped by forewing. Costal margin slightly incurved at middle; curved backward after fusion of ScP, RA and running before wing apex as straight line. Outer margin just after wing apex incurved. Subcostal space moderately broad. RA space wider than subcostal space.

#### Material Examined

Holotype CNU-NEU-NN2011030PC, a rather well-preserved incomplete forewing. Paratype CNU-NEU-NN2011031PC, an incomplete specimen in lateral aspect. Both specimens are located on single stone 33 mm distant from each other, deposited in CNUB.

#### Occurrence

Middle Jurassic, Bathonian/Callovian, Jiulongshan Formation; Daohugou Village, Shantou Township, Ningcheng County, Inner Mongolia, China.

#### Etymology

From the Latin *undulus*, undulate, in reference to the undulant hind margin of the forewing.

#### Remarks

The holotype and paratype belong to the same species with certainty. They differ mainly in forewing proportions: the forewing of the holotype is slightly broader than of that of the paratypes. This difference may be explained by sexual dimorphism in forewing shape occurring in some Neuroptera; therefore, these specimens may belong to different sexes.

### Parakseneura albomacula Yang, Makarkin & Ren, sp. nov

urn:lsid:zoobank.org:act:59AFD368-E4A9-42AE-9B4D-98612DE457E1

#### Diagnosis

Forewing differs from that of other species by color pattern, predominantly very dark with small pale spots; in hind wing, costal margin strongly curved backward after fusion of ScP and RA; large pale spot in apical portion of radial space.

#### Description (Fig. 4)

**Figure 4:**
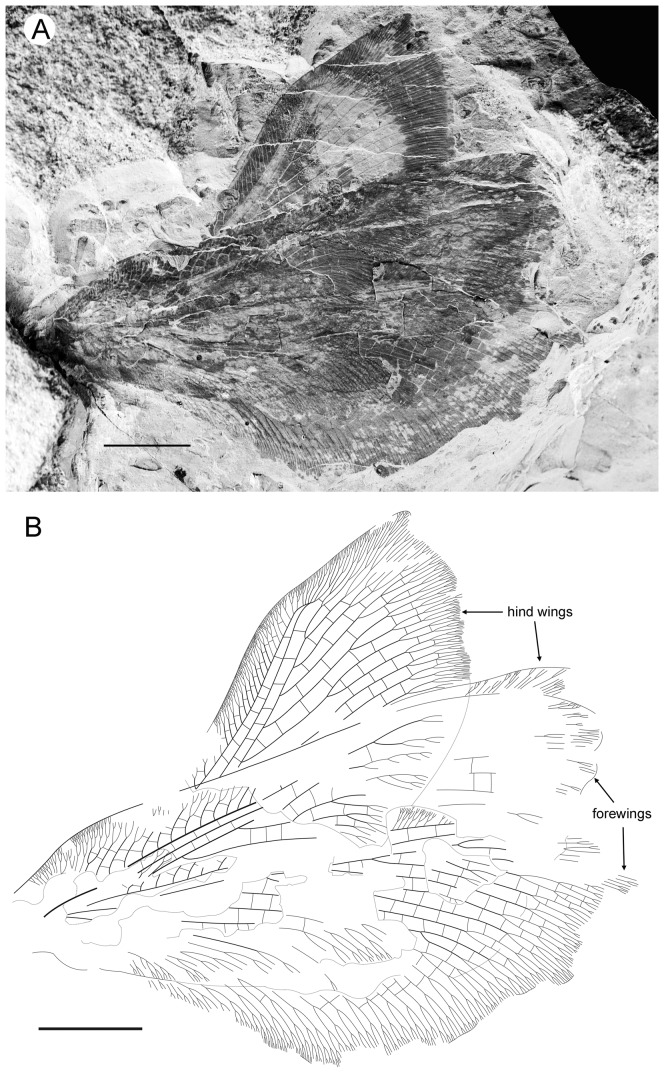
*Parakseneura albomacula* gen. et sp. nov., holotype CNU-NEU-NN2011029. A, photograph; B, drawing of the specimen as preserved. Scale bar 10 mm.

Forewing about 60 mm long, 24.5 mm wide (length/width ratio 2.45), with undulate outer margin (poorly preserved). Trichosors distinct along preserved outer margin. Hairs on membrane dense, quite long in basal portion; near outer margin not so dense as in other parts (probably due to poor preservation); near hind margin dense but not long. Costal space dilated at 1/6, slightly narrowed basally, apically. All preserved subcostal veinlets forked, some dichotomously. Humeral veinlet not preserved. One to two crossveins between subcostal veinlets. Subcostal space moderately broad, with widely-spaced crossveins. RA space as wide as subcostal space, with widely-spaced crossveins. Crossveins posterior to stem of RP rare, irregularly spaced. Color pattern in general dark brown marmoraceous.

Hind wing (apical portion). Costal margin slightly incurved medially, strongly bent backward after fusion of ScP, RA; wing apex sub-acute; outer margin probably excised immediately after wing apex; outer margin slightly undulate. Trichosors prominent along distal part of costal margin (other margins not preserved). Costal space broad. Subcostal veinlets dichotomously branched, each connected by one costal crossvein forming gradate series. ScP+RA relatively short, entering wing margin well before apex, gently bent backward basally, in general incurved, with two-three dichotomous branches. Subcostal space moderately broad distally, with quite regular widely-spaced crossveins. RA space slightly wider than subcostal space, with rare crossveins. Branches of RP dichotomously branched distally, connected with scarce crossveins. Membrane hairs short, dense. Color pattern of apical half in general dark brown with large pale spot in radial space.

#### Material Examined

Holotype CNU-NEU-NN2011029, deposited in CNUB; four incomplete wings partially overlapping.

#### Etymology

From the Latin *albus*, white, and *macula* (noun), spot, in reference to large pale spot in the hind wing.

#### Occurrence

Middle Jurassic, Bathonian/Callovian, Jiulongshan Formation; Daohugou Village, Shantou Township, Ningcheng County, Inner Mongolia, China.

#### Remarks

Costal apical margin of the hind wing of this species is similarly configured to that of *P*. *undula* sp. nov., but the forewing coloration of these species is quite different (i.e., generally pale with scarce dark maculation in *P*. *undula* sp. nov., generally dark with small pale spots in *P. albimacula* sp. nov.).

### Parakseneura curvivenis Yang, Makarkin & Ren, sp. nov

urn:lsid:zoobank.org:act:1D31271A-E777-4F82-9F90-C7B4C3B56833

#### Diagnosis

In hind wing, costal margin strongly bent backward after fusion of ScP and RA, ScP+RA strongly bent toward RP, and large pale spot in apical portion of radial space absent.

#### Description (Fig. 5)

**Figure 5:**
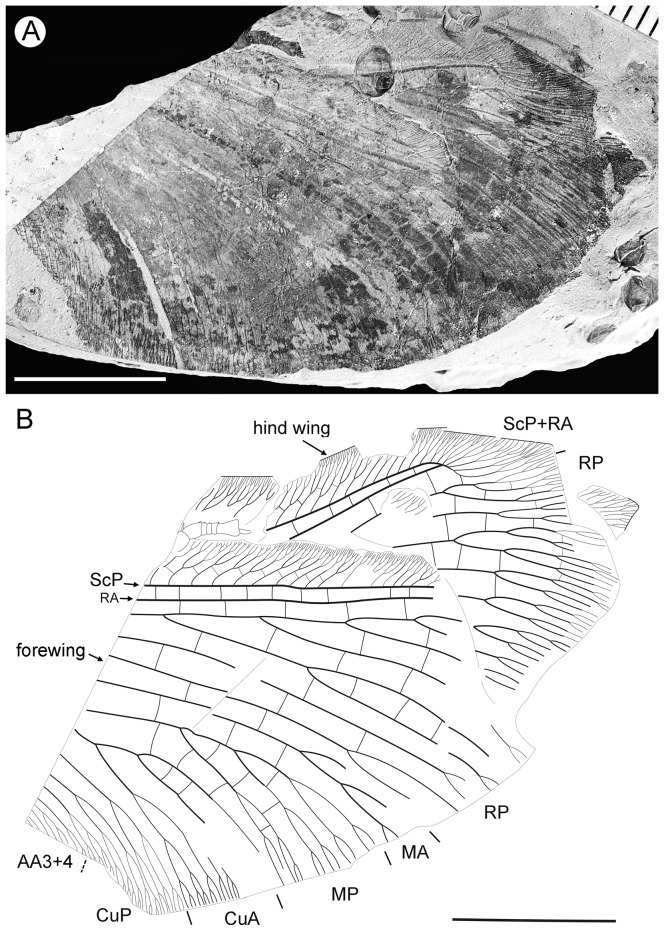
*Parakseneura curvivenis* gen. et sp. nov., holotype CNU-NEU-NN2011021. A, photograph; B, drawing of the specimen as preserved. Scale bar 10 mm.

Tibial apical spurs short, straight. Distal part of tibia and tarsus covered with minute, dense hairs (‘microtrichia’); longer bristles at ventral distal part of 1st to 4th tarsal segments; basitarsus, 5th tarsal segments elongate, nearly equal in length; other three segments of tarsus much shorter, transverse; claws incomplete.

Forewing (central part preserved). Venation similar to *P. undula* sp. nov., differ as follow: costal crossveins arranged in one series; RP with six branches; more rare crossveins. One of proximal-most branch of MA aberrantly reduced. MP with three pectinate branches, of them two proximal dichotomously branched. CuA in general dichotomously branched. Primary fork of MP located markedly more distally than that of CuA. Color pattern poorly-preserved, probably quite similar to that of *P. undula* sp. nov., in general variegated with dark, pale areas.

Hind wing (apical part preserved). Costal margin strongly bent backward after fusion of ScP, RA; wing apex sub-acute; outer margin probably excised immediately after wing apex. Costal space broad. Subcostal veinlets dichotomously branched, some connected by costal crossveins. ScP+RA relatively short, strongly bent backward basally; in general incurved, with four dichotomous branches. Subcostal space quite narrow apically, with regular crossveins. RA space much wider than subcostal space, with scarce crossveins. Branches of RP dichotomously branched distally, connected with scarce crossveins. Color pattern in general variegated with dark, pale areas (probably not completely preserved).

#### Material Examined

Holotype CNU-NEU-NN2011021, deposited in CNUB; fragmentary fore- and hind wings overlapping, and distal part of one leg.

#### Etymology

From the Latin *curvus*, curved, and *vena*, vein, in reference to the vein ScP+RA in the hind wing strongly curved posteriorly.

#### Occurrence

Middle Jurassic, Bathonian/Callovian, Jiulongshan Formation; Daohugou Village, Shantou Township, Ningcheng County, Inner Mongolia, China.

### Parakseneura nigrolinea Yang, Makarkin & Ren, sp. nov

urn:lsid:zoobank.org:act:BFEB3B53-6203-4DB3-8F90-0D25AC9F4896

#### Diagnosis

Forewing color pattern differs from that of other species by the presence of dark longitudinal strip between RP2 and RP3; hind wing unknown.

#### Description

**Figure 6:**
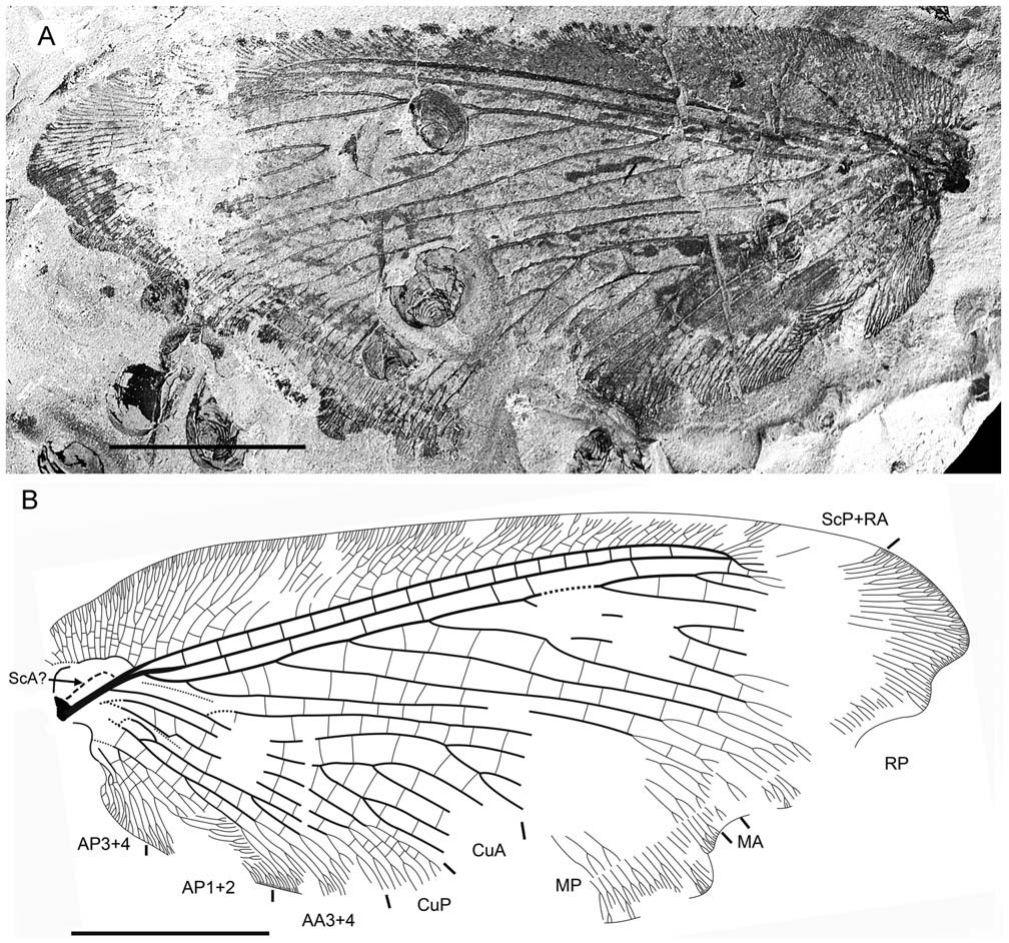
*Parakseneura nigrolinea* gen. et sp. nov., holotype CNU-NEU-NN2011017. A, photograph; B, drawing of the forewing venation. Scale bar 10 mm.

*Holotype CNU-NEU-NN2011017* (Figs. 6A, B). Forewing broad, about 47.3 mm long (as preserved; estimated length about 48.5 mm), 21.5 mm wide (as preserved; estimated width about 23 mm), with undulate outer margin; hind margin probably smooth. Trichosors prominent along hind, outer margins, and costal margin near wing apex. Microtrichia apparently cover entire wing membrane, denser in dark areas, sparser in pale areas probably due to poor preservation. Costal space very broad, dilated proximally then gradually becoming narrower. Subcostal veinlets dichotomously forked, connected by one to four crossveins (including branches of humeral veinlet), not forming regular gradate series. Humeral veinlet well-developed, strongly recurrent, branched, with at least 12 branched. Presumed ScA present. Subcostal space moderately broad, with quite dense, regularly spaced crossveins. ScP+RA rather strongly curved to RP, with two branches. RA space slightly wider than subcostal space; with rare, irregularly spaced crossveins. RP with 7 branches before fusion of ScP, RA; some deeply forked. RP1 originated relatively far to origin of RP; RP1, RP2 converging in middle. M forked distal to origin of RP1. MA few-branched distally. MP sinuous, probably dichotomously branched (incompletely preserved). Cu dividing into CuA and CuP near to wing base. CuA dichotomously branched; proximal-most fork of CuA well proximal to proximal-most branch of MP. CuP deeply forked, each branch dichotomously branched distally. Presumed AA1+2 present, relatively long, fused with AA3+4. AA3+4 long, forked near to wing base, proximal to fork of M; each branch deeply dichotomously forked. AP1+2 probably pectinately branched (incompletely preserved). Branching of AP3+4 unclear, in general dichotomous. Crossveins posterior to RP relatively rare, irregular, not forming gradate series. Wing color in general pale with brownish pattern in costal space, near outer, hind margins; apex darker; dark brown large spot in cubital, anal spaces; pale spot narrowly margined with brown at distal portions of RP2-MA; brown longitudinal strip between proximal portions of RP2, RP3.

**Figure 7:**
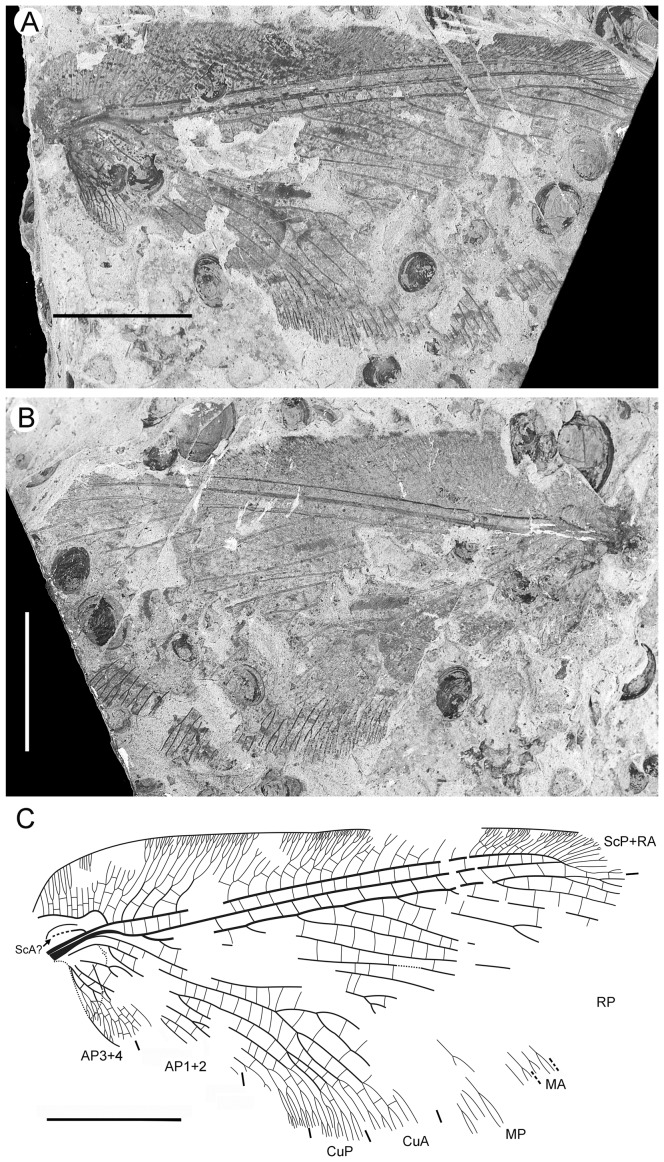
*Parakseneura nigrolinea* gen. et sp. nov., paratype CNU-NEU-NN2011011PC. A, part; B, counterpart; C, drawing of the forewing venation Scale bar 10 mm.

*Paratype CNU-NEU-NN2011011PC* (Figs. 7A–C). Forewing 43.2 mm long as preserved (estimated complete length about 50–53 mm), 23.3 mm wide as preserved (estimated complete width about 24–25 mm). Trichosors not detected along costal margin. Hairs on membrane cover entire wing, longer in humeral area. Costal space dilated basally, markedly narrowed apically. All subcostal veinlets dichotomously forked, connected by one to three crossveins (including branches of humeral veinlet), not forming regular gradate series. Humeral veinlet well developed, sinuous, strongly recurrent, branched. Presumed ScA partly preserved; unknown veinal structure anterior to it well developed. Subcostal space moderately broad, with dense and quite regularly spaced crossveins. ScP+RA curved toward RP, with four branches. RA space slightly wider than subcostal space; with rather regularly spaced crossveins. RP with 8 branches; RP1 originated near to origin of RP; RP1, RP2 converging in middle. Fork of M not preserved. Preserved part of MA few-branched distally. MP sinuous, probably dichotomously branched (incompletely preserved). Cu dividing into CuA and CuP near to wing base. CuA dichotomously branched; proximal-most fork of CuA well proximal to proximal-most branch of MP. CuP deeply forked, each branch dichotomously branched distally. Presumed AA1+2 not detected. AA3+4 long, forked near to wing base, slightly distal to Cu; each branch deeply dichotomously forked. AP1+2 probably pectinately branched (incompletely preserved). AP3+4 forked very near wing base; each branches dichotomous). Jugal lobe large (turned-up as preserved). Crossveins posterior to RP relatively dense, irregular, not forming gradate series. Wing color in general pale with brownish marmoreous pattern in costal space; dark brown large spot in cubital, anal spaces; brown longitudinal strip between proximal portions of RP2, RP3.

#### Material Examined

Holotype CNU-NEU-NN2011017, a well-preserved almost complete forewing. Paratype CNU-NEU-NN2011011PC, a well-preserved incomplete forewing. Both are deposited in CNUB.

#### Occurrence

Middle Jurassic, Bathonian/Callovian, Jiulongshan Formation; Daohugou Village, Shantou Township, Ningcheng County, Inner Mongolia, China.

#### Etymology

From Latin *niger*, black, and *linea* (noun), line, in reference to dark stripe between radial branches in the forewing.

#### Remarks

The assignment of these two specimens to the same species is undoubted and supported by very similar forewing venation and color pattern.

### Parakseneura albadelta Yang, Makarkin & Ren, sp. nov

urn:lsid:zoobank.org:act:5F8EC127-48AB-406E-B634-B9DC5DA48CF3

#### Diagnosis

Forewing color pattern differs from that of other species by the presence of distinct white area in cubital and anal spaces resembling the Greek letter delta.

#### Description

**Figure 8:**
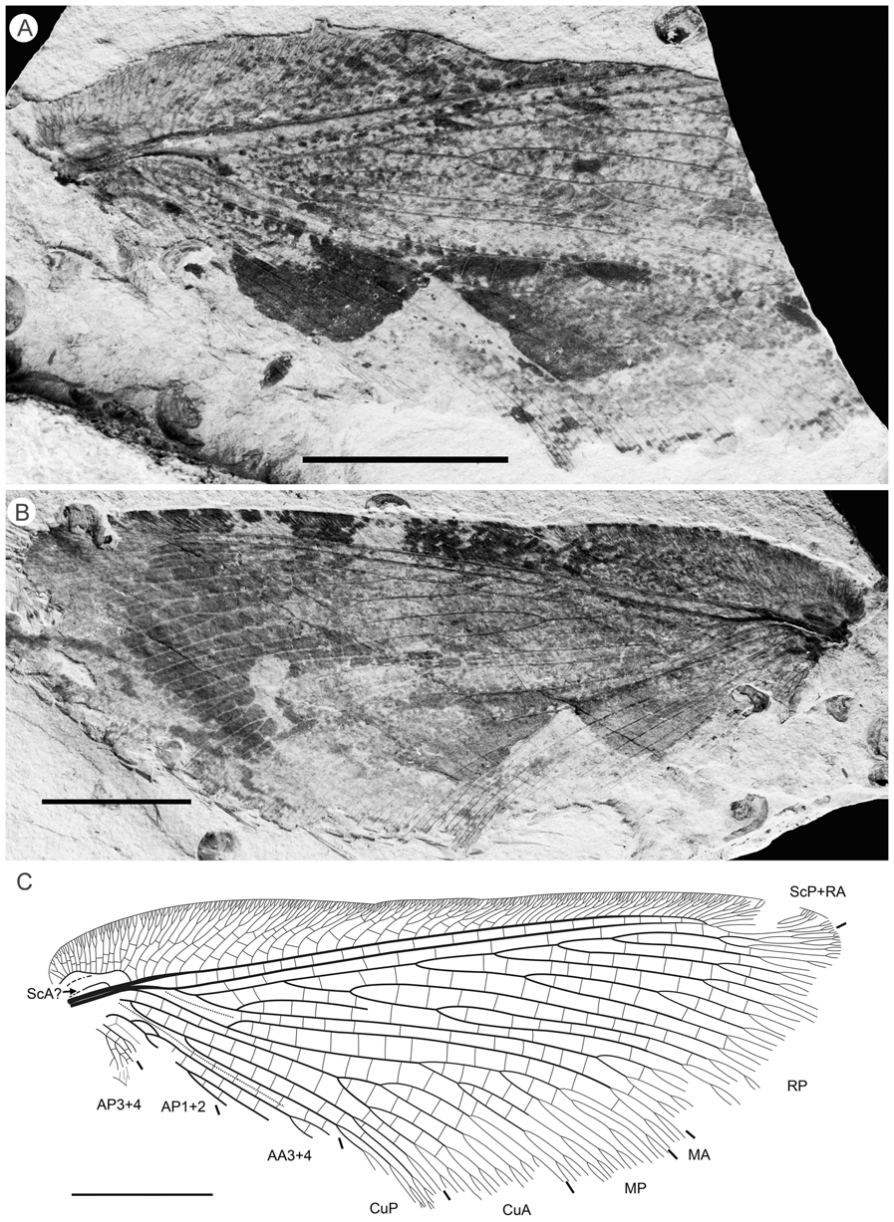
*Parakseneura albadelta* gen. et sp. nov., holotype CNU-NEU-NN2011015PC. A, part; B, counterpart; C, drawing of the forewing venation. Scale bar 10 mm.

*Holotype CNU-NEU-NN2011015PC* (Figs. 8A–C). Forewing 57 mm long as preserved (estimated compete length about 58–59 mm), 22 mm wide (as preserved). Costal margin slightly incurved medially, smoothly bent to backward apically; outer, hind margins not preserved. Trichosors preserved along costal margin. Entire wing membrane covered with hairs, dense and long in basal and costal part, shorter in other region. Costal space dilated basally, narrowed apically. All subcostal veinlets dichotomously forked. Humeral veinlet well developed, strongly recurrent and branched, with at least 15 branches. One to three crossveins between 36 proximal subcostal veinlets (including branches of humeral veinlet), not forming regular gradate series. Presumed ScA well preserved; unknown veinal structure present, poorly preserved. Subcostal space relatively narrow, with numerous, quite regularly spaced crossveins. ScP+RA rather long, slightly curved toward RP, with two long branches. RA space rather narrow, nearly as wide as subcostal space; with widely spaced crossveins. RP with seven branches, three proximal-most of these deeply forked (anterior branch of RP1 anomalously short). M forked far distal to origin of RP1. MA running nearly parallel to RP1, dichotomously branched distally. Anterior trace of MP running parallel to MA; two deep pectinate branches, each dichotomously branched. Cu dividing into CuA and CuP very near to wing base. CuA straight before branching, dichotomously branched; proximal-most fork of CuA conspicuously proximal to proximal-most branch of MP. CuP long, deeply forked (conspicuously proximal to fork of M), each branch dichotomously branched (conspicuously distal to proximal-most fork of CuA). Presumed AA1+2 present, short. AA3+4 long, forked near to wing base proximal to primary fork of CuP; posterior branch deeply forked. Claval fold distinct proximally. AP1+2 fragmentary preserved. AP3+4 probably pectinate (poorly preserved). Crossveins posterior to stem of RP relatively dense, irregular, not forming gradate series; absent in area of end twigging. Wing color in general marmoraceous, variegated with pale and dark areas; two dark brown (blackish) stops in cubital, anal spaces, between which pale triangle spot near hind margin; broad transverse brown band in radial space; pale spot in proximal, posterior part of it.

**Figure 9:**
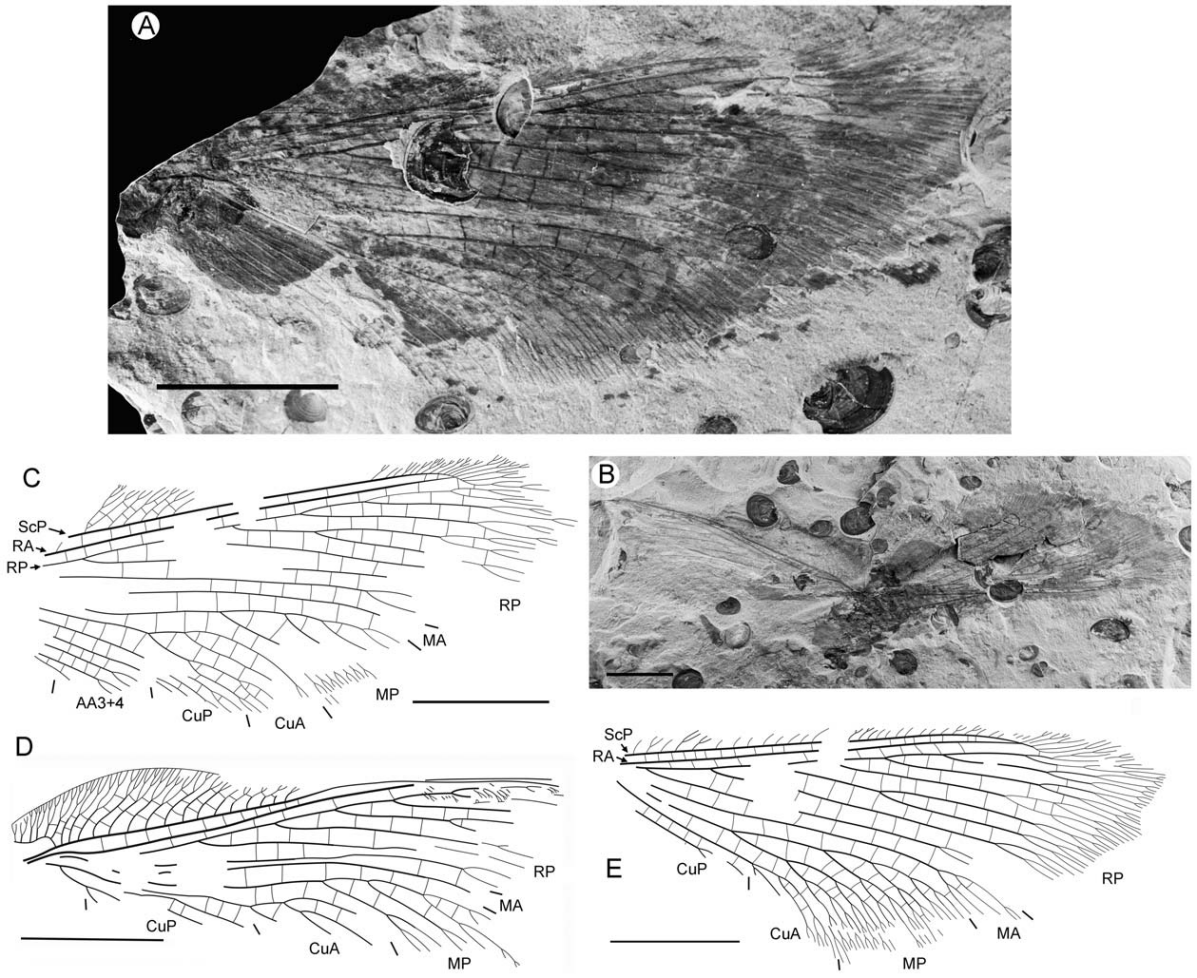
*Parakseneura albadelta* gen. et sp. nov., paratype CNU-NEU-NN2011022PC. A, photograph of part; B, photograph of counterpart; C–E, drawings of the venation: right forewing (C), left forewing (converted to the right) (D), right hind wing (E). Scale bar 10 mm.

*Paratype CNU-NEU-NN2011022PC* (Figs. 9A–E). Body very poorly preserved, no detail detected.

Forewing about 60 mm long (estimated from two incomplete forewings). Wing margin not preserved, except proximal part of costal margin. Membrane hairs in apical region visible, quite dense but not long. Costal space dilated proximally. All preserved subcostal veinlets dichotomously forked. Humeral veinlet well developed, strongly recurrent and branched, with at least 6 branches. One to three crossveins between proximal subcostal veinlets (including two branches of humeral veinlet), not forming regular gradate series. Subcostal space relatively broad, with irregularly spaced crossveins. ScP+RA markedly curved toward RP, with two-three long branches. RA space rather narrow, nearly as wide as subcostal space; with widely spaced crossveins. RP with six branches, each dichotomously branched distally (RP4 deeply in both wings). MA incompletely preserved, probably branched only distally. Anterior trace of MP running parallel to MA, with four pectinate branches, at least three of these dichotomously branched. CuA dichotomously branched; proximal-most fork of CuA markedly proximal to proximal-most branch of MP. CuP deeply forked, both dichotomously branched. AP3+4 fragmentarily preserved. Crossveins between stem of RP and posterior trace of AA3+4 relatively dense, irregular, not forming gradate series. Wing color similar to holotype; distal pale spot narrowly margined with dark.

Hind wing 47 mm long as preserved (estimated complete length about 54–57 mm), 18 mm wide as preserved (estimated complete width about 20 mm). Margins not preserved. ScP, RA fused. ScP+RA in general incurved, with four long veinlets. Subcostal space moderately broad, with relatively dense crossveins. RA space nearly as wide as subcostal space, with quite regularly spaced oblique crossveins. RP with 7 widely spaced branches proximal to pterostigmal region; each branch dichotomously branched distally (RP3 deeply); RP1 originated near origin of RP. MA dichotomously branched distally. MP profusely branched, pectinate, its anterior trace and three branches dichotomously branched distally. CuA relatively shallowly, dichotomously branched. CuP fragmentary preserved. Crossveins posterior to stem of RP rare, irregularly spaced. Color pattern unclear, masked by forewing pattern.

#### Material Examined

Holotype CNU-NEU-NN2011015PC, a well-preserved almost complete forewing. Paratype CNU-NEU-NN2011022PC, an incomplete, quite poorly preserved specimen, with right wings overlapping, and left forewing outspread. Both are deposited in CNUB.

#### Etymology

From the Latin *albus*, white, and *delta*, Greek letter delta (Δ), in reference to big white spot near the hind margin of the forewing resembling this Greek letter.

#### Occurrence

Middle Jurassic, Bathonian/Callovian, Jiulongshan Formation; Daohugou Village, Shantou Township, Ningcheng County, Inner Mongolia, China.

#### Remarks

The assignment of the holotype and paratype to the same species is undoubted and supported by very similar forewing venation and color pattern.

### Parakseneura cavomaculata Yang, Makarkin & Ren, sp. nov

urn:lsid:zoobank.org:act:66270FD8-F0D4-4367-9EA0-B93EF99D24F1

#### Diagnosis

Forewing color pattern differs from that of other species by evenly pale posterior portion of wing, without distinct black spots, and the presence of two pale elongate spots narrowly margined with dark.

#### Description

**Figure 10:**
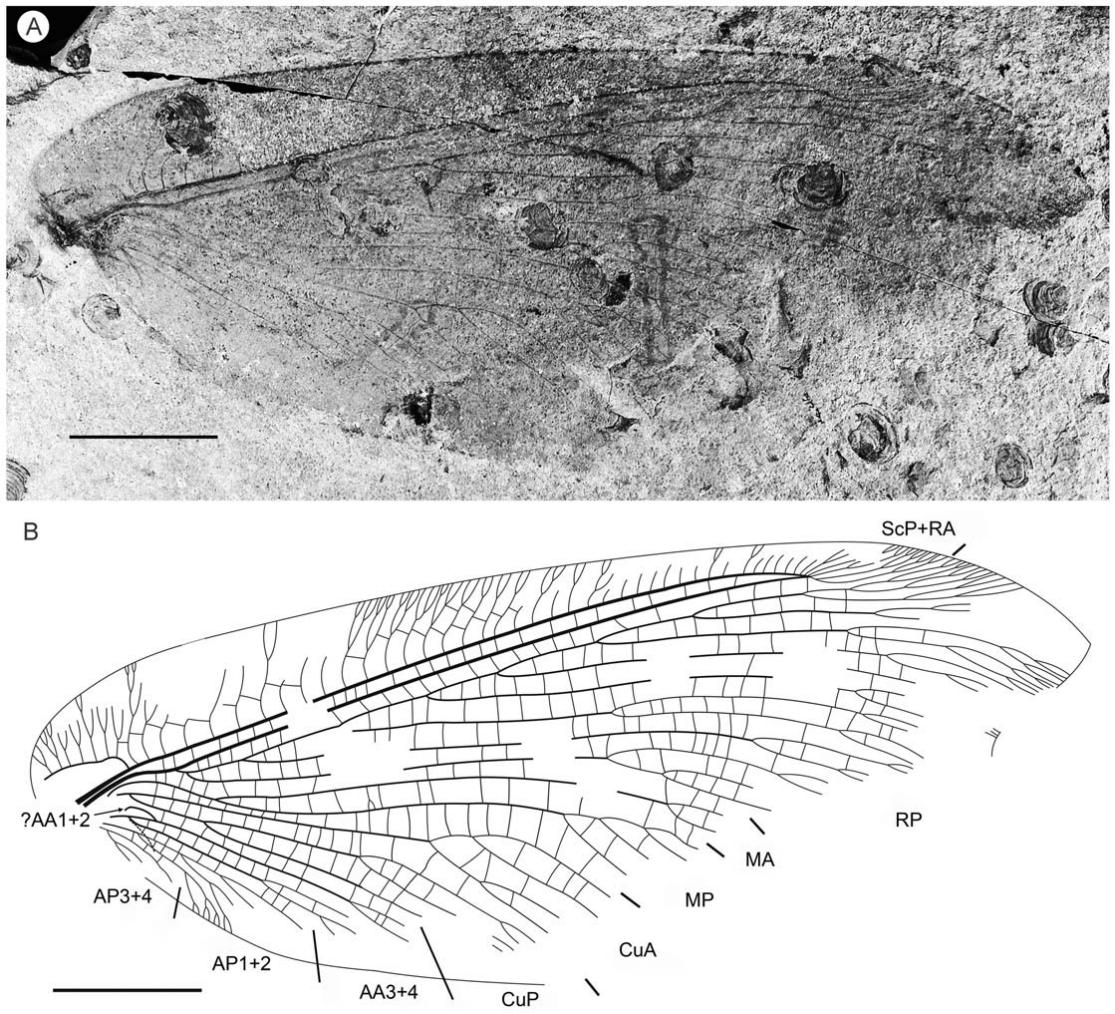
*Parakseneura cavomaculata* gen. et sp. nov., holotype CNU-NEU-NN2011008. A, photograph; B, drawing of the forewing venation. Scale bar 10 mm.

*Holotype CNU-NEU-NN2011008* (Figs. 10A, B). Forewing elongate, 71.6 mm long, about 28 mm wide (length/width ratio 2.56), with acute apex; outer margin probably undulate (poorly preserved), hind margin smooth. Trichosors indistinct. Hairs on veins, membrane not visible, except in humeral area (mainly on humeral plate). Costal space strongly dilated basally, narrowed apically. All subcostal veinlets forked, some dichotomously. Humeral veinlet well developed, strongly recurrent, branched with at least seven forked branches. Costal crossveins form one gradate series. Subcostal space moderately narrow, with numerous crossveins. ScP, RA distally fused far from wing apex; ScP+RA curved toward RP, with three very long branches; enter margin well before wing apex. RA space nearly as wide as subcostal space, with numerous crossveins. RP originated relatively close to wing base, with 9 very oblique branches dichotomously (some deeply) branched distally. RP1 originated near origin of RP. M basally not fused with R, forked distal to origin of RP1. MA slightly sinuous, primary fork in distal position (branching not preserved). MP sinuous, pectinate, with three preserved branches. Cu dividing into CuA and CuP near wing base. CuA straight before branching, pectinate with two long branches. CuP deeply forked (at level of fork of M), its anterior branch forked proximal to origin of proximal-most branch of CuA. Presumed AA1+2 present, short, terminating on AA3+4. AA3+4 deeply forked near wing base; both branches parallel to each other, dichotomously branched distally. AP1+2 pectinate, with three branches, at least two of them dichotomously branched. AP3+4 deeply forked. Jugal lobe apparently large (part of turn-up hind margin preserved). Crossveins posterior to stem of RP relatively dense, irregularly spaced. Color pattern brownish, without black spots; two pale transverse spots narrowly margined with dark: elongate in radial-medial space in distal wing portion, and shorter at origin of proximal-most branch of CuA.

**Figure 11:**
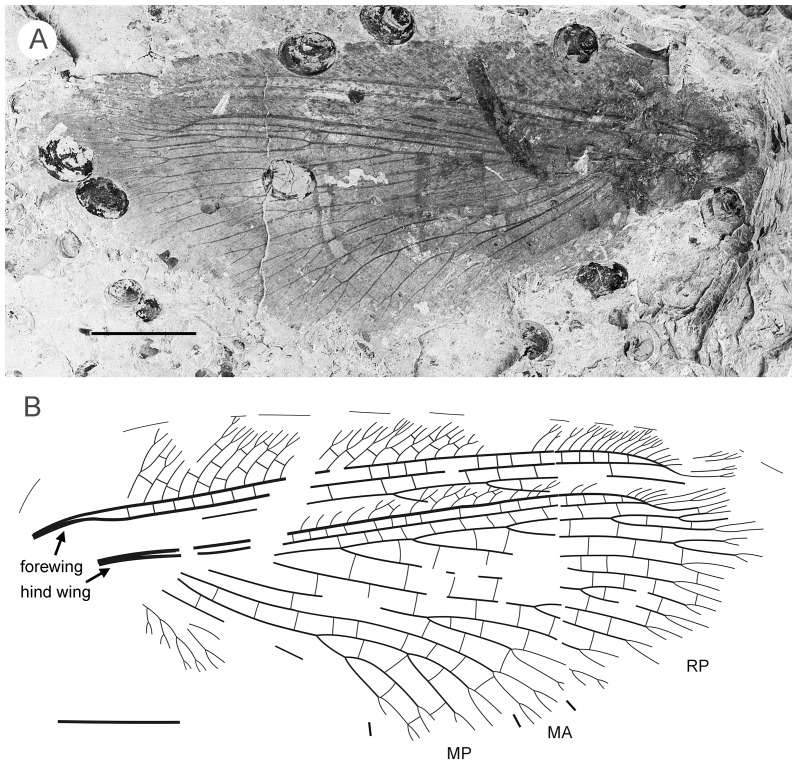
*Parakseneura cavomaculata* gen. et sp. nov., specimen CNU-NEU-NN2011007. A, photograph; B, drawing of the venation. Scale bar 10 mm.

*Specimen CNU-NEU-NN2011007* (Figs. 11A, B). Forewing (only anterior part well preserved) more than 65 mm long. Costal space broad, dilated proximally. Subcostal veinlets dichotomously branched, connecting by one-two crossveins in proximal half of wing. ScP+RA slightly bent toward RP in its basal part; with two long branches. Subcostal, RA spaces nearly equal in width, with quite numerous crossveins. Color pattern unclear, brownish, probably without black spots; elongate transverse pale spot narrowly margined with dark in radial-medial space in distal wing portion, and shorter at origin of proximal-most branch of CuA.

Hind wing more than 55 mm long. Margins not preserved. At least some subcostal veinlets forked, with several preserved crossveins. ScP+RA slightly bent toward RP in basal part; with two long branches. Subcostal space slightly wider than RA space; both with quite numerous crossveins. RP with 6 branches before fusion of ScP, RA, all dichotomously branched in distal part, most quite deeply. MA dichotomously branched distally. MP deeply dichotomously branched. Cubital, anal veins fragmentarily preserved. Crossveins posterior to stem of RP rare, irregularly spaced. Color pattern unclear, without black spots.

#### Material Examined

Holotype CNU-NEU-NN2011008, deposited in CNUB; a nearly complete well-preserved forewing. Specimen CNU-NEU-NN2011007, deposited in CNUB; a nearly complete, quite poorly preserved fore- and hind wings overlapped.

#### Etymology

From the Latin *cavus*, hollow, and *maculata*, spotted, in reference to two pale forewing spots narrowly margined with dark.

#### Occurrence

Middle Jurassic, Bathonian/Callovian, Jiulongshan Formation; Daohugou Village, Shantou Township, Ningcheng County, Inner Mongolia, China.

#### Remarks

The wings of the specimen CNU-NEU-NN2011007 are incomplete and poorly preserved. However, its forewing color pattern is similar to that of the holotype, and this specimen is preliminary assigned to *P. cavomaculata* sp. nov.

### Parakseneura inflata Yang, Makarkin & Ren, sp. nov

urn:lsid:zoobank.org:act:1AE164D6-B519-49AF-B58F-74B11FCF6514

#### Diagnosis

Forewing unknown; costal space of the hind wing strongly dilated (swollen) apically.

#### Description (Fig. 12)

**Figure 12:**
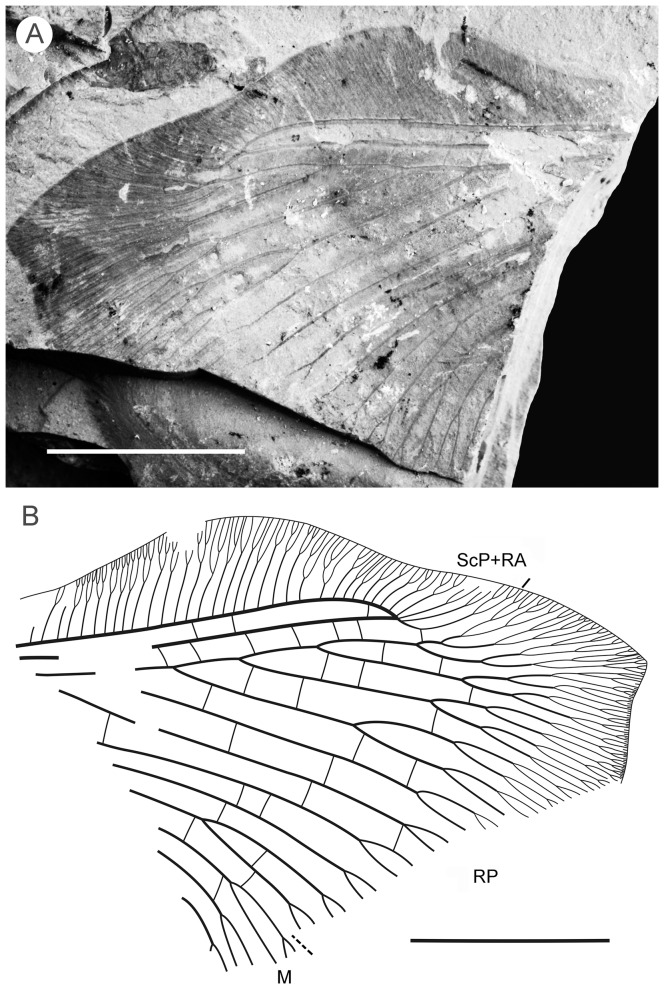
*Parakseneura inflata* gen. et sp. nov., holotype CNU-NEU-NN2011010. A, photograph; B, drawing of the hind wing venation. Scale bar 10 mm.

Hind wing 33 mm long as preserved (estimated complete length about 45–55 mm), with pointed apex; outer margin excised immediately posterior to apex. Trichosors prominent along outer margin, not distinct along costal margin. Costal space moderately broad, extremely dilated (swollen) apically. All preserved subcostal veinlets, veinlets of ScP+RA dichotomously branched. Costal crossveins not detected. ScP, RA fused. ScP+RA short, proximally bent toward RP, in general incurved; enter margin well before wing apex, with 3 long veinlets. Subcostal space moderately broad, with rare crossveins. RA space nearly as broad as subcostal space, with quite regular crossveins. Branches of RP dichotomously branched distally. Crossveins posterior to stem of RP rare. Color of wing apical portion in general brownish, probably paler in area of primary forking of branches of RP.

#### Material Examined

Holotype CNU-NEU-NN2011010, deposited in CNUB; a well-preserved apical portion of hind wing.

#### Etymology

From the Latin *inflatus*, swollen, in reference to the strongly dilated apical portion of the costal space.

#### Occurrence

Middle Jurassic, Bathonian/Callovian, Jiulongshan Formation; Daohugou Village, Shantou Township, Ningcheng County, Inner Mongolia, China.

#### Remarks

It is unknown with which ‘forewing’ species this hind wing may be associated. It does not belong surely to *Parakseneura undula* sp. nov., *P. albomacula* sp. nov., *P. curvivenis* sp. nov., *P. cavomaculata* sp. nov. as their hind wings are known (at least their costal margin) and strongly differ.

### Parakseneura metallica Yang, Makarkin & Ren, sp. nov

urn:lsid:zoobank.org:act:EF0115E9-FCF5-4112-A1C2-3F3867916EA7

#### Diagnosis

Forewing unknown; hind wing somewhat metallic in color, i.e., shining with a faint tinge of blue; costal margin slightly convex.

#### Description (Fig. 13)

**Figure 13:**
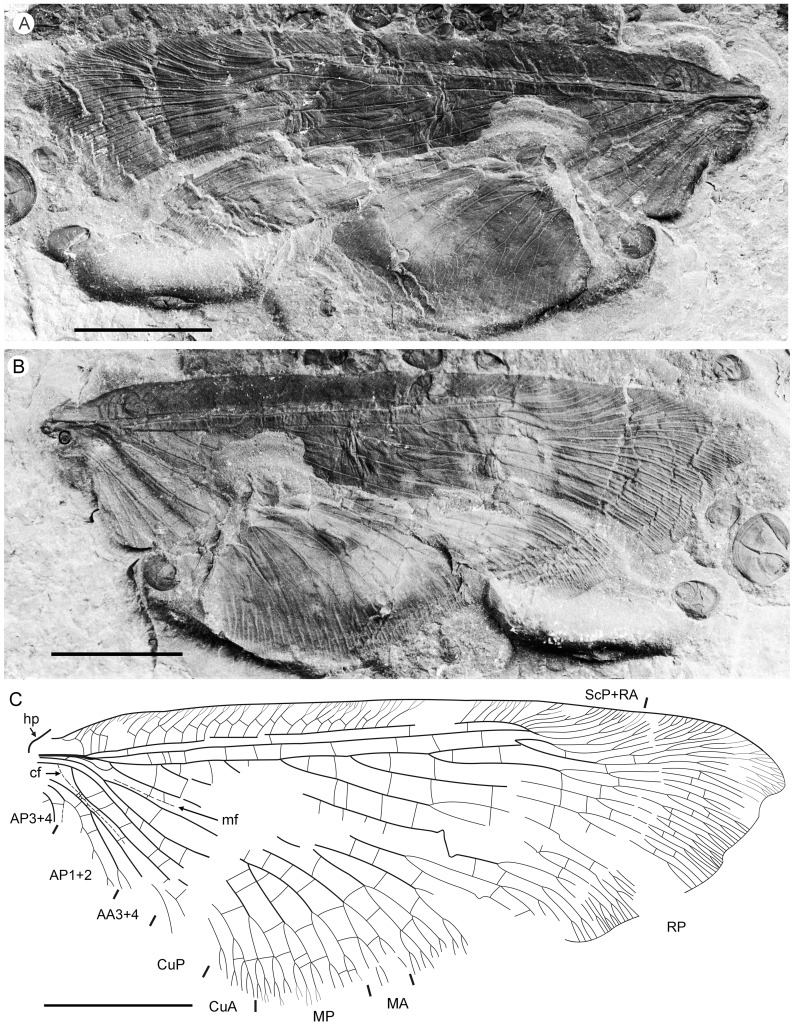
*Parakseneura metallica* gen. et sp. nov., holotype CNU-NEU-NN2011019PC. A, photograph of part; B, photograph of counterpart; C, drawing of the hind wing venation. Scale bar 10 mm.

Hind wing 53.3 mm long, 22 mm wide. Costal margin slightly convex; wing apex obtuse; outer margin excised immediately posterior to apex, posteriorly slightly undulate; hind margin not preserved. Trichosors prominent along outer margin, costal margins. Humeral plate well developed, covered with many fine hairs. ScA not preserved. Costal space equally moderately broad. All preserved subcostal veinlets forked once, few dichotomously branched; humeral veinlet recurrent, branched. Costal crossveins forming one series in proximal half of wing. ScP, RA fused. ScP+RA relatively short, proximally bent toward RP, in general incurved; enter margin well before wing apex, with two long branched veinlets. Subcostal space moderately broad, with very scarce preserved crossveins. RA space broader than subcostal space, with scarce irregularly spaced crossveins. RP with 7 widely spaced branches proximal to pterostigmal region; each branch profusely dichotomously branched distally; RP1 originated near origin of RP. Basal r-m brace between R, M systems long, strongly sinuous. Medial fold distinct in proximal part of wing. M forked slightly distal to origin of RP1. MA dichotomously branched distally. MP pectinately branched, its anterior trace and two branches dichotomously branched distally. Cu forked near wing base. CuA, CuP relatively shallowly, dichotomously branched. Claval fold distinct. AA3+4 deeply forked. AP1+2 pectinate, probably with four branches. AP3+4 incompletely preserved. Crossveins posterior to stem of RP rare, irregularly spaced. Color pattern in general dark brown, with paler regions; veins appear dark bluish.

#### Material Examined

Holotype CNU-NEU-NN2011019P/C, deposited in CNUB; a nearly complete well-preserved hind wing.

#### Etymology

From Latin *metallicus*, metallic, in reference to the metallic color of the wing (a faint tinge of blue and shining).

#### Occurrence

Middle Jurassic, Bathonian/Callovian, Jiulongshan Formation; Daohugou Village, Shantou Township, Ningcheng County, Inner Mongolia, China.

#### Remarks

The wing is broken; the posterior portion is dislocated. This hind wing may not be assigned to any species whose hind wings (or their costal margin) are known.

### Parakseneura emarginata Yang, Makarkin & Ren, sp. nov

urn:lsid:zoobank.org:act:C80B253E-B114-45CB-B070-EAB8AE203603

#### Diagnosis

Forewing unknown; costal margin of hind wing markedly excised in region of fusion of ScP and RA.

#### Description (Fig. 14)

**Figure 14:**
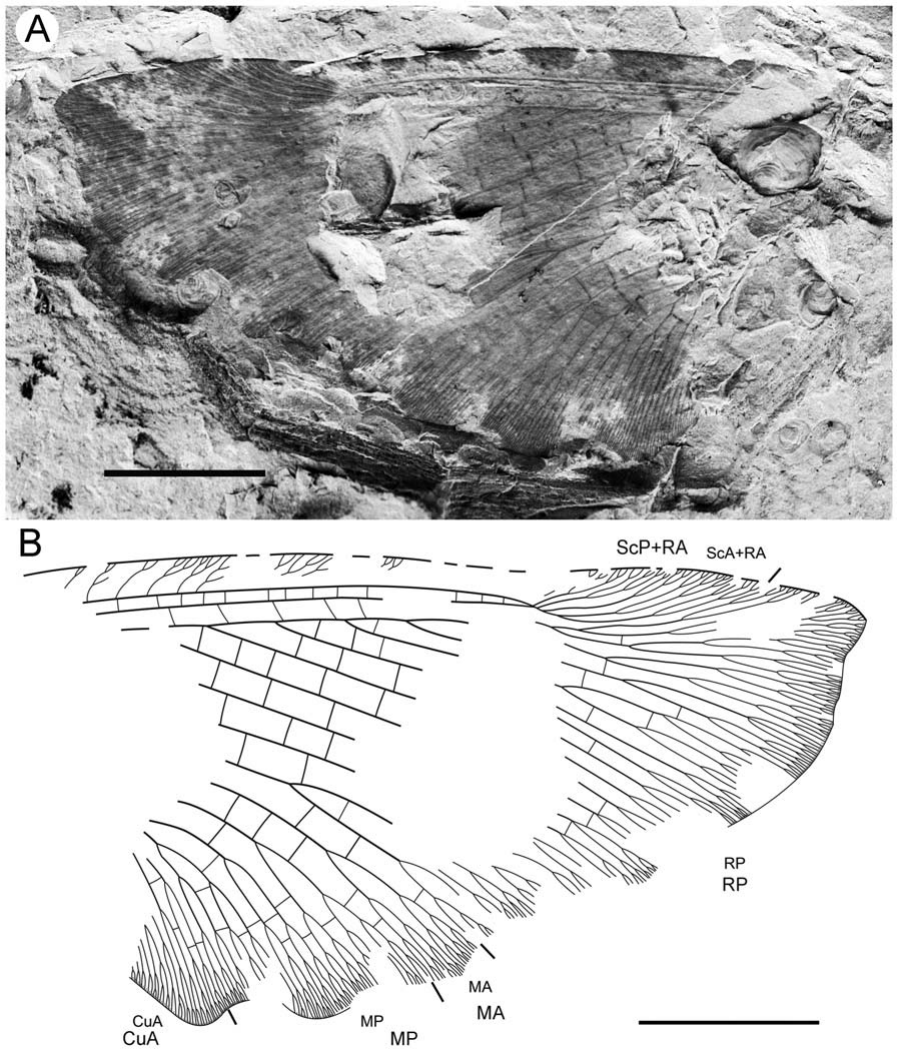
*Parakseneura emarginata* gen. et sp. nov., holotype CNU-NEU-NN2011024. A, photograph; B, drawing of the hind wing venation. Scale bar 10 mm.

Hind wing broad, 48 mm as preserved (estimated complete length about 55 mm), 26 mm wide. Costal margin markedly excised in region of fusion of ScP, RA; apically only slightly curved backward; outer margin slightly undulate, excised immediately posterior to apex; hind margin strongly undulate. Outer, hind margins with distinct trichosors. Membrane hairs dense, rather long. Costal space moderately broad. Preserved veinlets of ScP dichotomously forked; no crossveins detected. ScP, RA fused; ScP+RA gently curved toward RP, entering margin well before wing apex; ScP+RA, its four long branches dichotomously forked. Subcostal space narrow, with regularly spaced crossveins. RA space relatively narrow, with rare oblique crossveins. RP with 10 branches; RP1 quite deeply dichotomously branched. MA dichotomously branched distally. MP deeply dichotomously branched. CuA fragmentarily preserved. Crossveins posterior to RP scarce, irregularly spaced. Color pattern of distal two thirds brownish, with following pale spots: relatively small near margins, one large transverse just proximal to ScP, RA fusion; most probably wing proximally pale.

#### Material Examined

Holotype CNU-NEU-NN2011024, deposited in CNUB; an incomplete hind wing.

#### Etymology

From Latin *emarginatus*, emarginate, in reference to the costal margin of the hind wing markedly excised.

#### Occurrence

Middle Jurassic, Bathonian/Callovian, Jiulongshan Formation; Daohugou Village, Shantou Township, Ningcheng County, Inner Mongolia, China.

#### Remarks

This hind wing may not be assigned to any species whose hind wings (or their costal margin) are known.

### Parakseneura directa Yang, Makarkin & Ren, sp. nov

urn:lsid:zoobank.org:act:A8F0D75D-AABB-420D-8A97-5DB82D5BF993

#### Diagnosis

Forewing unknown; costal margin of hind wing straight; crossveins narrowly margined with pale.

#### Description (Fig. 15)

**Figure 15:**
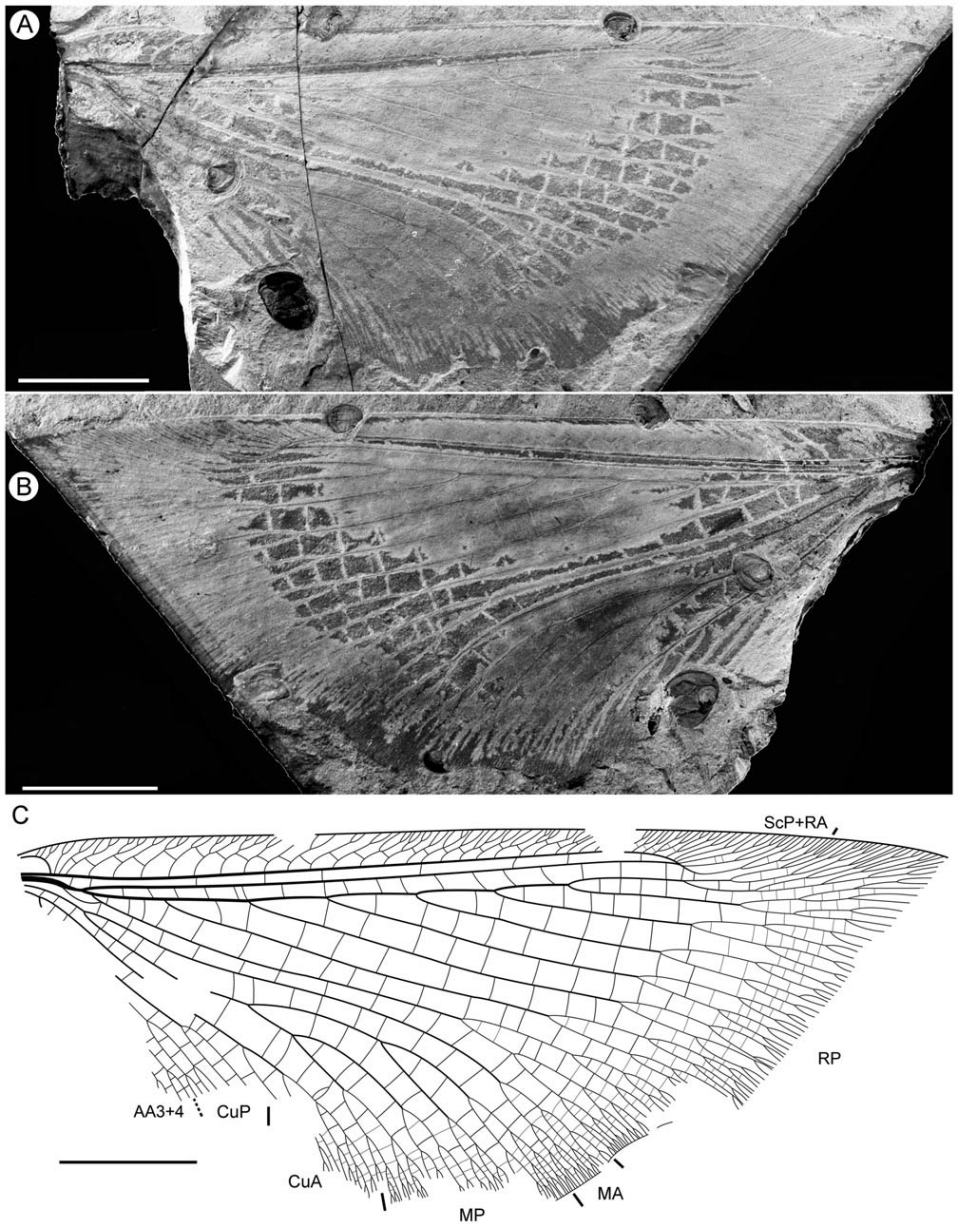
*Parakseneura directa* gen. et sp. nov., holotype CNU-NEU-NN2011016PC. A, photograph of part; B, photograph of counterpart; C, drawing of the hind wing venation. Scale bar 10 mm.

Hind wing 68 mm as preserved (estimated complete length about 70 mm), 27.5 mm wide. Costal margin straight, only slightly curved backward near apex; hind margin undulate. Trichosors detected along outer margin. Membrane hairs covered entire wing, but reduced on pale areas due to poor preservation. Costal space moderately broad; veinlets of ScP forked once or dichotomously; humeral veinlet recurrent, branched; crossveins form single series extending from wing base to fusion of ScP, RA. ScP+RA curved toward RP, relatively short, entering margin well before wing apex; ScP+RA, its 2–3 long branches dichotomously forked. Subcostal space narrow, with widely spaced crossveins. RA space relatively narrow, with quite numerous, irregularly spaced crossveins. RP with 8 branches, dichotomously branched distally; RP1 originated very near origin of RP. Basal r-m crossvein connects RP1 with R. M, R basally not fused, forked at nearly level of origin of RP1; MA slightly sinuate, dichotomously branched distally. MP deeply dichotomously branched. Cu forked near wing base. CuA nearly straight, pectinately branched, with 4 oblique branches. CuP deeply dichotomously branched. AA3+4 fragmentary preserved. Short crossveins posterior to stem of RP relatively closely spaced (between distal parts of branches), long crossveins more wide spaced. Color pattern unclear, probably darkish; veins, crossveins narrowly margined with pale.

#### Material Examined

Holotype CNU-NEU-NN2011016PC, deposited in CNUB; a nearly complete well-preserved hind wing.

#### Etymology

From Latin *directus*, straight, in reference the costal margin of the hind wing being straight (not excised or exserted).

#### Occurrence

Middle Jurassic, Bathonian/Callovian, Jiulongshan Formation; Daohugou Village, Shantou Township, Ningcheng County, Inner Mongolia, China.

#### Remarks

This hind wing may not be assigned to any species whose hind wings (or their costal margin) are known.

### Parakseneura sp. indet. A

#### Description (Fig. 16)

**Figure 16:**
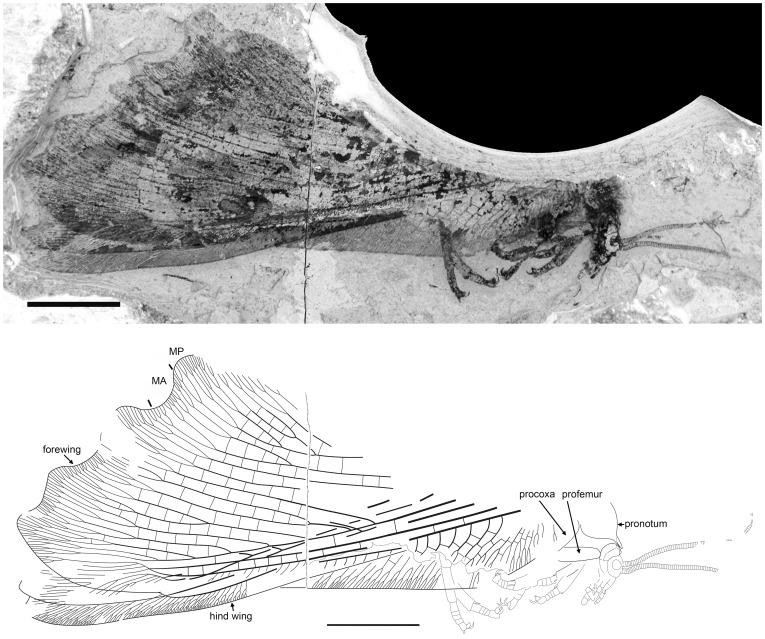
*Parakseneura* sp. indet. A, specimen CNU-NEU-NN2011020. A, photograph; B, drawing of the specimen as preserved. Scale bar 10 mm.

Head relatively small, with large eyes; ventrally with very long dense, fine hairs. Antennae appear filiform; very stout basally, becoming gradually more attenuate towards apex; probably much shorter than forewing. Maxillary palpi relatively short, thinner than labial palpi. Labial palpi relatively short, stout, probably three-segmented. Pronotum large, covered with dense, long hairs. Legs relatively short, stout. Foreleg: coxa poorly preserved, elongate, stout; femur broad, covered with relatively long, dense hairs; tibia twice narrower than femur, covered with dense hairs, with two distal short straight spurs; tarsus covered with dense, short hairs; five-segmented, with basitarsus, fifth segment longest; fifth segment distally with several long bristles; pretarsus with arolium and two big, strongly curved claws. Preserved parts of mid-leg, hind leg in general constructed similarly.

Forewing about 60 mm long. Costal space broad, strongly broadened proximally. Subcostal veinlets dichotomously forked, proximally connected by 1–3 crossveins. Subcostal space moderately broad, with rather closely spaced crossveins. ScP+RA quite strongly curved toward RP, relatively long, with three long branches. RP with 9 branches, of these two deeply forked. MA dichotomous, rather deeply forked. MP not completely preserved, heavily branched. Outer wing margin strongly undulate. Crossveins posterior to stem of RP rare, quite regularly spaced. Color pattern unclear, variegated with black, fuscous, pale areas.

Hind wing fragmentarily preserved. Costal margin slightly curved backward before fusion of ScP, RA. Subcostal veinlets dichotomously forked. Color pattern unclear.

#### Material Examined

CNU-NEU-NN2011020, deposited in CNUB; a well-preserved incomplete specimen (lateral aspect) with all wing overlapped.

#### Occurrence

Middle Jurassic, Bathonian/Callovian, Jiulongshan Formation; Daohugou Village, Shantou Township, Ningcheng County, Inner Mongolia, China.

#### Remarks

The venation and color patter are in general similar to those of *Parakseneura nigromacula* sp. nov., but diagnostic features are not distinct. Therefore, we treat it as indeterminate specimen.

### *Parakseneura* sp. indet. B

#### Description (Fig. 17)

**Figure 17:**
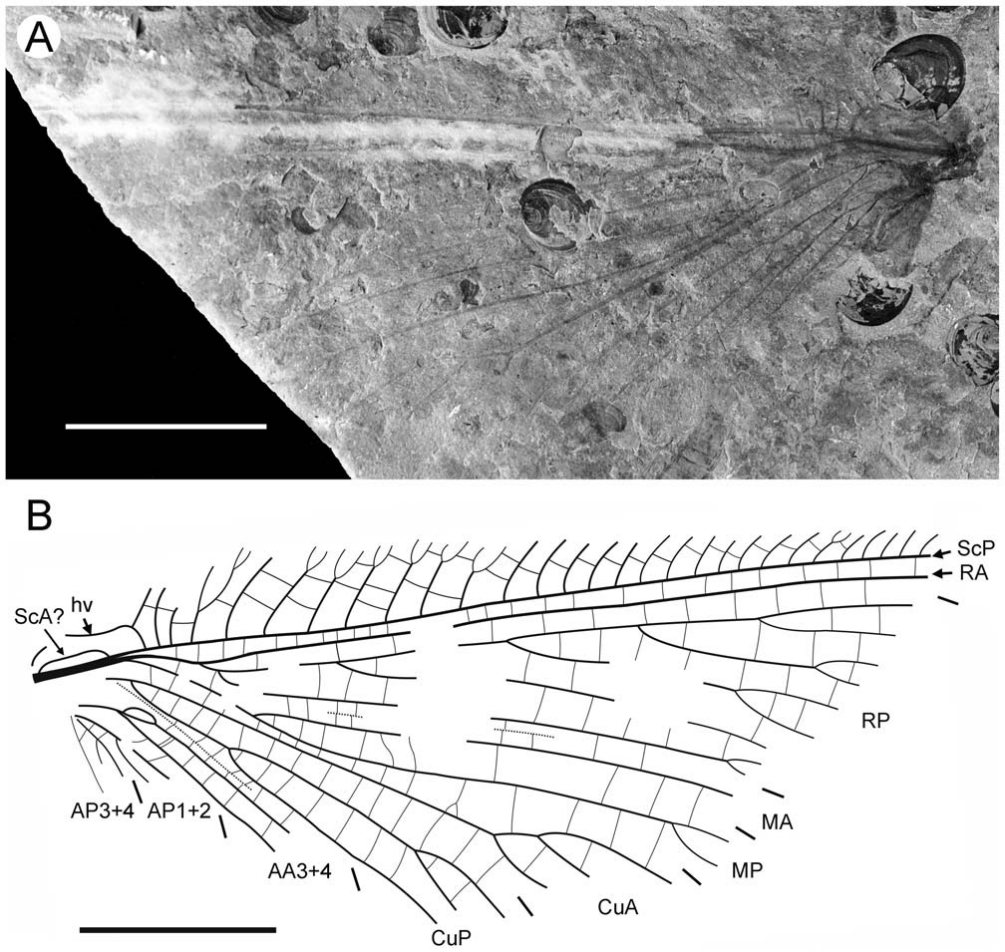
*Parakseneura* sp. indet. B, specimen CNU-NEU-NN2011012. A, photograph; B, drawing of the forewing venation. Scale bar 10 mm.

Forewing 47 mm long as preserved (estimated complete length about 65–75 mm), about 20 mm wide as preserved. Subcostal veinlets connected by one-three costal crossveins. Humeral veinlet strongly recurrent, branched. Sclerotized bulge margined with presumed ScA; another veinal structure anterior to it. Subcostal space with quite closely spaced crossveins. RA space slightly wider than subcostal space, with quite scarce crossveins. RP with 5 preserved branches, some deeply forked; RP1 originated near to origin of RP. M closely approaching R, forked distal to origin of RP1. MA running parallel to RP, probably with only terminal branching (not preserved). MP forked far distal to proximal-most branch of CuA. MP, MA slightly divergent. MP, CuA approach to each other for short length. Cu forked near wing base. CuA straight before branching, probably pectinately branched (two branches preserved). CuP deeply forked; anterior branch forked. Presumed AA1+2 present, short, fused with AA3+4 forming basal ‘loop’. AA3+4 deeply forked, approximately at level of Cu fork. AP1+2, AP3+4 deeply forked. Medial, claval folds present. Crossveins posterior to stem of RP irregularly spaced. Color pattern unclear, lacking dark spots.

#### Material Examined

CNU-NEU-NN2011012, deposited in CNUB; a proximal part of a quite poorly preserved forewing.

#### Occurrence

Middle Jurassic, Bathonian/Callovian, Jiulongshan Formation; Daohugou Village, Shantou Township, Ningcheng County, Inner Mongolia, China.

#### Remarks

This specimen is similar to *P*. *cavomaculata* sp. nov. by the absence of dark color pattern and the venation. It might be in principle assigned to this species. Unfortunately, two forewing pale spots characteristic of *P*. *cavomaculata* sp. nov. (see diagnosis of that species) are not detected due to incomplete preservation.

### *Parakseneura* sp. indet. C

#### Description (Fig. 18)

**Figure 18:**
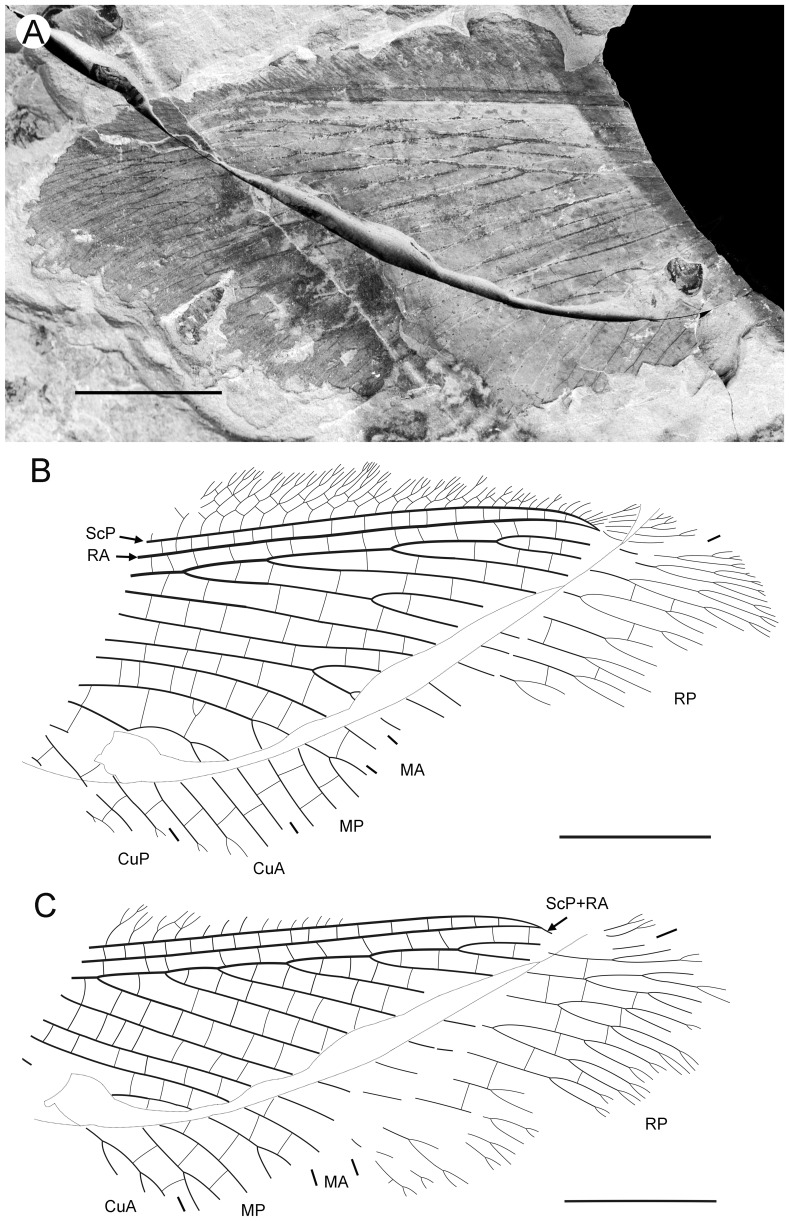
*Parakseneura* sp. indet. C, specimen CNU-NEU-NN2011028. A, photograph; B, drawing of the forewing venation; C, drawing of hind wing venation (both converted to the right). Scale bar 10 mm.

Forewing about 48 mm long as preserved (estimated complete length about 70 mm), about 25 mm wide as preserved. Subcostal veinlets dichotomously branched, connecting by one-two costal crossveins in distal part. ScP+RA relatively short, proximally bent toward RP, in general incurved, with one long veinlet. Subcostal, RA spaces nearly equal in width, with quite dense crossveins. RP with 7 branches before pterostigmal region. RA1 profusely branched. MA probably with only terminal branching (not preserved). MP distally parallel to MA; pectinately branched, with 3 preserved branches. CuA probably dichotomously branched (not completely preserved); proximal-most fork of CuA far proximal to proximal-most branch of MP. CuP fragmentarily preserved. Anal veins not preserved. Crossveins posterior to stem of RP scarce, irregular. Color pattern unclear, lacking dark spots.

Hind wing 47 mm long as preserved, about 21 mm wide as preserved. Subcostal, RA spaces nearly equal in width, with quite dense crossveins. RP with 9 branches before pterostigmal region. MA probably with only terminal branching (not preserved). Anterior branch of MP distally parallel to MA; MP profusely branched, not pectinate. CuA fragmentarily preserved. Crossveins posterior to stem of RP scarce, quite irregularly spaced between branches of RP. Color pattern unclear, lacking dark spots.

#### Material Examined

CNU-NEU-NN2011028, deposited in CNUB; a distal part of fore- and hind wings overlapped.

#### Occurrence

Middle Jurassic, Bathonian/Callovian, Jiulongshan Formation; Daohugou Village, Shantou Township, Ningcheng County, Inner Mongolia, China.

#### Remarks

This specimen is similar to *P*. *cavomaculata* sp. nov. by wings size, the absence of dark color pattern, and the venation. It might be assigned to this species, but two forewing pale spots characteristic of that species are not detected due to incomplete preservation.

### Parakseneura sp. indet. D

#### Description (Fig. 19)

**Figure 19:**
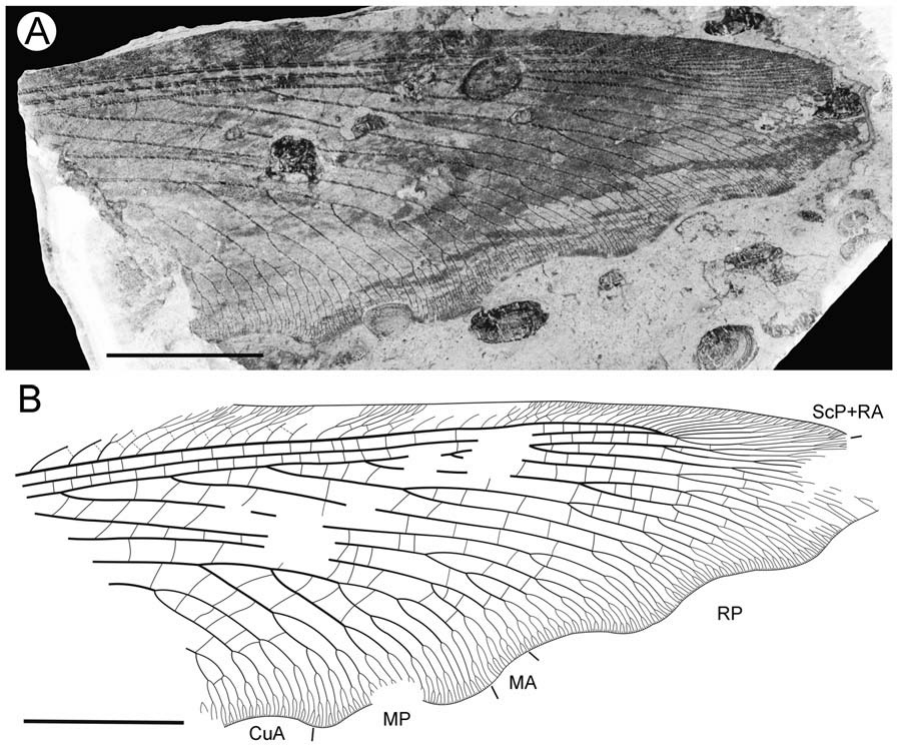
*Parakseneura* sp. indet. D, specimen CNU-NEU-NN2011025. A, photograph; B, drawing of the forewing venation. Scale bar 10 mm.

Forewing 53 mm long as preserved (actual probable length about 40–45 mm; estimated complete length about 60 mm), 20.5 mm wide as preserved; wing strongly narrowed towards apex probably due to post-sedimentation deformation of rock. Costal margin almost straight, only slightly gently curved backward in apical portion; outer, distal part of hind margins undulate. Trichosors prominent along hind margin, not detected along costal margin. Costal space dilated proximally. Subcostal veinlets dichotomously branched, connecting by poorly preserved costal crossveins. ScP, RP fused. ScP+RA slightly curved toward RP, with 5 long veinlets, enter margin well before wing apex. Subcostal, RA spaces equal in width, with regularly spaced, numerous crossveins. RP with 8 branches before pterostigmal region, majority of these deeply dichotomously branched. RP1, MA approach in middle of length. MA distally dichotomously branched. MP pectinate, with 4 dichotomously branched branches. CuA incomplete, probably dichotomous. Crossveins posterior to stem of RP relatively scarce, not forming gradate series. Color pattern most resembles that of type species, with distinct narrow undulate dark stripes near outer, hind margins.

#### Material Examined

Specimen CNU-NEU-NN2011025, deposited in CNUB; a well-preserved distal two thirds of a forewing.

#### Occurrence

Middle Jurassic, Bathonian/Callovian, Jiulongshan Formation; Daohugou Village, Shantou Township, Ningcheng County, Inner Mongolia, China.

#### Remarks

We consider this specimen to be distorted (lengthened) during the post-sedimentation deformation of rock. Similar distorted specimens belonging to other families occur very rarely in this locality (e.g., one specimen of Chrysopidae).

The forewing color pattern of this specimen is most similar (but not completely identical) to that of *P. undula* sp. nov. Their venation somewhat differs (e.g., MP is pectinate in *Parakseneura* sp. indet. D).

### Shuraboneura Khramov & Makarkin, gen. nov

urn:lsid:zoobank.org:act:AA813B1C-F008-44EC-93DB-F09F5A13D3BC

#### Type and Only Species

*Shuraboneura ovata* sp. nov.

#### Diagnosis

Forewing broad-oval (narrower in *Pseudorapisma*), with hind, outer margins smooth (undulate in *Parakseneura*); ScP, RA distally separate (fused in *Parakseneura*); intracubital space narrow, not broadened distally (wide, broadened distally in *Pseudorapisma*); branches of MP, CuA at some angle with hind margin (nearly parallel to hind margin in *Pseudorapisma*).

#### Etymology

From Shurab, a town in Kyrgyzstan and alternative name of the locality (“Shurab-3”), and Neuroptera. Gender feminine.

### Shuraboneura ovata Khramov & Makarkin, sp. nov

urn:lsid:zoobank.org:act:37A03E41-26CC-48B3-BAC2-27BEAEB5A342

#### Description (Fig. 20)

**Figure 20:**
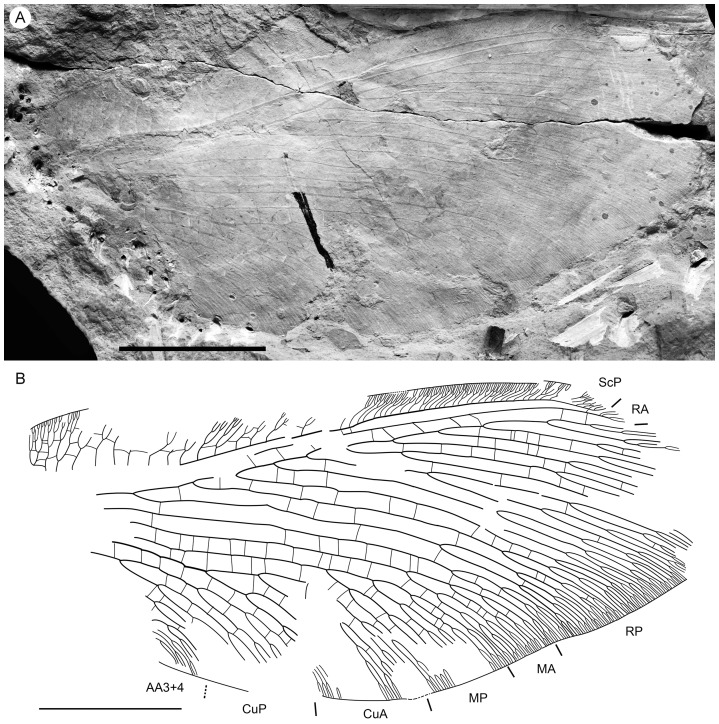
*Shuraboneura ovata* gen. et sp. nov., holotype PIN 2389/509. A, photograph; B, drawing of the forewing venation Scale bar 10 mm.

Forewing ovate, 44 mm long (as preserved; estimated complete length about 50 mm), 21.5 mm wide (as preserved; estimated complete width 22 mm). Membrane hairs not detected. Trichosors not distinct. Costal space proximally broadened, gradually narrowed towards apex. All subcostal veinlets dichotomously forked; in distal half very closely spaced. Humeral veinlet well developed, strongly recurrent. One to two costal crossveins between two subcostal veinlets in proximal half of wing. ScP, RA distally separated. Apical part of RA not curved backward, with at least four veinlets. Subcostal space narrow, with scarce crossveins. RA space relatively narrow, with rather irregularly spaced mainly oblique crossveins. RP with 12 branches, some deeply forked before marginal branching. Scarce irregularly spaced crossveins between branches of RP. Fork of M not preserved. MA not forked before marginal branching. MP deeply dichotomously branched. Fork of Cu not preserved. CuA branched well proximal to branching of MP; dichotomous or pectinate with three dichotomously branched branches (incompletely preserved). CuP constructed similarly to CuA: probably pectinate with three dichotomously forked oblique branches (incompletely preserved). AA3+4 incomplete, probably multi-branched. Crossveins in medial, cubital spaces irregular. Color pattern not preserved.

#### Material Examined

Holotype PIN 2389/509, deposited in PIN; a quite well preserved almost complete forewing.

#### Etymology

From the Latin *ovatus*, oval, ovate, in reference to oval shape of the forewing.

#### Occurrence

Early/Middle Jurassic, Sogul Formation; Say-Sagul locality, Osh Region, Kyrgyzstan.

### Shuraboneura sp. indet

#### Description (Fig. 21)

**Figure 21:**
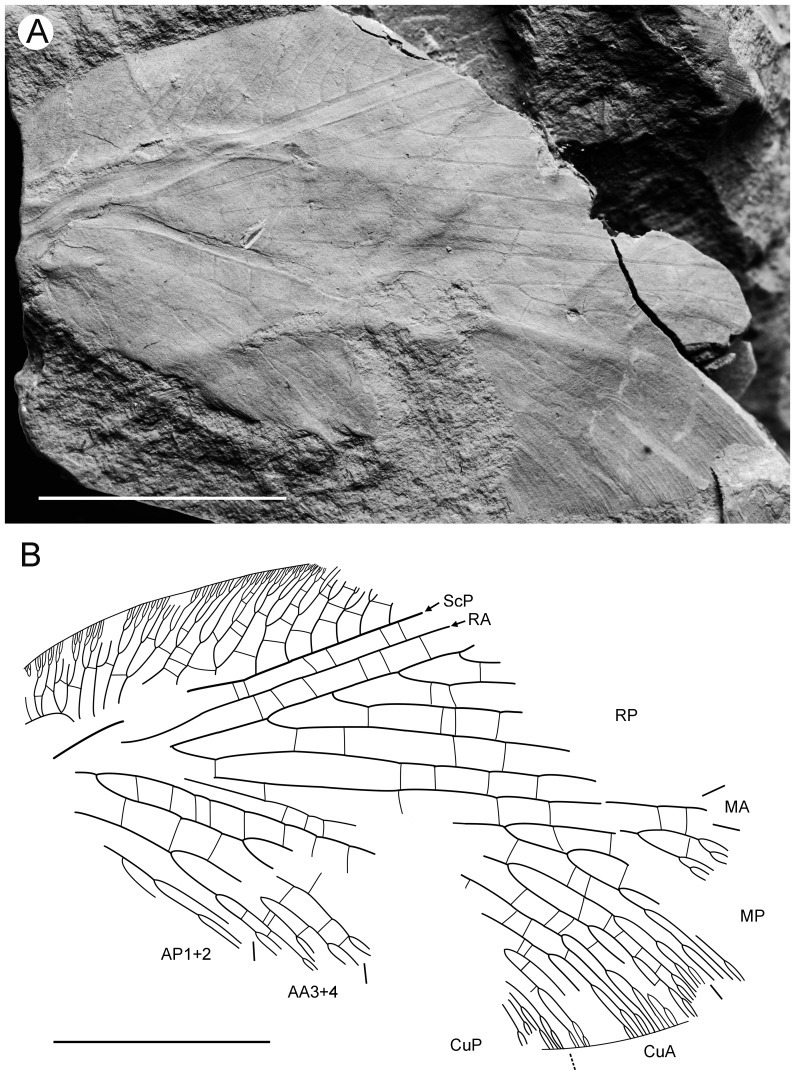
*Shuraboneura* sp. indet., specimen PIN 2345/334. A, photograph; B, drawing of the forewing venation. Scale bar 10 mm.

Forewing 27 mm long (as preserved; estimated complete length about 50 mm), 18.5 mm (as preserved; estimated complete width about 21 mm). Membrane hairs not detected. Trichosors not distinct. Costal space proximally broadened. All subcostal veinlets dichotomously forked, including branches of humeral veinlet. Humeral veinlet well-developed, strongly recurrent. One to three costal crossveins between two subcostal veinlets in proximal half of wing. Subcostal space rather narrow, with scarce crossveins. RA space relatively narrow, with irregularly spaced perpendicular to RA crossveins. RP1 originated near to origin of RP. Few irregularly spaced crossveins between branches of RP. M forked somewhat distal to origin of RP1. MA straight proximally. MP probably deeply dichotomously branched. Cu dividing into CuA and CuP very near to wing base. CuA probably dichotomously branched (incompletely preserved). CuP probably pectinate (incompletely preserved). AA3+4 dichotomously branched with proximal-most fork located well distal to dividing of Cu, nearly at same level as fork of M. AP1+2 fragmentarily preserved. Crossveins in medial, cubital spaces irregular. Color pattern not preserved.

#### Material Examined

Specimen PIN 2345/334, deposited in PIN; a basal part of forewing.

#### Occurrence

Early/Middle Jurassic, Sogul Formation; Say-Sagul locality, Osh Region, Kyrgyzstan.

#### Remarks

The venation of this specimen is in general similar to that of *Shuraboneura ovata* sp. nov., but its fragmentary nature does not allow sure assignment.

The following fragmentary wings of Parakseneuridae are known from that locality: PIN 2389/458, PIN 2389/493 (forewings); PIN 2032/499, PIN 2389/443 (probably hind wings); PIN 2061/115; PIN 2032/500; PIN 2389/444. These fragments are at present not possibly to attribute to species.

### Pseudorapisma Yang, Makarkin & Ren, gen. nov

urn:lsid:zoobank.org:act:814B1546-7E0E-46E6-9C04-0070E202290A

#### Type Species

*Pseudorapisma jurassicum* sp. nov.

#### Diagnosis

Forewing elongate (broadly-ovate in *Parakseneura*, *Shuraboneura*), about 50–70 mm long, with large humeral plate; ScP, RA apically separate (fused in *Parakseneura*); basal anal ‘loop’ formed by fusion of presumed AA1+2 and AA3+4 absent (present in *Parakseneura*). In hind wing, ScP, RA apically separate (fused in *Parakseneura*); CuA pectinately branched well proximal to branching of MP (distal in *Parakseneura*); basal sinuate crossvein between R and M systems absent (present in *Parakseneura*).

#### Occurrence

Middle Jurassic (Jiulongshan Formation) of Daohugou (Inner Mongolia, China).

#### Species Included

Three species: *Pseudorapisma jurassicum* sp. nov., *P. maculatum* sp. nov., *P. angustipenne* sp. nov.

#### Etymology

From the Greek *pseudos*, false, and *Rapisma* (a genus-group name), in reference to superficial resemblance in the venation to *Rapisma* McLachlan. Gender neuter.

### Pseudorapisma jurassicum Yang, Makarkin & Ren, sp. nov

urn:lsid:zoobank.org:act:764D778F-242C-4A8A-BF6E-3249C7A0ABD1

#### Diagnosis

Hind wing differs from that of *P. maculatum* sp. nov. by straight convex costal margin (convex in *P. maculatum* sp. nov.), and the absence of several small pale spots in the cubital space (present in *P. maculatum* sp. nov.).

#### Description

**Figure 22:**
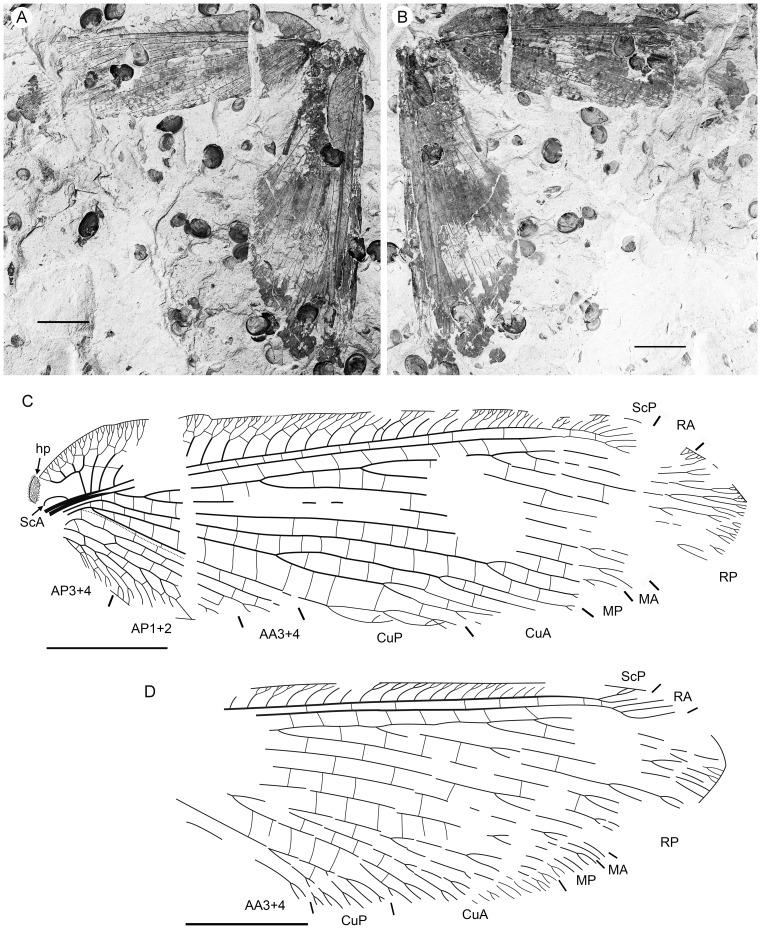
*Pseudorapisma jurassicum* gen. et sp. nov., holotype CNU-NEU-NN2011033PC. A, part; B, counterpart; drawing of the right forewing (C) and left hind wing (D) venation. Scale bar 10 mm.

*Holotype CNU-NEU-NN2011033P* (Figs. 22A–D). Mesothorax, metathorax, abdomen incomplete, very poorly preserved.

Right forewing about 58 mm long (based on reconstruction), about 18 mm wide as preserved (estimated complete width 19–20 mm). Humeral plate large, elongate, covered with dense quite long hairs. Costa covered with very short hairs. Trichosors not detected along costal margin, indistinct near apex (other margins not preserved). Costal space moderately broad, markedly dilated basally; all subcostal veinlets forked (dichotomously in proximal half, mainly once forked in distal half), connected by one-two crossveins proximally; humeral veinlet strongly recurrent, branched. Presumed ScA short, arched; ScP entering wing margin well before apex. Subcostal space relatively narrow, slightly dilated towards apex; crossveins irregularly spaced. RA distally slightly curved backward, with two long veinlets, entering wing margin well before apex. RA space much wider than subcostal space, with regularly spaced oblique crossveins. RP with 6 branches before pterostigmal region; RP1, RP2 slightly arched, proximally running almost parallel to costal margin. M not fused with R basally, forked well distal to origin of RP1 (fork not preserved). MA running parallel to RP1, branched near margin, probably dichotomously. MP dichotomously branched (alternatively, with one long branch originated much basad branching of MA). Cu dividing into CuA, CuP close to wing base. Cubital space broad; CuA, CuP divergent. CuA dichotomously branched, with primary fork located at approximately middle of length (alternatively, with one long forked branch). CuP nearly straight before branching, probably pectinate (with 2–3 branches); proximal-most branch located well distal to that of CuA. AA3+4 long, forked relatively far from wing base; both branches parallel to each other for long distance; distal branching not preserved. AP1+2 basally forked into two branches; anterior trace of anterior branch nearly straight, pectinately branched, with forked branches; posterior branch in general dichotomously branched. AP3+4 basally forked; anterior branch dichotomously branched; posterior branch probably once forked. Crossveins posterior to RP scarce, widely spaced, not forming gradate series. Membrane hairs preserved only in basal part, but much shorter and not so dense than in CNU-NEU-NN2011027PC, in other parts unclear, maybe very short or not preserved. Color pattern not distinct: proximal part darkish, distal 3/4 variegated with darkish and pale area, especially in cubital space.

Hind wings: left hind wing approximately 53 mm long (basal part poorly preserved), about 18.5 mm wide as preserved (estimated complete width about 19–20 mm). Trichosors not detected as margins poorly preserved. Costal space relatively narrow. Preserved subcostal veinlets once or twice forked, rather widely spaced; their distal parts strongly curved toward wing apex; no costal crossveins detected. ScP entering wing margin well before apex. Subcostal space moderately narrow, with space relatively widely spaced crossveins. R1 distally bent backward, with two long veinlets, terminating on costal margin well before apex. RP with 6 very oblique branches. RA narrow. MA, MP running almost parallel to each other and to RP1 for most length, both few branched distally. Anterior trace of CuA almost straight, with four very oblique pectinate branches, each dichotomously branched. CuP with 3 very oblique pectinate branches, proximal-most branch located well distally than that of CuA. Cubital space broad. Only incomplete anterior branch of AA3+4 preserved. Crossveins posterior to RA widely, rather regularly spaced, not forming gradate series. Membrane microtrichia not preserved. Color pattern similar to that of next specimen.

**Figure 23:**
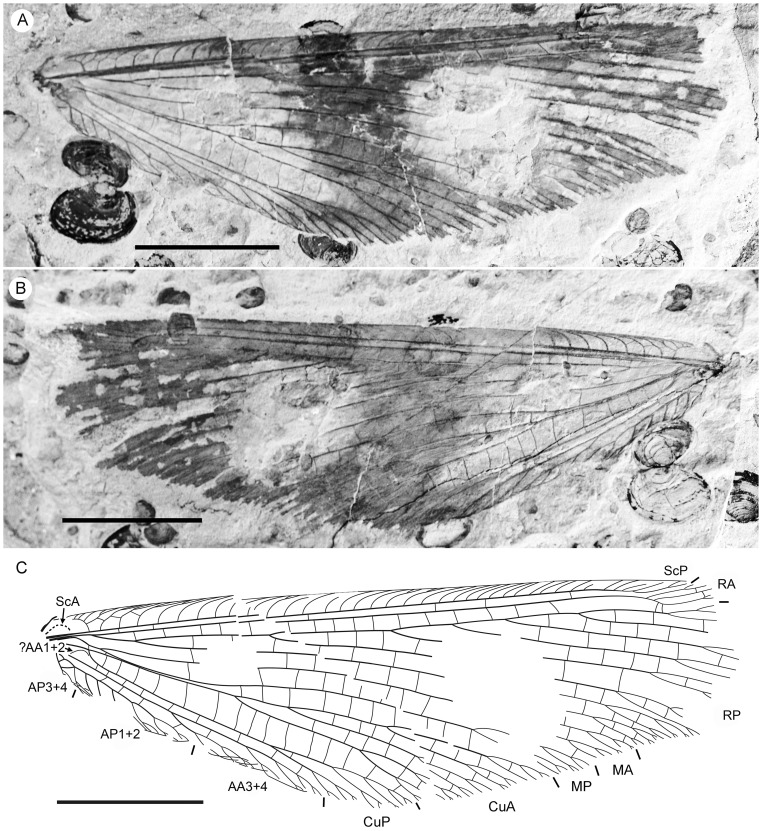
*Pseudorapisma jurassicum* gen. et sp. nov., paratype CNU-NEU-NN2011006PC. A, photograph of part; B, photograph of counterpart; C, drawing of the hind wing venation. Scale bar 10 mm.

*Paratype CNU-NEU-NN2011006PC* (Figs. 23A–C). Hind wing elongate, with quite well developed tornus; 48 mm as preserved (estimated complete length about 51 mm), about 15 mm wide. Fuscous areas of wing membrane covers with quite long hairs (especially well visible in apical part) not detected on pale areas. Trichosors not detected along costal margin (other margins not preserved). Humeral plate large covered with many fine hairs; frenulum bristles not detected. Costal space relatively narrow. Subcostal veinlets simple or once forked, rather widely spaced; their distal parts strongly curved toward wing apex (running almost parallel to wing margin), particularly in proximal wing portion; basal veinlets connected by 3 crossvein forming short series. Humeral veinlet recurrent, branched. Presumed ScA hardly visible, arched. ScP entering wing margin well before apex. Subcostal space moderately narrow, with widely spaced crossveins. R1 distally bent backward, with two long veinlets, terminating on costal margin. RP originated near wing margin at very acute angle; with 7 very oblique branches; three proximal-most branches spaced more widely than others. RA space relatively narrow, slightly broadened towards wing apex. Basal sinuate r-m crossvein between R and M systems not detected, although this wing region well preserved. M, R probably closely approach basally; M forked well proximal to origin of proximal-most branch of RP. MA, MP running almost parallel to each other for most length, sinuously curved in proximal portion, both few branched distally. Proximal parts of MP and CuA closely approach for some length. Cu dividing into CuA and CuP relatively far to wing apex. Anterior trace of CuA almost straight, with very oblique pectinate branches, of these three proximal-most long, dichotomously branched. Anterior trace of CuP almost straight, with 4 very oblique pectinate branches, shorter than those of CuA. Cubital space very broad. Presumed AA1+2 short, arched terminating near CuP. AA3+4 long, straight, pectinately branched, running parallel to CuP; proximal-most branch pectinate. AP1+2 parallel to AA3+4, pectinately branched (approximately with 6 branches). AP3+4 short, once forked. Crossveins posterior to RA widely, rather regularly spaced, not forming gradate series. Color pattern: proximal third of wing pale; distal two-thirds dark with large rounded pale spot.

**Figure 24:**
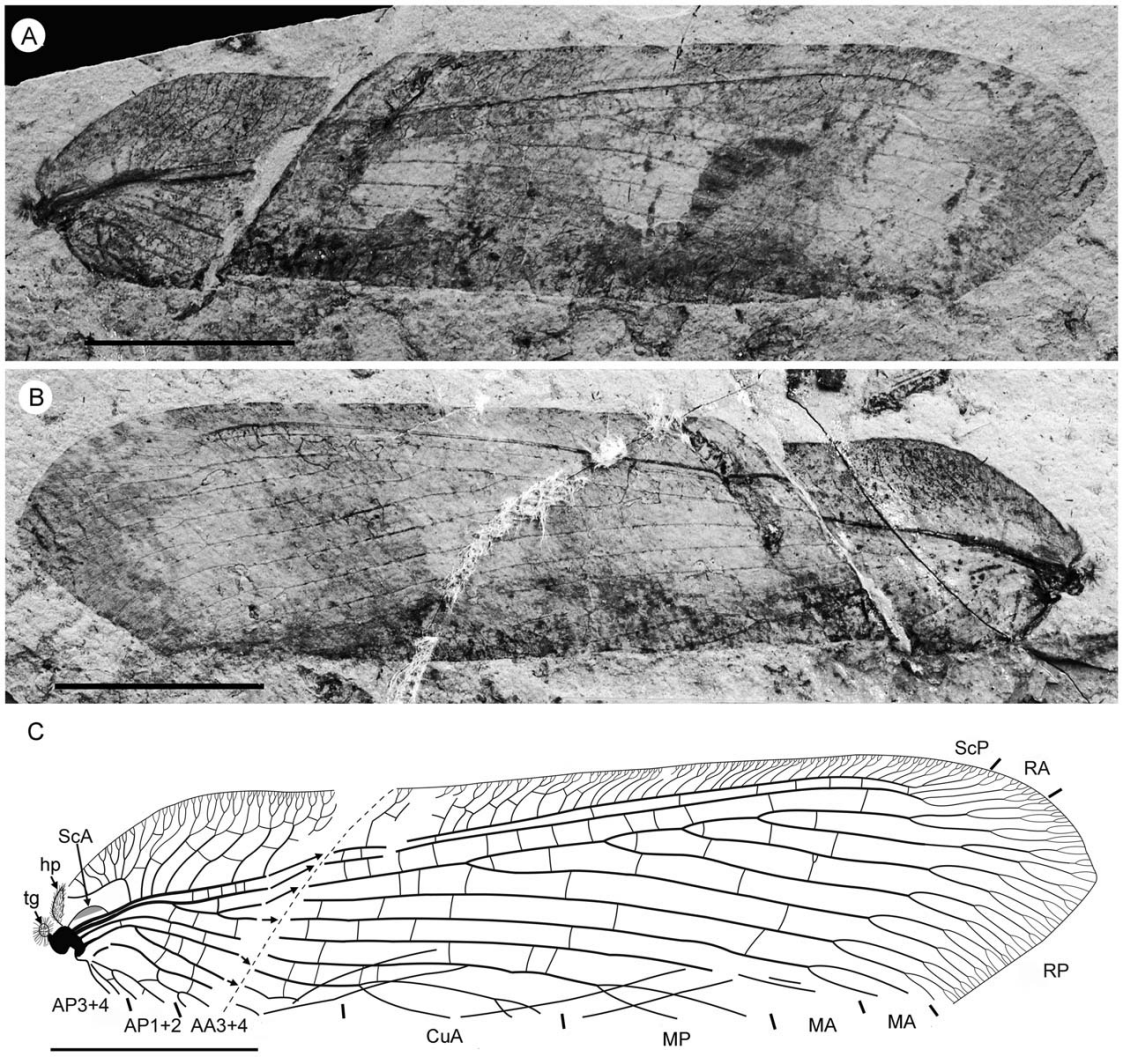
*Pseudorapisma jurassicum* gen. et sp. nov., specimen CNU-NEU-NN2011027PC. A, part; B, counterpart; C, drawing of the forewing venation. Scale bar 10 mm.

*Specimen CNU-NEU-NN2011027PC* (Figs. 24A–C). Forewing about 51 mm long (based on reconstruction), 12 mm wide as preserved (complete width impossible to estimate). Tegula rounded, with dense long hairs. Humeral plate large, elongate, covered with quite long hairs. In humeral area, heavily sclerotized bulge, and presumed ScA anterior to it clearly visible. Wing membrane covered with hairs, especially long in basal portion of costal space. Trichosors preserved along entire margin, not distinct. Costal space proximally dilated, gradually narrowed toward apex. All subcostal veinlets dichotomously forked. Humeral veinlet well developed, strongly recurrent, with 6 branches (four forked, two simple). One to two costal crossveins between two subcostal veinlets in proximal half of wing. ScP, RA widely separated distally; both enter margin well before wing apex. Apical part of RA curved toward RP, with two long dichotomously forked veinlets. Subcostal space narrow, with scarce crossveins. RA space relatively narrow, slightly dilated toward wing apex, with rather closely spaced crossveins. RP originated quite far from wing base, with 7 branches, each not forked before marginal branching. Few irregularly spaced crossveins between branches of RP. RP1, RP2 proximally parallel to costal margin, then running at acute angle to it, and parallel to other branches of RP. M not fused basally with R; its fork not preserved, located distal to origin of RA1. MA, MP long, slightly divergent toward apex; MP forked before marginal branching. Cu dividing into CuA and CuP near wing base. Primary fork of CuA forked approximately at proximal 1/3 of wing length. CuP poorly preserved. Basal configurations of AA3+4, AP1+2, AP3+4 in general as in the holotype. Crossveins posterior to RP rare, irregularly spaced. Color pattern indistinct, brownish variegated with pale, and three large pale regions.

**Figure 25:**
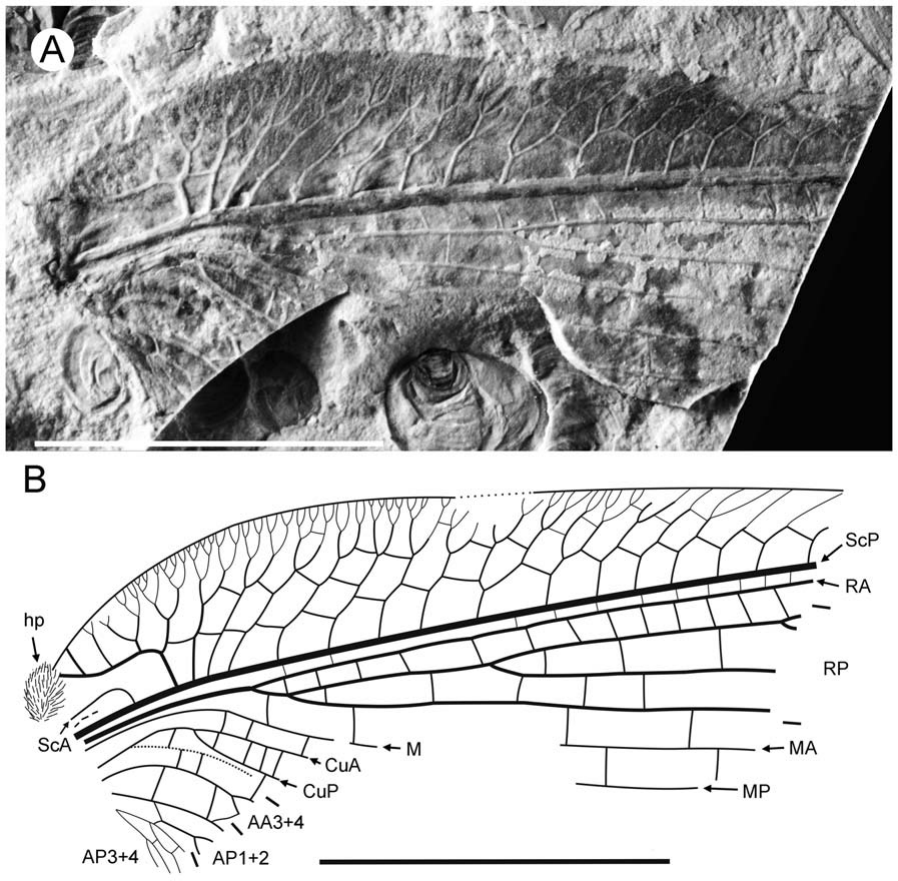
*Pseudorapisma jurassicum* gen. et sp. nov., specimen CNU-NEU-NN2011013. A, photograph; B, drawing of the forewing venation. Scale bar 10 mm.

*Specimen CNU-NEU-NN2011013* (Figs. 25A, B). Forewing 23.5 mm long as preserved (estimated complete length about 50 mm). Humeral plate large, appearing oval, covered with quite long hairs. Trichosors along preserved proximal part of costal margin not detected. Membrane hairs not detected (except some long hairs near humeral plate). Costal space broad, dilated basally. Subcostal veinlets dichotomously branched proximally. Humeral veinlet well developed, strongly recurrent, branched. One to three costal crossveins between subcostal veinlets. ScP stout. Subcostal space very narrow, with scarce fine crossveins. RA space narrow, slightly dilated toward wing middle; with rather regularly spaced numerous crossveins. RP originated quite far from wing base. Three proximal-most branches of RP widely spaced, of them two proximal-most branches parallel to costal margin. M not fused basally with R, forked well distal to origin of RP1 (fork not preserved). MA, MP (in proximal portion of wing) parallel to each other and costal margin. Cu dividing into CuA, CuP near wing base. Claval fold between CuP, AA3+4 distinct. AA3+4 forked well distal to fork of Cu. AP1+2 forked near wing base; posterior branch forked nearly at level of AA3+4 fork. AP3+4 forked at wing base. Crossveins posterior to stem of RP scarce, irregularly spaced. Color pattern indistinct, in general brownish variegated with pale.

#### Material Examined

Holotype CNU-NEU-NN2011033PC, a quite poorly preserved incomplete specimen in ventral aspect; right forewing nearly complete, rather well-preserved; right hind wing lacks; left forewing (only few portions visible) hidden under poorly preserved left hind wing. Paratype CNU-NEU-NN2011006PC, a nearly complete well-preserved hind wing. Specimens not included in the type series: CNU-NEU-NN2011027PC, an almost complete, quite well preserved forewing with the hind margin folded up; CNU-NEU-NN2011013, a well-preserved basal portion of a forewing. All deposited in CNUB.

#### Etymology

After the Jurassic period.

#### Occurrence

Middle Jurassic, Bathonian/Callovian, Jiulongshan Formation; Daohugou Village, Shantou Township, Ningcheng County, Inner Mongolia, China.

#### Remarks

The species identity of the holotype and paratype is undoubted, verified by very similar preserved venation and coloration of their hind wings. Other two forewings are assigned to this species preliminary, because of slightly different color pattern (possibly, however, due to quite poor preservation).

### Pseudorapisma maculatum Yang, Makarkin & Ren, sp. nov

urn:lsid:zoobank.org:act:047903FC-D117-4930-A996-FDF780B476FB

#### Diagnosis

Hind wing differs from that of *P. jurassicum* sp. nov. by convex costal margin (straight in *P. jurassicum* sp. nov.), and the presence of several small pale spots in the cubital space (absent in *P. jurassicum* sp. nov.).

#### Description (Fig. 26)

**Figure 26:**
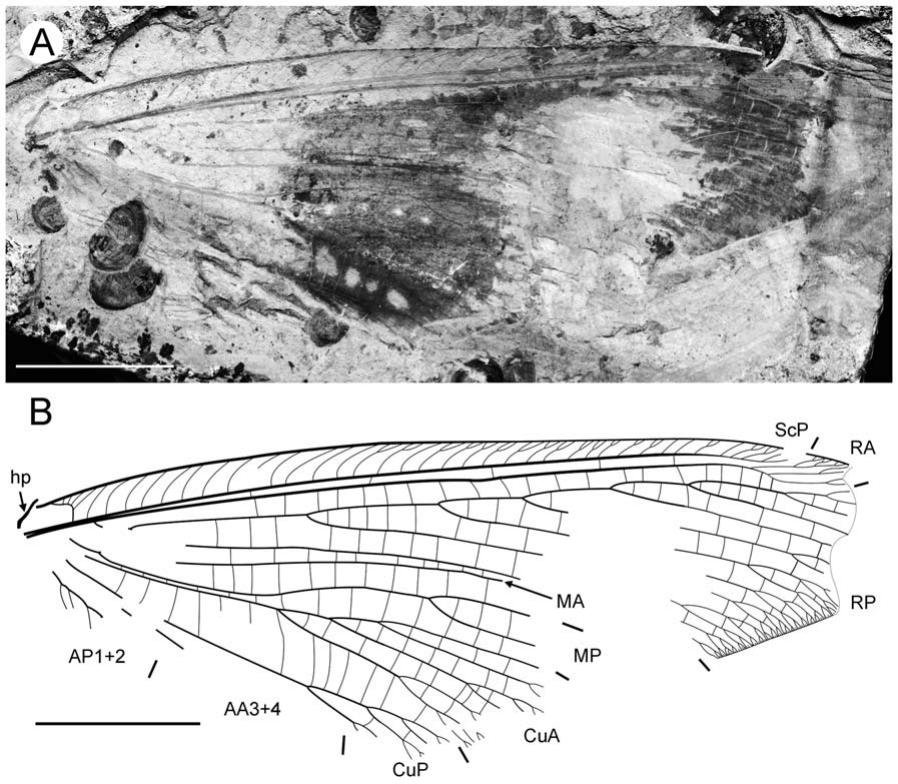
*Pseudorapisma maculatum* gen. et sp. nov., holotype CNU-NEU-NN2011018. A, photograph; B, drawing of the hind wing venation. Scale bar 10 mm.

Hind wing with convex costal margin; 51 mm as preserved (estimated complete length about 55 mm), about 19.5 mm wide (estimated complete length 20 mm). Trichosors distinct along preserved outer margin; not detected along costal margin. Membrane hairs not detected. Humeral plate large covered with many short hairs. Costal space relatively broad. Subcostal veinlets oblique, simple proximally, once forked distally; costal crossveins not detected. Humeral veinlet recurrent, branched. Presumed ScA not detected. ScP entering wing margin well before apex. Subcostal space moderately narrow, appears dilated toward apex, with widely spaced crossveins. RA terminating on costal margin well before apex, distally bent backward, with two long veinlets. RP originated near wing base, with 7 very oblique branches; RP1 originated close to origin of RP. RA space relatively narrow. Basal sinuate r-m crossvein between R and M systems not detected (or not preserved). M forked proximal to origin of proximal-most branch of RP. MA, MP running almost parallel to each other for most length, sinuously curved in proximal portion; MP deeply forked. Proximal parts of MP, CuA closely approach for some length; between them several very short crossveins. Cu dividing into CuA and CuP close to wing apex. Anterior trace of CuA almost straight proximally, with 3 long, very oblique pectinate branches, each dichotomously branched. Anterior trace of CuP almost straight, with 3 oblique pectinate branches, shorted than those of CuA. Cubital space very broad. AA3+4 fragmentarily preserved. AP1+2 pectinately branched (not completely preserved). AP3+4 not preserved. Crossveins posterior to RA widely, rather regularly spaced, not forming gradate series. Color pattern: proximal third of wing, half of costal and subcostal spaces pale; distal two thirds dark with pale spots: one larger in radial space, four small in cubital space.

#### Material Examined

Holotype CNU-NEU-NN2011018, deposited in CNUB; a nearly complete well-preserved hind wing.

#### Etymology

From the Latin *maculatus*, spotted, in reference to pale spots in the cubital space of the hind wing.

#### Occurrence

Middle Jurassic, Bathonian/Callovian, Jiulongshan Formation; Daohugou Village, Shantou Township, Ningcheng County, Inner Mongolia, China.

#### Remarks

The attribution of this hind wing to the genus *Pseudorapisma* gen. nov. is undoubted as its venation is very similar to that of the type species.

### Pseudorapisma angustipenne Yang, Makarkin & Ren, sp. nov

urn:lsid:zoobank.org:act:E3CABB3A-7A3B-45F8-8E26-A25C4236BFF6

#### Diagnosis (Fig. 27)

**Figure 27:**
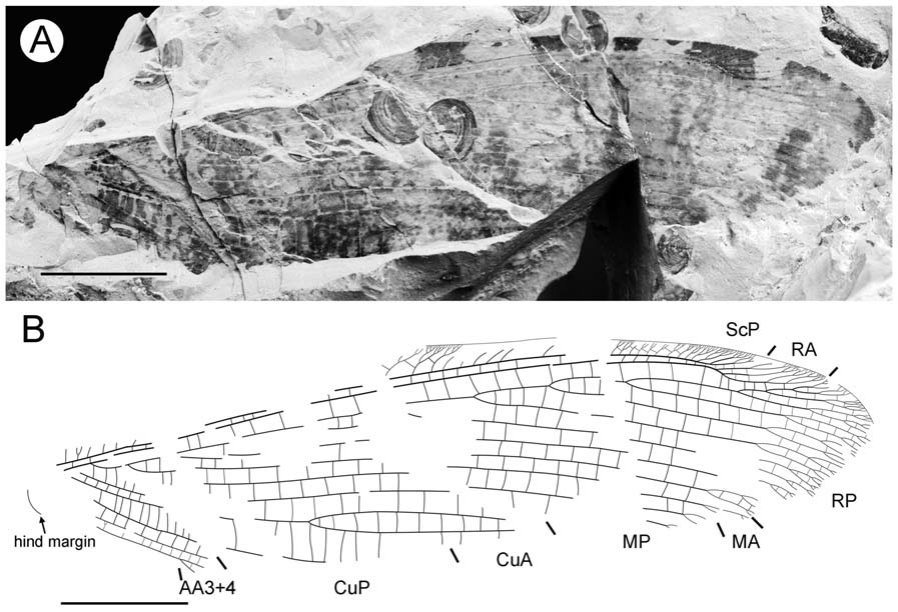
*Pseudorapisma angustipenne* gen. et sp. nov., holotype CNU-NEU-NN2011014. A, photograph; B, drawing of the forewing venation. Scale bar 10 mm.

Forewing differs from that of *P. jurassicum* by denser crossveins, in particular between terminal branches of RP, and sinuous CuA (in general straight in *P. jurassicum*).

#### Description

Forewing about 67 mm long, 16 mm wide as preserved (complete width probably about 20 mm). Trichosors prominent apically, not detected along costal margin. Membrane hairs relatively long apically and in costal space; shorter in other wing regions. All preserved subcostal veinlets dichotomously branched; between then (except for several distal-most) one costal crossvein. ScP entering wing margin well before apex. Subcostal space narrow, with quite closely spaced crossveins. RA terminating on costal margin well before apex, distally bent backward with two long veinlets. RA space with rather regularly spaced numerous crossveins. RP with 7 widely spaced oblique branches before pterostigmal region; two proximal-most branches proximally parallel to costal margin, then arched. M forked distal to origin of RP1 (fork not preserved); MA, MP parallel to each other, RP1 (i.e., in general arched); MA only with terminal branching (in area of end twigging); MP branched somewhat proximally than MA (not preserved). Cu dividing into CuA, CuP near wing base (fork not preserved); CuA sinuous, in general parallel to M, with only deep fork preserved. CuP, CuA diverge; cubital space broad. Two branches of AA3+4 parallel in middle of length. AP1+2, AP3+4 not preserved. Crossveins posterior to stem of RP relatively dense, irregular, occur in particular between terminal branches of RP. Color pattern marmoraceous, i.e., with alternating pale and brownish areas.

#### Material Examined

Holotype CNU-NEU-NN2011014, deposited in CNUB; an incomplete forewing.

#### Occurrence

Middle Jurassic, Bathonian/Callovian, Jiulongshan Formation; Daohugou Village, Shantou Township, Ningcheng County, Inner Mongolia, China.

#### Etymology

From the Latin *angustus*, narrow, and *penna*, wing, in reference to narrow forewing.

#### Remarks

This species is tentatively assigned to *Pseudorapisma* gen. nov. due to incompleteness of the forewing of the only known specimen.

## Comparative Qualitative Analysis of Characters of Parakseneuridae

### Body Characters

**Figure 28:**
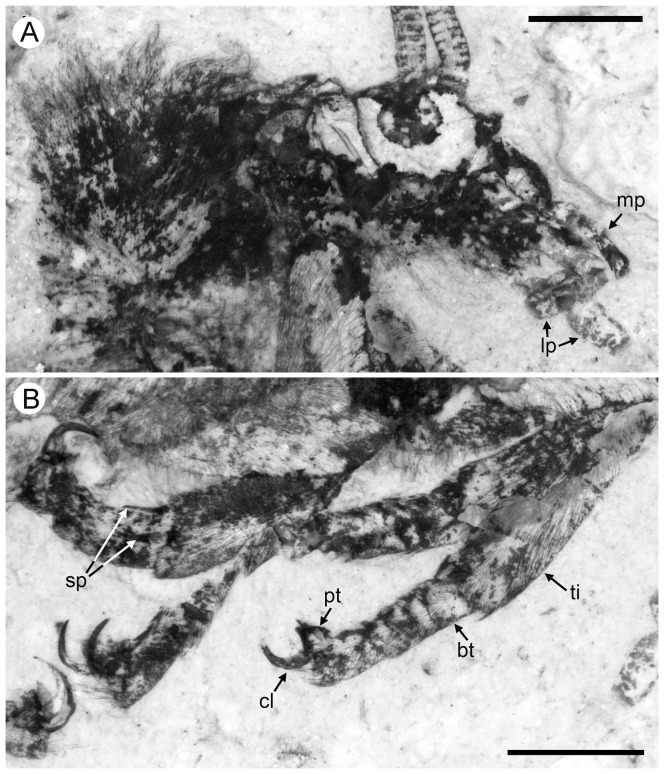
*Parakseneura* sp. indet. A., specimen CNU-NEU-NN2011020. A, head and pronotum; B, tibiae and tarsi of fore- and mid-legs. **bt**, basitarsus; **cl**, claw; **lp**, labial palps; **mp**, maxillary palpus; **pt**, pretarsus; **sp**, spurs; **ti**, foreleg tibia. Scale bar 2 mm.

The body (head, prothorax and legs) is partially preserved only in one specimen, i.e., *Parakseneura* sp. indet. A. (Figs. 16, 28). An incomplete, damaged and/or poorly preserved thorax is found in *Parakseneura albadelta* gen. et sp. nov., *P. cavomaculata* gen. et sp. nov., and *Pseudorapisma jurassicum* gen. et sp. nov., but no details are visible (Figs. 9B, 11, 22A, B). The legs are also partially preserved in *Parakseneura undula* gen. et sp. nov. and *P. curvivenis* gen. et sp. nov. (Figs. 3D–E, 5). The incomplete abdomen is preserved in two specimens (*Pseudorapisma jurassicum* gen. et sp. nov. and *Parakseneura undula* gen. et sp. nov.) but no details are visible.

Of the extant Neuroptera, the body of Parakseneuridae is most similar to that of some Australian Ithonidae (*Ithone* spp.) in its dense vestiture, the morphology of legs, palpus, and prothorax. Of the fossil families, the body of Parakseneuridae most resembles that of Kalligrammatidae, except for the structure of palpi. However, the body of some important fossil families is entirely unknown (e.g., Brongniartiellidae, Osmylopsychopidae) or fragmentary and poorly known (e.g., Prohemerobiidae, Panfiloviidae), so this comparison is incomplete.

#### Antennae

The stout, relatively short filiform antennae of this family most resemble those of Kalligrammatidae (Figure 1 in [70]; Figure 8 in [71]). The short antennae are also characteristic of many Ithonidae, some Mesochrysopidae, all Myrmeleontidae, Psychopsidae and Mantispidae [72].

#### Palpi

The labial palpi are very stout compared with those of most extant families, but not prolonged. Of extant Neuroptera, this morphology is more or less similar to that of the Australian Ithonidae (see Figure 6 in [73]). The structure of palpi in many fossil families is unknown.

#### Spurs

Two terminal spurs on each tibia are probably plesiomorphic condition in the order as it is found in such generalized families as Ithonidae, Nevrorthidae, Psychopsidae, and Osmylidae

### Wing Characters

#### Wing shape

The undulate forewing margin of *Parakseneura* is most similar to that of *Undulopsychopsis* Peng *et al*. (Psychopsidae) (Figures 1–3 in [74]. This condition occurs scarcely in Neuroptera. Of the extant taxa, this is found in some Hawaiian species of the hemerobiid genus *Micromus* Rambur [75]. This is also characteristic of some genera of two extinct families: a new undescribed family (e.g., see the Late Cretaceous *Palaeogetes ponomarenkoi* Makarkin, 1990, and an Early Cretaceous species from the Yixian Formation: Figure 6 in [76]; Figure 5B in [6]) and Saucrosmylidae from Daohugou [9], [77]. Most probably, this condition is an autapomorphy at various taxonomic levels and has evolved independently in various families multiple times.

The proximal half of the hind wings in Neuroptera is usually narrower than the distal half, but the hind wings of the type genus of Parakseneuridae are distinctly wider in proximal half than in distal. Similar wing shape is found in the extant Australian Ithonidae and Corydalidae. In all Kalligrammatidae and in the type genus of Brongniartiellidae, the hind wings are considerably wider than forewings, but this dilation falls mainly on their middle and/or distal half.

#### Membrane trichiation

**Figure 29:**
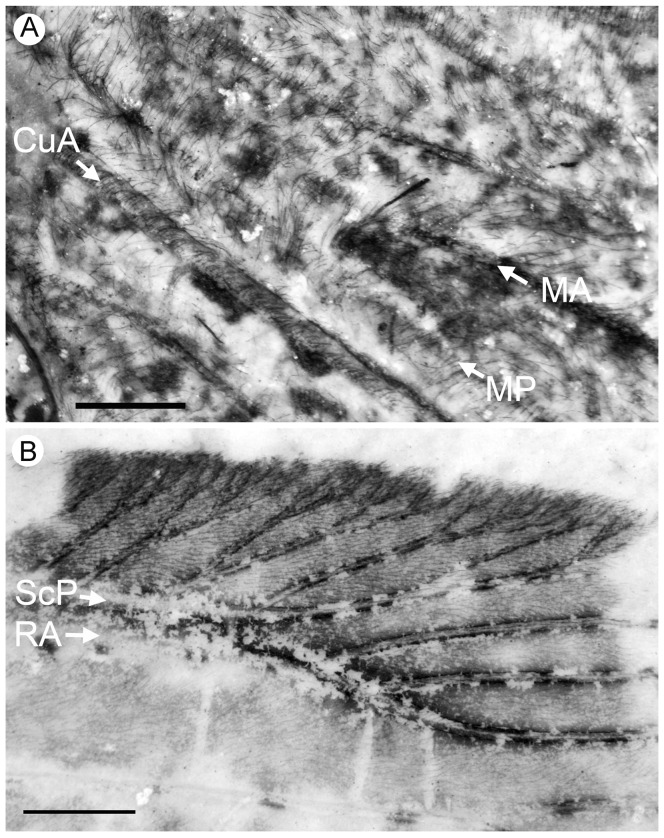
Long hairs on wing membrane of Parakseneuridae. A, middle portion of the forewing of *Parakseneura nigromacula* gen. et sp. nov., CNU-NEU-NN2011009; B, apical portion of the forewing of *Pseudorapisma angustipenne* gen. et sp. nov., CNU-NEU-NN2011014 (both wetted with ethanol). Scale bar 1 mm.

Two types of setae may occur on the wing membrane, sensilla (known also as ‘macrotrichia’) and spinules (known also as ‘microtrichia’) (terminology as in [78]). The latter are minute, usually not visible in the fossil, but are present in many extant taxa (see Table S1). Dense long setae on the wing membrane are not found in the extant Neuroptera, except for Ascalaphidae (some Haplogleniinae) where very scarce sensilla are present on both dorsal and ventral surfaces of both wings [79]. Both quite scarce, long sensilla and dense minute spinules occur on the forewing membrane of Sialidae (Figures 1, 6 in [80]). The long dense hairs are only found on the wing membrane of Parakseneuridae and Kalligrammatidae (Fig. 29) [69], [81]. These hairs are impossible to study in detail, and it is unknown yet if these are long spinules or true sensilla. In some species of Parakseneuridae, long hairs cover almost entire wing membrane, denser in anterior and apical portions of the wing, and longer basally (Fig. 29).

The presence of long hairs on a restricted area of the hind wing of one species of the Eocene genus *Palaeopsychops* Andersen (Ithonidae s.l.) is considered as species autapomorphy [82].

#### Subcosta Anterior (Fig. 30)

**Figure 30:**
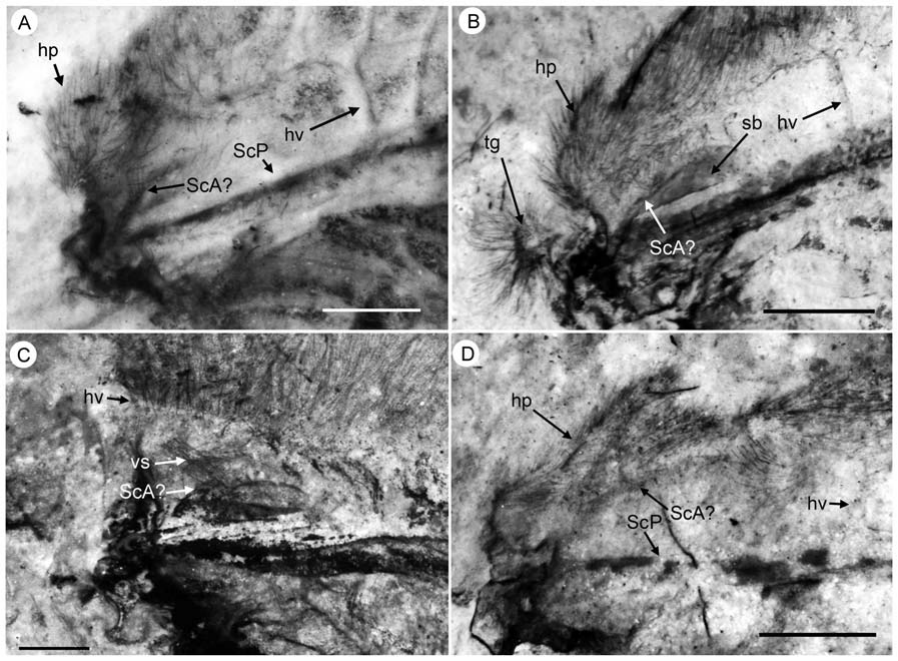
Humeral area of wings of Parakseneuridae. A, the forewing of *Pseudorapisma jurassicum* gen. et sp. nov., CNU-NEU-NN2011013; B, same, CNU-NEU-NN2011027PC; C, the forewing of *Parakseneura nigromacula* gen. et sp. nov., CNU-NEU-NN2011026PC; D, the hind wing of *Pseudorapisma maculatum* gen. et sp. nov., CNU-NEU-NN2011018 (all wetted with ethanol). Scale bar 1 mm.

A sclerotized bulge in the humeral area of both fore- and hind wings is usually well developed in the family (Fig. 30B, labeled *sb*). This bulge occurring only in Endoneoptera is considered by Kukalová-Peck and Lawrence [25] as the modified anterior subcosta (ScA). In the humeral area of the forewings of some Parakseneuridae two veinal structures are quite clearly visible: one margins a sclerotized bulge anteriorly (Fig. 30B, labeled *ScA?*), and another is located anteriorly to it (Fig. 30C, labeled *vs*). Both structures are also present in Kalligrammatidae (e.g., Figure 2 in [69]), and both can in principle be interpreted as ScA. In extant Neuroptera they are lost, and a sclerotized bulge is usually not margined by a veinal structure (for example in Ithonidae: Figure 5 in [25]). However, in the forewings of many Hemerobiidae there is rather well-developed such veinal structure (see for example Figures 13, 24 in [83]). This is probably homologous with the veinal structure bordered the sclerotized bulge of Parakseneuridae. Therefore, we interpret this veinal structure as ScA. In general, we follow here Kukalová-Peck and Lawrence [25], but consider a veinal structure bordered a sclerotized bulge to be ScA, not entire sclerotized bulge. This presumed ScA may not be confidently homologous with ScA of other Pterygota (e.g., orthopteroid orders). Moreover, it may be a secondary veinal structure. Nevertheless, the hypothesis of Kukalová-Peck and Lawrence [25] appears currently to be most reasonable, but it needs verification by examination of new well-preserved fossil materials.

#### Subcosta Posterior

In this family, ScP is apically fused with RA (*Parakseneura* gen. nov.) or not (*Shuraboneura* gen. nov., *Pseudorapisma* gen. nov.). It is generally thought that the latter condition is plesiomorphic, and the former is derived [24], [84]. Although it is likely correct in general for insects, the question might be more complex for particular families of Neuroptera. For example, the vast majority of extant members of Chrysopidae and Hemerobiidae have ScP and RA distally separated, but their oldest (Mesozoic) representatives (and their stem groups) often have ScP and RA distally fused. The same situation is in Ithonidae. Therefore, it is quite possibly that reverse evolution of this character (from ScP and RA fused to separated) might have occurred in some families. In Parakseneuridae, *Shuraboneura* gen. nov. is older than two other genera. Therefore, the condition of ScP and RA distally separate is most probably plesiomorphic in this family.

The ScP is relatively short in the fore- and hind wings of Parakseneuridae. Similar short ScP is often present in Neuroptera when ScP is not fused with RA, for example Panfiloviidae (e.g., Figure 4 in [36]), Ithonidae (e.g., Figure 2 in [2]), Psychopsidae (e.g., Figure 3B in [74]), Hemerobiidae. Noteworthy, the Kalligrammatidae have usually long ScP.

#### Radius Anterior

The distal curvature of RA towards the stem of RP is characteristic of the family, occurring in both the forewings (Fig. 27) and the hind wings (Fig. 26). Similar condition is only found in some Ithonidae (see Figure 2 in [2]).

#### Radius Posterior

The widely spaced branches of RP as found in some Parakseneuridae (Figs. 15, 26) occur rather rarely in the order. Of relatively large Neuroptera, such condition is known in Kalligrammatidae (e.g., Figures 1, 2 in [85]), Ascalochrysidae (Figure 2 in [11]), some Osmylidae (e.g., Figure 2 in [37]), and some non-classified Neuroptera (e.g., Figure 2 in [86]; Figure 2 in [87]).

#### Media Anterior

In the forewings, this vein is similarly configured in all species of Parakseneuridae: shallowly dichotomously branched. Such configuration is generally characteristic of most families of the order Neuroptera. Only in Permithonidae, MA is deeply dichotomously branched (e.g., Figure 2 in [88]). In the hind wings, MA is similarly configured as in the forewing.

#### Media Posterior

Such configuration of the forewing MP (deeply dichotomously branched) as found in *Shuraboneura* gen. nov. and *Parakseneura* gen. nov. is not characteristic of extant taxa. Only few genera of Kalligrammatidae (i.e., the Middle Jurassic *Protokalligramma* Yang *et al*.; an undescribed genus from the Late Jurassic of Karatau: Figure 254 in [89]; Figure 2 in [69]) have almost identically configured MP. Some Ithonidae have also similar branching (e.g., *Principiala* Makarkin & Menon, Figure 3 in [72]). The MP of the Permian Permithonidae is dichotomously branched. This condition is probably plesiomorphic, and the pectinate branching of MP found in some specimens of *Parakseneura* gen. nov. (see Figs. 9, 10, 19) is derived.

In the hind wings of Parakseneuridae, MP is always dichotomously branched, shallowly (Fig. 23) or deeply (Fig. 15). The latter condition is found in Ascalochrysidae (Figure 2 in [11]), some Kalligrammatidae (e.g., Figures 1, 2 in [85]), some Hemerobiidae (e.g., Figure 175 in [33]). In the majority of other Neuroptera, MP is shallowly pectinately or dichotomously branched.

#### Cubitus Anterior

CuA of the forewings is deeply dichotomously branched in all genera of Parakseneuridae. The similar branching is found in some Kalligrammatidae (e.g., *Protokalligramma*, and an undescribed genus from Karatau [69]), Aetheogrammatidae (e.g., Figure 3 in [10]), and some Psychopsidae (e.g., Figure 3A in [90]). In the hind wings of Parakseneuridae, CuA is generally pectinate, but its branches are dichotomously branched (Figs. 13, 23, 26). The similar branching is found in some Kalligrammatidae (e.g., Figure 1 in [91]; Figures 1, 2 in [85]), Panfiloviidae (e.g., Figure 4, 5 in [36]), and Grammolingiidae (e.g., Figure 1a in [92]).

#### Cubitus Posterior

The configuration of CuP in the forewing (deeply dichotomous) found in *Shuraboneura* gen. nov. and *Parakseneura* gen. nov. is characteristic of Brongniartiellidae, Osmylopsychopidae, the most generalized Kalligrammatidae (i.e., *Protokalligramma* Yang *et al*. and an undescribed genus from Karatau [69]), some Ithonidae and some genera of other families in which other branching types dominate (e.g., *Undulopsychopsis* Peng *et al*. in Psychopsidae; see Figure 3 in [74]). This type of CuP branching appears to be most primitive in the order, but in the Permian and outgroup taxa (Raphidioptera, Megaloptera) CuP is simpler configured, even in Corydalidae which has most rich venation of outgroup taxa. However, the hitherto known Permian taxa of Neuroptera are relatively small, and its CuP may be secondarily simplified.

In the hind wings, CuP is shallowly dichotomously branched (Figs. 13, 23, 26). The similar branching is found in Ithonidae and some Kalligrammatidae (e.g., Figure 1 in [91]). Unfortunately, the hind wings are unknown in Brongniartiellidae and Osmylopsychopidae, and poorly known in some other fossil families (e.g., Prohemerobiidae, Archeosmylidae).

#### Anal veins

**Figure 31:**
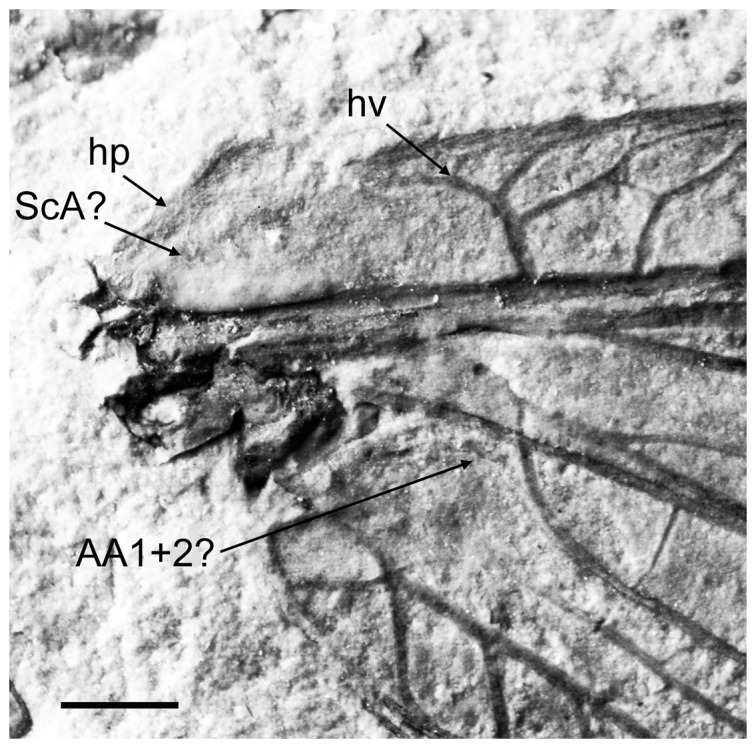
Basal portion of the hind wing of *Pseudorapisma jurassicum* gen. et sp. nov., paratype CNU-NEU-NN2011006PC (dry). Scale bar 1 mm.

The number and configuration of anal veins in Parakseneuridae appears to be most primitively in the superorder. This is the only known family in Neuropterida, which possesses a complete set of anal veins if the presence of AA1+2 is confirmed. In the forewing, the presumed AA1+2 is detected only in *Parakseneura* gen. nov. (Figs. 1B, 3C, 10B), in the hind wing only in *Pseudorapisma* gen. nov. (Fig. 31). In both genera the presumed AA1+2 is short and distally fused with AA3+4 (in the forewing) or terminated near CuP (in the hind wing). The presumed forewing AA1+2 has a common stem with AA3+4, whereas these veins in the hind wing are not stemmed. Three other anal veins (i.e., AA3+4, AP1+2, AP3+4) in the forewing are deeply forked each. Similar branching of these veins (but somewhat shallower) is known in Osmylopsychopidae (e.g., Figure 6 in [93]), some Kalligrammatidae (i.e., *Protokalligramma* Yang *et al*. and an undescribed genus from Karatau [69]), probably in Brongniartiellidae (incompletely preserved, see Figure 3 in [38]), and some Ithonidae (e.g., Figure 2E in [2]).

#### Summary

The venation of this family appears one of most primitive in the order by the presence of the presumed ScA and AA1+2, the dichotomous branching of MP, CuA, CuP, AA3+4, AP1+2, the sporadically arranged crossveins, the presence of the basal r-m brace in the hind wing. It possesses, however, some apomorphic conditions, e.g., the humeral veinlet is well developed, strongly recurrent, and the nygmata and ‘M5’ are lost. This analysis shows that Parakseneuridae is morphologically most similar to Ithonidae, the primitive Kalligrammatidae, Brongniartiellidae, and Osmylopsychopidae.

## Intergeneric Relationships in Parakseneuridae

Of the three genera of Parakseneuridae fam. nov., *Shuraboneura* gen. nov. is probably most primitive. Its forewing in general very similar to that of *Parakseneura* gen. nov. (i.e., similar size, shape, and general venational pattern). However, *Shuraboneura* gen. nov. has some important plesiomorphic conditions, e.g., ScP and RA are separate and the hind and outer wing margin is not undulate. This is well consistent with its older age.

*Pseudorapisma* gen. nov. and *Parakseneura* gen. nov. equally pretend to be a most advanced genus. The former genus possesses such apomorphic conditions as the absence of a basal sinuate crossvein between R and M systems in the hind wing and the presumed AA1+2 in the forewing. On the other hand, its ScP and RA are not fused and the presumed AA1+2 is present in the hind wing, plesiomorphic character states in the family. The venation of *Parakseneura* gen. nov. appears to be more plesiomorphic than *Pseudorapisma* gen. nov., but its ScP and RA are fused and the presumed AA1+2 is absent in the hind wing, apomorphic conditions in the family.

## The Phylogenetic Position of Parakseneuridae

### Results of Phylogenetic Analysis

**Figure 32:**
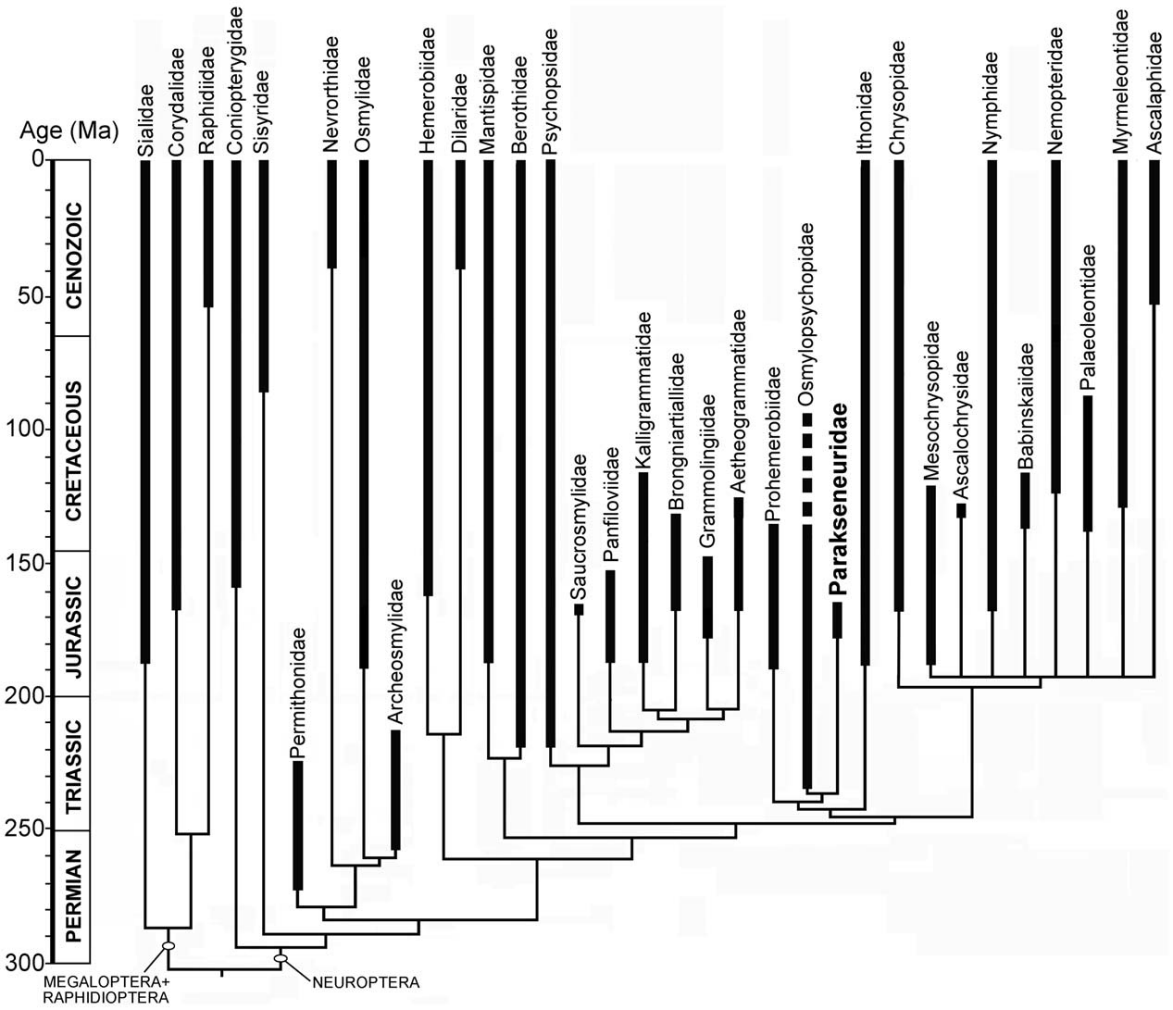
The phylogenetic relationships of 33 families of Neuropterida based on the combined morphological and DNA sequence data. Divergence time is inferred from the results of molecular divergence time estimates from [24]. Fossil age of each family is based on all available paleontological data, both published and unpublished.

A combined comparative analysis of morphological and DNA sequence data for 18 extant and 15 extinct families of Neuropterida recovered 26 most parsimonious trees (length = 3054; consistency index = 0.475; retention index = 0.362). The topology of the phylogram presented in Figure 32 reflects the results of this analysis, with ages of fossils included along with divergence time inferred from the results of molecular divergence time estimates from [24]. Reflecting the significant amount of missing data for fossil taxa, as well as the limited morphological scoring, the overall statistical support for most nodes on the tree is relatively very weak but well resolved, with only a single polytomy recovered in the clade comprising of families placed in Myrmeleontoidea (Nymphidae, Nemopteridae, Ascalaphidae, Myrmeleontidae, Palaeoleontidae and Babinskaiidae) and Chrysopoidea (Mesochrysopidae and Ascalochrysidae). Despite weak statistical support some clades appear quite reasonable and correspond to qualitative analyses.

#### Coniopterygidae

The position of this family as sister to other Neuroptera is expected and reasonable. Its constant morphology (e.g., minute size and great reduction of the venation) since the Late Jurassic implies that the family diverged from the other Neuroptera long before Jurassic. Small size of Coniopterygidae is possible reason of their absence in the fossil record before that time.

#### Sisyridae

The venation of this family together with its small size is most similar to that of some Permian representatives of Permithonidae. This was mentioned years ago [94]. Therefore, the basal position of Sisyridae in our phylogram is reasonable. Nevertheless, the definite adult fossils of this taxon are known only from earliest Eocene [95], although a larva was recorded from the Late Cretaceous Santonian [96].

#### ‘Osmyloid’ clade

The clade comprising Archeosmylidae, Osmylidae, Permithonidae and Nevrorthidae appears to be artificial. However, the close phylogenetic relationships between Archeosmylidae and Osmylidae are supported by discovery of a Middle Jurassic genus of Osmylidae similar to the Triassic Archeosmylidae [35]. The placement of Permithonidae and Nevrorthidae in this clade reflects only their isolated position in the order. Both families are undoubtedly basal in the order [17], [18], [97], but their phylogenetic relationships with other families are unknown yet. A group of families recovered in the phylogram under the psychopsid clade (i.e., Saucrosmylidae, Panfiloviidae and Grammolingiidae) might belong to this clade (see below).

#### ‘Hemerobioid’ clade

The clustering of Hemerobiidae and Dilaridae appears to be artificial and contradicts most other analyses by various authors (see below). Although their venation is rather similar, other features are dissimilar (the dilarid head, mouthparts, antennae and abdomen with long ovipositor strongly differ from those of Hemerobiidae). Hemerobiidae is usually clustered with Chrysopidae (e.g., [20], [23], [24]) sharing similar larval morphology (possibly, however, largely due to their similar habits), but these families are very far from each other by adult features. The Middle Jurassic undescribed genera that might be ancestral Hemerobiidae resemble psychopsoids and very unlike chrysopoids (pers. obs. of VM). Dilaridae are placed usually in the berothid clade ([17], [20], although see [24]) also based mainly on larval characters. However, the larger adult Dilaridae of more primitive genus *Dilar* Rambur have some common features with Ithonidae (e.g., the similarly constructed head and similarly configured venation) rather than with Berothidae. This means that the relationships of both Hemerobiidae and Dilaridae with other families are entirely unresolved yet.

#### Berothoid clade

The families Mantispidae and Berothidae (together with Rhachiberothinae and Mesithoninae) are clearly closely related and cluster in most other phylogenies [16]–[18], [24], [97]. This topology is supported here.

#### Psychopsoid clade

All families of this clade (plus Prohemerobiidae and Osmylopsychopidae) are sometimes treated as a separate taxon, either as the superfamily Psychopsoidea [98] or as the suborder Psychopsiformia [99]. Makarkin and Archibald [100] considered that the monophyly of Psychopsiformia (Psychopsoidea) is doubtful: the Prohemerobiidae probably does not belong to this suborder, and Panfiloviidae and Grammolingiidae are more similar to Osmylidae than to Psychopsidae. Therefore, it is not surprising that our phylogram recovered polyphyly of this group: the majority of families fall in the psychopsoid clade, but Prohemerobiidae and Osmylopsychopidae in the ithonoid clade.

The placement of Brongniartiellidae and Osmylopsychopidae in different clades may only be explained by the very poor knowledge (a few specimens were examined). Actually, these families appear to be closely related or even synonyms. It should be noted that limited number of genera selected for analysis of psychopsoid families makes difficult to understand their actual relationships. There are numerous diverse, fossil ‘psychopsid-like’ genera (including undescribed) that are at present difficult to classify. Most of these were not included in the analysis.

The clustering of Kalligrammatidae and Aetheogrammatidae in one clade with Psychopsidae appears to be quite reasonable. Kalligrammatidae and Psychopsidae were always considered closely related (e.g., [89], [101]). Aetheogrammatidae and Kalligrammatidae are very closely related families [10]; the former is likely a specialized branch of the latter [6], and in this case may be part of the latter. The discovery of the primitive aetheogrammatid genus in the Middle Cretaceous of China [102] supports this and shows that these two families differ only in some details of the wing venation. The mouthparts of both families are very elongate, specialized to feed on pollen and generative organs of extinct gymnosperms; cf. the mouthparts of Aetheogrammatidae (Figure on p. 26 in [103]) and Kalligrammatidae (Figure 1 in [70]). Therefore, these families are undoubtedly sister-groups.

Within this clade, the families Saucrosmylidae, Panfiloviidae and Grammolingiidae are closely related. Saucrosmylidae is more distant from the latter two in having more specialized venation (e.g., strongly dilated RA space with densely reticulated venation), but its general appearance is similar. These three families share similar size, color pattern, and similarly configured main veins. Analyses by other authors revealed relatively close relationships between the families Panfiloviidae, Grammolingiidae and Osmylidae [36], [100], and Saucrosmylidae only recently separated from Osmylidae [12], [104]. The venation of the oldest known grammolingiids from the Early/Middle Jurassic of Sai-Sagul revealed strong resemblance with that of Osmylidae, e.g., ScP and RA are distally fused in a manner similar to that of Osmylidae; the subcostal space is relatively narrow with few crossveins (AK, pers. obs.). Panfiloviidae and Grammolingiidae have very similar general venation, and are probably closely related, although not recovered as sister groups in this analysis.

The expected phylogeny based on the comparative analyses of characters and the recovered phylogram (Fig. 32) might differ mainly in the position of these families. We believe that the families Saucrosmylidae, Panfiloviidae and Grammolingiidae belong to the osmyloid clade. These families share with Osmylidae some principal characters, e.g., in the forewing, the costal space is strongly narrowed basally, with a simple, crossvein-like humeral veinlet; the branching of M is similar; Cu is forked very close to the wing base; in the hind wing, M is forked very close to the wing base. The similarity of the wings of these three families with those of psychopsoids (e.g., by numerous, very dense crossveins over wings, and a strong folding structure) is most probably convergent.

#### Ithonoid clade

The clustering of Ithonidae, Prohemerobiidae, Parakseneuridae and Osmylopsychopidae is largely concordant with previous estimates (see above). The venation of the Middle Jurassic genus *Jurapolystoechotes* Ren *et al*. is intermediate between typical Brongniartiellidae and the polystoechotid-like Ithonidae as indicated by the hind wing described by [86], and the forewings examined by C.F. Shi, VM and QY in CNUB. In general, the relationships of families of the ithonoid and psychopsoid clades are at present poorly understood because the vast majority of them are extinct and require further study in a comparative context. In this analysis the ithonoid clade is sister to Myrmeleontiformia excluding Psychopsoidea (i.e., Myrmeleontoidea and Chrysopoidea together), but it is quite possibly that the ithonoids and psychopsoids might be sister-groups when more fossil taxa are examined in detail.

#### Myrmeleontoid clade

The association of families of Chrysopoidea (Chrysopidae, Mesochrysopidae and Ascalochrysidae) and Myrmeleontoidea (other families of the clade) into single clade recovered by the phylogram is only seemingly strange. It was predicted by Ponomarenko [105] and Makarkin and Menon [106]. Ponomarenko [105] was of the opinion that a group of the Mesozoic genera similar to *Chrysoleonites* Martynov might be ancestral to both “chrysopoid” and “myrmeleotoid” lineages. Makarkin and Menon [106] developed this hypothesis in more detail. Indeed, the similarity of the venation of Chrysopoidea (especially Mesochrysopidae and Ascalochrysidae) and Myrmeleontoidea (especially some Nymphidae) is notable. The Jurassic genus *Chrysoleonites* Martynov (and some other similar genera) resembles both Mesochrysopidae and Nymphidae, but possesses a mixture of character states not allowing attributing it to neither Chrysopoidea nor Myrmeleontoidea.

MacLeod [107] stated that the larva of Chrysopidae “shows certain of the features usually associated with myrmeleontoid larval heads” (p. 194), mainly in the structure of the tentorium. Also, the female of all extant families of Myrmeleontoidea, Psychopsidae and Chrysopidae have single spermatheca, whereas other extant Neuroptera possess the paired spermatheca that is considered plesiomorphic state in Neuroptera [108]. The single spermatheca might be a synapomorphy of these three groups constituting a clade if the ithonoid sub-clade is excluded. However, of other Neuropterida only the subfamily Corydalinae (Corydalidae) possesses paired spermatheca, other groups have the single spermatheca (i.e., Raphidioptera, Sialidae, Corydalidae: Chauliodinae) [109], [110]. In insects in general the spermatheca is usually unpaired, and Contreras-Ramos [110] believes that this is plesiomorphic state, although Snodgrass [111] had an opinion that the spermatheca is possibly “primitively bifurcate or paired.” (p. 566).

The monophyly of Chrysopoidea appears to be very probably. Since Handlirsch [13] Mesochrysopidae and Chrysopidae have been considered closely related; their sister relationship shown by Nel and colleagues [32]. On the other hand, Mesochrysopidae and Ascalochrysidae are likely closely related, sharing many common features such as reduction of CuP in the hind wing [11]. However, our analysis did not recover a sister group relationship between either Mesochrysopidae and Ascalochrysidae, or Mesochrysopidae and Chrysopidae, so these hypotheses cannot be confirmed. Of the Jurassic representatives of the myrmeleontoid clade (i.e., Mesochrysopidae, Nymphidae, Chrysopidae, and the non-classified nymphid-like genera), Chrysopidae appear to be most morphologically distant from others and the reduction of the jugal lobe in the forewing may be considered as a synapomorphy of Myrmeleontoidea + Chrysopoidea exclusive of Chrysopidae (see Table S3). Unfortunately, the fossils of this clade are very rare in Early Jurassic localities; only discovery of new materials may help to resolve this question.

The extant families of Myrmeleontoidea (Nymphidae, Nemopteridae, Ascalaphidae, Myrmeleontidae) form the most reasoned clade in the order supported by several synapomorphies [24], although the position of the fossil families Palaeoleontidae and Babinskaiidae within this clade is unclear yet.

### The position of Parakseneuridae

According to our phylogram, the Parakseneuridae falls into the ithonoid clade as s sister family of Osmylopsychopidae. As mentioned above, Osmylopsychopidae are poorly known so this sister relationship should be considered as preliminary. The qualitative analysis presented shows that this family is morphologically most similar to Ithonidae, Kalligrammatidae, Osmylopsychopidae and Brongniartiellidae. These families belong to the ithonoid and psychopsoid clades.

### Conclusions

The venation of the large intriguing Middle Jurassic family Parakseneuridae displays a series of plesiomorphic characteristics. In particular, this is the only Neuropterida family, which possesses the presumed vein AA1+2 lost (or very indistinct) in other taxa of the superorder. Interestingly, all known Permian neuropterans do not have such primitive venation, probably because of their relatively small size. Both the phylogram and the comparative qualitative analysis revealed the position of Parakseneuridae near Ithonidae and Osmylopsychopidae (Brongniartiellidae). At present, only the relationships of families in the myrmeleontoid clade may be considered as relatively well resolved with strong statistical support. The relationships between other families are poorly resolved and vary in the phylogenies based on different characters. The reasons of this uncertainty can be explained by that fact that all divergences in the order at family level happened very long ago, in the Permian and Triassic (except for the myrmeleontoid clade which diverged in the Jurassic and Cretaceous time). Although we tried to choose in the morphological scoring only the ground plan characters states, it was sometimes impossible to do due to incompleteness of the fossil data and numerous venation reversals and parallelisms. The molecular data that might be the frame of the phylogeny of the order may also be unreliable because of long divergences. Neuroptera is ideally concordant with the situation where “shortness of time spans between divergences for evolution to occur and long time spans after divergences for subsequent evolutionary changes to obscure the earlier ones” [112, p. 449].

The uncertainty of phylogenetic position of many families of Neuroptera (including Parakseneuridae) is currently objective cause reflecting deficient palaeontological data, especially from critical important periods for the order, i.e., earliest Triassic and latest Triassic/earliest Jurassic. We strongly need the discovery and detailed examination of new fossil taxa.

## Supporting Information

Table S1 Description of morphological character states.
(PDF)

Table S2 Accession numbers of gene sequences for Neuropterida exemplars retrieved from Genbank.
(PDF)

Table S3 Morphological scoring for selected Neuropterida families.
(PDF)
