# Supplementary material for: A Remarkable New Family of Jurassic Insects (Neuroptera) with Primitive Wing Venation and Its Phylogenetic Position in Neuropterida
Source: PLoS One. 2012 Sep 18;7(9):e44762. doi: 10.1371/journal.pone.0044762 (PMC3445537; doi:10.1371/journal.pone.0044762)
Supplement: Table S1 — Description of morphological character states. (PDF) [file pone.0044762.s001.pdf]

A Remarkable New Family of Jurassic Insects (Neuroptera) with Primitive Wing Venation and its Phylogenetic Position in Neuropterida

Qiang Yang, Vladimir N. Makarkin, Shaun Winterton, Alexander V. Khramov, and Dong Ren

Table S1. Description of morphological character states.

LARVA:
Character 1: ***mouthparts***
(0) masticatory (as in Corydalidae: Figure 2b in [1]); (1) suctorial (as in Berothidae: Figure 8b in [1]).
Comments. State (1) is an autapomorphy of Neuroptera.

ADULT:
Character 2: ***palpi***
(0) moderately short (as in Parakseneuridae; Fig. 28A); (1) clearly elongate (as in Kalligrammatidae: Figure 249 in [2]).
Comments. State (1) is derived and probably a synapomorphy of Kalligrammatidae and Aetheogrammatidae, although the present analysis did not revealed sister relationships of these families.

Character 3: ***palpi***
(0) all segments nearly equal in thickness (as in Parakseneuridae; Fig. 28A); (1) terminal segment clearly enlarged (as in Coniopterygidae: Figure 7 in [3]).
Comments. State (1) is derived.

Character 4: ***coronal suture***
(0) distinct, long (as in Berothidae: Figure 34 in [4]); (1) indistinct, short (as in Sisyridae: Figure 8 A in [5]); (2) absent.
Comments. The coronal suture is the longitudinal median arm of the epicranial suture on posterior part of the head [6]. Coniopterygidae, Hemerobiidae, and probably Mesochrysopidae (maybe other families) are polymorphic for this character. In Coniopterygidae, the coronal suture is distinct from the posterior margin of the head to the labrum in Brucheiserinae (i.e., the coronal suture is continued in the mid-frontal suture), and indistinct or absent in other taxa [3,7]. In Hemerobiidae, the coronal suture is absent in many genera, but vestigial at least in *Micromus* Rambur and *Psectra* Hagen [8]. In Mesochrysopidae, this suture is detected only in one genus, *Longicellochrysa* Ren *et al.* [9].

Character 5: ***ocelli***
(0) present (as in Corydalidae: Figure 3A in [10]); (1) absent.
Comments. Osmylidae is polymorphic for this character; the ocelli are absent in the extant genus *Gumilla* Navás and present in the others.

Character 6: ***forelegs***
(0) walking; (1) raptorial (as in Mantispidae).
Comments. Berothidae are polymorphic for this character; the forelegs are raptorial in Rhachiberothinae, Paraberothinae, and probably Mesithoninae, and in the others.

Character 7: ***articulation of forelegs to prothorax***

(0) posteriorly (as in Berothidae: Figure 33 in [4]); (1) anteriorly (as in Mantispidae: Figures 10, 25 in [11]).

Comments. State (1) is an autapomorphy of Mantispidae.

Character 8: ***long ovipositor in female***

(0) absent; (1) present (as in Dilaridae: Figures on reverse of back wrapper in [12]).

Comments. A long hose-like ovipositor is the most spectacular apomorphic transformation of the female gonocoxites in Neuropterida occurring in all Raphidioptera and two families of Neuroptera (Mantispidae and Dilaridae) [13]. Mantispidae is polymorphic for this character: Symphrasinae possess an ovipositor, other subfamilies not. The ovipositor might be evolved independently in all three groups of Neuropterida; this is obvious concerning Raphidioptera and Neuroptera, and most probable concerning Mantispidae and Dilaridae as these families are not closely related as previously supposed.

Character 9: ***gonostyli of the 9th gonocoxites in female***

(0) present (as in Hemerobiidae: Figure 26 in [14]); (1) lost (as in Chrysopidae: Figures 127, 128 in [13]).

Comments. The presence of terminal gonostyli on the female ninth gonocoxites is a plesiomorphic condition in Neuropterida. It is believed that the loss of gonostyli have occurred several times independently in Neuroptera [13].

WINGS

Character 10: ***spinules ('microtrichia') on wing membrane***

(0) present (as in Hemerobiidae: Figure 12B in [15]); (1) absent.

Comments. Spinules (known also as 'microtrichia') are minute cuticle processes that are non-innervated and non-articulated in sockets [16]. Chrysopidae, Nymphidae and Berothidae are polymorphic for this character. The spinules cover the whole wing membrane in only one species of Chrysopidae (i.e., *Leptochrysa prisca* Adams et Penny [17]), and occur in reduced form only in *Nesydrion* Gerstaecker among Nymphidae [18]. State (1) is used for the analysis for these families. State (0) is used for Berothidae as the spinules are present in some genera [18].

Character 11: ***long hairs on wing membrane***

(0) absent; (1) present (as in Parakseneuridae; Fig. 29).

Comments. In Sialidae, both long true sensilla (setae, 'macrotrichia') and minute spinules ('microtrichia') occur on wing membrane [19], whereas in Ascalaphidae (some Haplogleniinae) only scarce sensilla are present [20]. In fossils, long hairs (where present) are impossible to study in detail, and it is unknown yet if these are long spinules or true sensilla. The presence of long hairs on a restricted area of the hind wing of one species of the Eocene genus *Palaeopsychops* Andersen (Ithonidae s.l.) is here considered as species autapomorphy.

Character 12: ***trichosors***

(0) present (as in Parakseneuridae); (1) absent (as in Chrysopidae).

Comments. The trichosors are small setigerous swellings along the wing margin between the tips of veins and veinlets [15]. These structures are characteristic of only Neuroptera. Mantispidae is polymorphic for this character; the trichosors are present in Mesomantispinae, Symphrasinae and some Drepanicinae, and absent in Calomantispinae and Mantispinae. It is believed that their presence is a plesiomorphic condition in the order.

Character 13: ***nygmata***

(0) present (as in Osmylidae: Figure 2C in [21]); (1) absent.

Comments. The nygmata ('facetic organs' [22], 'corneous spots' [23]) are small, thickened sensory spots on the wings. Their presence is a plesiomorphic condition as they occur in several orders of Holometobola (at least). Prohemerobiidae is possibly polymorphic for this character. The nygmata are not detected in *Prohemerobius dilaroides* Handlirsch, the type species of the genus and family, but present in some other species of this genus (e.g., Figure 2 in [24]).

Character 14: ***distal ScP and RA***
(0) separated (as in Parakseneuridae; Fig. 24C); (1) fused (as in Parakseneuridae; Fig. 1).

Comments. Many families of Neuroptera are polymorphic for this character (i.e., Berothidae, Chrysopidae, Hemerobiidae, Ithonidae, Kalligrammatidae, Panfiloviidae, Parakseneuridae, Permithonidae and Psychopsidae). ScP and RA are distally fused in some Mesozoic representatives of Limaiinae (Chrysopidae) [25; VM, QY, pers. obs.], and a few genera of Hemerobiidae [26,27]. The vast majority of genera of these families have these veins to be separate distally. ScP and RA are distally widely separated in only one genus of Kalligrammatidae (i.e., *Kalligrammina* Panfilov); this genus is though thought to be "incertae sedis in regard to family assignment" [28, p. 318]. In many extant Psychopsidae, the distal ScP abruptly terminates at RA or these veins are connected by a strong crossvein that closes distally so-called *vena triplica*. The majority fossil psychopsids have no *vena triplica*, and ScP and RA are distally clearly separated. In Osmylopsychopidae, ScP and RA approach each other distally, but their fusion is not clearly visible because of poor preservation.

In Coniopterygidae, ScP and RA appear widely separated distally. However, based on a study of the pupal tracheation, Withycombe [29] considered ScP deeply forked distally, whose anterior branch continues stem of ScP, and the posterior branch is basally crossvein-like and distally continues RA. Therefore, in fact both conditions occur on the same wing, ScP and RA are fused and separate. This unusual relationship of these veins needs more detailed examination. State (0) is used for this analysis.

FOREWING
Character 15: ***length***
(0) 0-5 mm; (1) 5-15 mm; (2) 15-35 mm; (3) >35 mm.

Comments. Mean forewing length in Mantispidae is approximately 15 mm; State (2) is used for this analysis.

Character 16: ***length/width ratio***
(0) <3.5; (1) >3.5.

Character 17: ***humeral veinlet***
(0) crossvein-like, simple (as in Chrysopidae; Figure 1 in [30]); (1) recurrent, branched (as in Parakseneuridae; Fig. 1).

Comments. The recurrent and branched humeral veinlet is characteristic of several families of Neuroptera. It has occurred (probably independently) in the hemerobiid, berothid, psychopsid and ithonid clades. Berothidae, Mantispidae, Prohemerobiidae and Hemerobiidae are polymorphic for this character. The berothid subfamily Mesithoninae possesses the strongly recurrent and branched humeral veinlet, whereas numerous other Mesozoic taxa have state (0) which is used here for the analysis. Some species of Prohemerobiidae have only a slightly recurrent and single forked humeral veinlet. In a few genera and species of Hemerobiidae (e.g., *Micromus* Rambur, *Zachobiella* Banks) the humeral veinlet is also only slightly recurrent and simple, or single forked. A few taxa of Mantispidae (e.g., *Plega* Navás

among Symphrasinae, Mesomantispinae) have state (1), but the majority of other genera (including the Early Jurassic *Liassochrysa* Ansorge et Schlüter) have state (0) which is used for the analysis.

Character 18: ***costal space***
(0) narrowed basally, moderately dilated some distance from base (as in Osmylidae: Figure 2A in [21]); (1) dilated in proximal part (as in Hemerobiidae: Figure 17 in [14]); (2) strongly dilated for entire length (as in Psychopsidae: Figure 32 in [31]); (3) equally slightly dilated for entire length (as in Palaeoleontidae: Figure 4B in [32]); (4) narrow for entire length (as in Coniopterygidae: Figure 8 in [3]).

Comments. Nymphidae, Prohemerobiidae, Ithonidae and Psychopsidae are polymorphic for this character. In Nymphidae, state (2) occurs in the most species of Myiodactylinae, and state (3) in the most species of Nymphinae and the vast majority of fossil taxa. In Prohemerobiidae, state (1) occurs in a few species that possess the strongly recurrent and branched humeral veinlet; state (0) in other species. The same situation is in Ithonidae. State (0) is used for the analysis in these families. In Psychopsidae, some fossil genera (e.g., *Undulopsychopsis* Peng *et al*: Figure 2 in [33]) have state (1), whereas all extant and some fossil genera (e.g., *Baisopsychops* Makarkin: Figure 1 in [34]) possess state (2), which is used for the analysis.

Character 19: ***width of subcostal space in its middle part***
(0) narrow (as in Hemerobiidae: Figures 24, 66, 90 in [14]); (1) moderately broad (as in Parakseneuridae: Fig. 1); (2) broad (as in Grammolingiidae: Figure 3 in [35]).

Comments. Osmylidae is polymorphic for this character. The subcostal space is narrow in most extant genera (state 0), and very broad in an undescribed Jurassic subfamily (state 2). However, the most primitive fossil taxa (including Protosmylinae, *Archaeosmylidia* Makarkin *et al*.: [36]) possess state (1) which is used here for the analysis.

Character 20: ***ScP***
(0) long, reaching pterostigmal region (as in Parakseneuridae; Fig. 1); (1) short, enters costal margin not reaching pterostigmal region (as in Raphidiidae).

Comments. Some Mesozoic taxa of Chrysopidae appear to posses state (1) (see e.g. Figure 2G in [37]). However, this is likely a derived condition within the family as ScP reaches the pterostigmal region in the vast majority of the other taxa (including Mesozoic forms).

Character 21: ***subcostal veinlets***
(0) all (or mostly) forked (as in Parakseneuridae: Fig. 1); (1) all (or mostly) simple (as in Ascalaphidae: Figures 36, 38 in [20]).

Comments. Nevrorthidae, Osmylidae, Nymphidae and Berothidae are polymorphic for this character. The subcostal veinlets in some species of Nevrorthidae are mostly forked (e.g., some *Nipponeurorthus* Nakahara; one undescribed species from Baltic amber), whereas in others they are mostly simple; state (0) is used for the analysis. State (1) is used for the analysis in Osmylidae, although some subcostal veinlets in many genera are forked. In some Nymphidae (e.g. *Myiodactylus* Brauer: [38]) the majority of subcostal veinlets are forked. In some Berothidae (e.g., the Burmese amber genera: Figures 19, 24, 25 in [39]) all subcostal veinlets are simple.

Character 22: ***subcostal crossveins (sc-r)***

(0) few (1-4, as in Hemerobiidae: Figures 33, 90, 166 in [14]); (1) many (>4, as in Parakseneuridae: Fig. 1); (2) very numerous (as in Kalligrammatidae: Figure 5 in [40]).

Comments. Dilaridae, Osmylidae and Nymphidae are polymorphic for this character. In Dilaridae, the genus *Dilar* Rambur possesses many subcostal crossveins (state 0), whereas *Nallachius* Navás and *Neonallachius* Nakahara have few crossveins; the latter are considered here as having secondarily simplified venation, and state (0) is used for the analysis. The vast majority of the Osmylidae genera have state (0), a few others have the states (1) (i.e., *Archaeosmylidia* Makarkin *et al*. [36], *Osmylochrysa* Jepson *et al*. (Figure 11 in [42]), and *Porismus* McLachlan (Figure 1 in [41]) and (2) (i.e., an undescribed genus of the Jurassic Daohugou; VM, QY, pers. obs.). This character varies in the extant Nymphidae, e.g., state (0) in *Norfolius* Navás and some *Nymphes* Leach, and state (1) in other species of *Nymphes* and *Myiodactylus* [38]. However, there are few subcostal crossveins in almost all fossil Nymphidae.

Character 23: ***RA (or ScP+RA) termination***
(0) well before wing apex (as in Parakseneuridae); (1) at or near wing apex (as in Mesochrysopidae; [43] Makarkin & Menon 2005, fig. 3) (2) well after wing apex (as in Ascalaphidae; [20] Tjeder 1992, figs. 36, 38).

Comments. State (2) is a synapomorphy of Myrmeleontoidea.

Character 24: ***RP origin***
(0) near wing base (as in Palaeoleontidae: Figure 4B in [32]); (1) distant from wing base (as in Mesochrysopidae: Figure 3 in [43]); (2) far from wing base (as in Ascalaphidae: Figures 36, 38 in [20]); (3) very far from wing base (as in Babinskaiidae: Figure 8A in [44]).

Character 25: ***RP1 branching***
(0) shallow (as in Parakseneuridae; Fig. 1); (1) deep (as in Hemerobiidae: Figures 74, 140 in [14]).

Comments. Prohemerobiidae, Psychopsidae and Hemerobiidae are polymorphic for this character. RP1 varies in the extant genera of Hemerobiidae from simple for the most length (e.g., *Hemerobius* Linnaeus) to deeply pectinate branched (e.g., *Conchopterella* Handschin) [14]. The most Mesozoic and Palaeogene taxa, however, possess state (1) (see Figure 4 in [45]; Figure 31 in [46]; Figures 4, 5 in [27]). In a few species of Prohemerobiidae and Psychopsidae, RP1 is deeply forked (see e.g. Figure 3 in [33]), but the majority of taxa have state (0).

Character 26: ***crossveins in radial space***
(0) very rare (as in Sisyridae: Figures 10A, 11A in [5]); (1) rare (as in Nevrorthidae: Plate 7, Figures 1, 2 in [47]); (2) dense (as in Parakseneuridae: Fig. 1); (3) very dense (as in Kalligrammatidae: Figure 5 in [40]).

Comments. Berothidae, Dilaridae, Osmylidae and Mesochrysopidae are polymorphic for this character. The crossveins in most genera of Berothidae (including most ancient ones) are rare, but dense in the subfamilies Nyrminae and Berothimerobiinae. The crossveins in small-sized Dilaridae (*Nallachius*, *Neonallachius*) are very rare, but this is a derived condition within the family. The crossveins in most genera of Osmylidae are quite dense to very dense (e.g., an undescribed genus of the Jurassic Daohugou; VM, QY, pers. obs.); but state (1) is characteristic of most putative primitive taxa (i.e., *Archaeosmylidia* Makarkin *et al*., Mesosmylininae, Protosmylinae) and is used for the analysis. The crossvein number in Mesochrysopidae is quite variable between taxa (see Figures 198, 110 in [48]; Figures 6, 7, 9

in [30]). The venation of *Mesochrysopa* Handlirsch is considered here as most primitive within the family, and its state (2) is used for the analysis.

Character 27: ***arrangement of crossveins in radial space***
(0) all crossveins sporadically distributed, not arranged in series (as in Ascalaphidae: Figures 36, 38 in [20]); (1) most crossveins sporadically distributed but some form gradate series (as in Brongniartiellidae: Figure 3B in [49]); (2) most crossveins arranged in gradate series, but there are some sporadically distributed crossveins (as in Osmylidae: Figure 2 in [50]); (3) all crossveins arranged in one or more gradate series (as in Hemerobiidae: Figures 33, 41, 50 in [14]).

Comments. Berothidae, Osmylidae, Mesochrysopidae are polymorphic for this character. In Berothidae, all four conditions are found but the majority of genera possess state (3) which is used in the analysis. In Osmylidae, states (0) to (2) occur; state (2) characteristic of most primitive taxa (*Archaeosmylidia* Makarkin *et al*., Mesosmylininae, Protosmylinae) is used for analysis. The crossvein arrangement in Mesochrysopidae is highly variable between taxa (see Figures 108, 110 in [48]; Figures 6, 7, 9 in [30]). State (2) characteristic of *Mesochrysopa* is used for the analysis.

Character 28: ***aligned basal oblique crossveins r-m and m-cu***
(0) absent; (1) present (as in Ascalaphidae: Figures 36, 38 in [20]).

Comments. The presence of aligned basal oblique crossveins r-m and m-cu is probably synapomorphy of the advanced Myrmeleontoidea (Myrmeleontidae, Ascalaphidae). Nymphidae is polymorphic for this character; in the Mesozoic taxa, these crossveins are not aligned.

Character 29: ***M and R***
(0) separate for entire length; (1) fused basally for considerably distance (as in Mantispidae: Figure 5A in [51]).

Character 30: ***MP termination***
(0) after wing mid-point; (1) before or at wing mid-point.

Character 31: ***MP branching***
(0) dichotomous, or deeply forked (as in Parakseneuridae: Fig. 1; Permithonidae: Figure 3b in [52]); (1) pectinate (as in Ithonidae: Figures 2B-D in [54]); (2) strongly pectinate (as in Saucrosmylidae: Figure 3A in [55]).

Comments. Coniopterygidae and Kalligrammatidae are polymorphic for this character. In Coniopterygidae, all three states appear occur if assumed that MA is reduced. State (1) is used for the analysis as it is characteristic of the most genera of Aleuropteryginae, a more ancient subfamily and more diverse in the fossil record than Coniopteryginae (see Table 1 in [56]). Most genera of Kalligrammatidae have a pectinate branched MP with branches anteriorly directed, clearly a derived condition within the family; state (0) is characteristic of *Protokalligramma* Yang *et al.* and an undescribed genus from Karatau [40] and is used for the analysis. In Sialidae, state (0) is used for the analysis, but some species have the simple MP.

Character 32: ***m-cu arculus ('M5')***
(0) distinct (as in Nevrorthidae: Plate 7, Figure 1 in [47]); (1) lost or not discernible from crossveins (as in Ascalaphidae: Figures 36, 38 in [20]).

Comments. A convex oblique vein connecting the stem of M and CuA is plesiomorphically present in almost all Neoptera (see for example Figures 1A, B in [57] for

this vein in the forewing of a basal blattinopsidaean from Carboniferous). In Neuroptera, this vein is well developed in the Permian Permithonidae (see Figures 2, 3, 6 in [58]) and Mesozoic Mesithoninae (Berothidae) (see Figures 1, 2 in [59]). The families Ithonidae and Osmylidae are polymorphic for this character. The m-cu arculus is not discernible from crossveins in most Osmylidae, but distinct in some Jurassic taxa (e.g. *Archaeosmylidia* Makarkin *et al*., and an undescribed genus from Daohugou [36], VM, QY, pers. obs.), especially in the latter taxon. The same situation is in Ithonidae where the m-cu arculus is distinct in some genera (e.g., *Platystoechotes* Carpenter), and not discernible from crossveins in the others (e.g., *Rapisma* Walker), see Figure 2 in [54].

Character 33: ***M and CuA***
(0) separate for entire length; (1) fused for some distance (as in Sialidae: Figure 1 in [19]).
Comments. The partial fusion of M and CuA occurring in all Raphidioptera (including Priscaenigmatidae) and Sialidae probably evolved independently.

Character 34: ***MP and CuA***
(0) separate; (1) fused (as in Ascalaphidae: Figures 36, 38 in [20]).
Comments. The fusion of MP and CuA in the forewing is a synapomorphy of Myrmeleontoidea (but it is unclear concerning Babinskaiidae due to small examined specimens). The similar character state found in some genera of other families (e.g., some *Nallachius* among Dilaridae: Figure 1 in [60]) undoubtedly evolved independently.

Character 35: ***CuA branching***
(0) dichotomous, or deeply forked (as in Parakseneuridae; Fig. 1); (1) pectinate (as in Hemerobiidae: Figure 33 in [14]); (2) strongly pectinate (as in Nymphidae: Figures 1, 24 in [38]); (3) simple (as in Coniopterygidae: Figure 8 in [3]).
Comments. Berothidae, Dilaridae, Grammolingiidae, Kalligrammatidae and Mantispidae are polymorphic for this character. Some genera or species of Berothidae, Mantispidae Mantispidae and Dilaridae have CuA not pectinate (dichotomous or so). In the most genera of Kalligrammatidae, CuA is usually weakly pectinate; state (0) characteristic of *Protokalligramma* and an undescribed genus from Karatau [40] is used for the analysis. The pectinate branches of the Grammolingiidae species are long and dichotomously branched.

Character 36: ***CuP branching***
(0) simple or with terminal fork (as in Permithonidae: Figures 1b, 2 in [52]); (1) dichotomous or deeply forked (as in Parakseneuridae; Fig. 1); (2) few pectinate (as in Mesochrysopidae: Figure 9.3 in [30]); (3) strongly pectinate (as in Nymphidae: Figures 1, 24 in [38]).
Comments. Kalligrammatidae, Mantispidae, Nymphidae and Psychopsidae are polymorphic for this character. In the most genera of Kalligrammatidae, CuA is usually weakly or strongly pectinate; state (1) is characteristic of *Protokalligramma* and an undescribed genus from Karatau [40] is used for the analysis. Some taxa of Mantispidae have CuP dichotomously branched (e.g., *Mesomantispa* Makarkin) or with terminal fork (e.g., some Symphrasinae). In some species of Nymphidae, CuP is only weakly pectinate. Some genera of Psychopsidae have CuP to be strongly dichotomously branched (e.g., *Undulopsychopsis*: Figures 2 in [33]; *Baisopsychops*: Figure 1 in [34]).

Character 37: ***AA3+4 branching***
(0) simple or with terminal fork (as in Chrysopidae: Nothochrysinae: Figures 1-5 in [61]); (1) dichotomous or deeply forked (as in Parakseneuridae: Fig. 1); (2) pectinate (as in Osmylidae: Figures 1-3 in [50]).

Comments. Berothidae, Ithonidae, Kalligrammatidae and Nymphidae are polymorphic for this character. In some genera or species of Berothidae and Ithonidae, AP1+2 is pectinate. Most species of Kalligrammatidae have AA3+4 more or less pectinate; state (1) characteristic of *Protokalligramma* and an undescribed genus from Karatau [40] is used for the analysis. In the genera of Myiodactylinae, AA3+4 is pectinate, in the other genera of Nymphidae it is deeply or shallowly forked; state (1) is used for the analysis.

Character 38: ***AP1+2 branching***
(0) simple or with terminal fork (as in Chrysopidae: Nothochrysinae: Figures 1-5 in [61]); (1) dichotomous or deeply forked (as in Parakseneuridae: Fig. 1); (2) pectinate (as in Osmylidae: Figures 1-3 in [50]).

Comments. Kalligrammatidae and Sisyridae are polymorphic for this character. In some genera of Kalligrammatidae (e.g., *Sophogramma* Ren et Guo), the AP1+2 is deeply forked, not pectinate. In Sisyridae, this vein is few-pectinate or has terminal fork; state (2) is used in the analysis.

Character 39: ***jugal lobe***
(0) distinct (as in Chrysopidae: Nothochrysinae: Figures 1-5 in [61]); (1) absent or reduced (as in Ascalaphidae: Figure 38 in [20]).

Comments. The full reduction of the anal lobe occurs probably only in families of the myrmeleontoid clade (except Chrysopidae). However, it evolved also independently within some other families in advanced genera (e.g., in Coniopterygidae, Berothidae).

HIND WING

Character 40: ***hind wings***
(0) normally developed; (1) very narrow and long (as in Nemopteridae: Figures 1987, 2023 in [62]).

Comments. Neuroptera normally bear well-developed hind wings more or less similar in size to forewing, but there are a few brachypterous, micropterous or apterous species, found in the following extant families: Hemerobiidae, Coniopterygidae, Ithonidae, Dilaridae, Berothidae, and Nemopteridae [63,64]. Such reduction is usually associated with flightlessness, and obviously derived within genera or generic groups. The highly modified hind wings of Nemopteridae are an autapomorphy of this family.

Character 41: ***humeral veinlet***
(0) crossvein-like, simple; (1) recurrent, branched (as in Parakseneuridae: Fig. 31).

Comments. The humeral veinlet of Psychopsidae is recurrent but not branched; state (1) is used for the analysis.

Character 42: ***basal r-m brace***
(0) long, sinuate (as in Hemerobiidae: Figures 42, 51, 61 in [14]); (1) short straight or absent (as in Ascalaphidae: Figures 36, 38 in [20]).

Comments. Berothidae and Mantispidae are polymorphic for this character. The basal r-m brace is long and sinuate in Mesithoninae and Rhachiberothinae among Berothidae, and Symphrasinae among Mantispidae, whereas in other taxa of these families it is straight, crossvein-like. State (0) is used for analysis.

Character 43: ***'M5'***

(0) long (as in Ascalochrysidae: Figure 3 in [65]); (1) short, crossvein-like (as in Hemerobiidae: Figure 150 in [14]); (2) lost or not discernible from crossveins (as in Parakseneuridae: Fig. 2C).

Comments. The long ‘M5’ occurring in the families Permithonidae (see Plate 1, Figure 1 in [66]), Nevrorthidae (see Plate 8, Figures 1, 2 in [47]) and Ascalaphidae is considered plesiomorphic [65].

Character 44: ***CuP***

(0) well developed, complete; (1) strongly reduced (as in Ascalochrysidae; Figure 3 in [65]).

Comments. Mantispidae is polymorphic for this character; the complete CuP is present only in Symphrasinae, and strongly reduced in others. CuP of Berothidae varies in its appearance in different genera from the reduction of its middle part (basal and distal parts are present) to entire loss.

References to Table S1

1. Gepp J (1984) Erforschungsstand der Neuropteren-Larven der Erde (mit einem Schlüssel zur larvaldiagnose der Familien, einer Übersicht von 340 beschreibenen Larven und 600 Literaturzitaten). In: Gepp J, Aspöck H, Hölzel H, eds. Progress in World's Neuropterology. Proceedings of the First International Symposium on Neuropterology. Graz, Austria: Privately printed. pp. 183–239.
2. Ponomarenko AG (2002) Superorder Myrmeleontidea Latreille, 1802 (=Neuropteroidea Handlirsch, 1903). In: Rasnitsyn AP, Quicke DLJ, eds. History of Insects. Dordrecht: Kluwer Academic Publishers. pp. 176–192.
3. Meinander M (1972) A revision of the family Coniopterygidae (Planipennia). Acta Zool Fenn 136: 1–357.
4. Aspöck U, Aspöck H (1997) Studies on new and poorly-known Rhachiberothidae (Insecta: Neuroptera) from subsaharan Africa. Ann Nat Mus Wien Ser B 99: 1–20.
5. Parfin SI, Gurney AB (1956) The spongilla-flies, with special reference to those of the western hemisphere (Sisyridae, Neuroptera). Proc US Natl Mus 105: 421–529.
6. Snodgrass RE (1935) Principles of Insect Morphology. McGraw-Hill Book Co., New York. 667 p.
7. Sziráki G (2007) Studies on Brucheiserinae (Neuroptera: Coniopterygidae), with description of the second genus of the subfamily. Acta Zool Acad Sci Hung 53(Suppl. 1): 231–254.
8. Tjeder B (1961) Neuroptera-Planipennia. The Lace-wings of Southern Africa. 4. Family Hemerobiidae. In: Hanström B, Brinck P, Rudebec G, eds. South African Animal Life. Vol. 8. Uppsala: Almqvist & Wiksells Boktryckri Ab. pp. 296–408.
9. Ren D, Makarkin VN, Yang Q (2010) A new fossil genus of Mesochrysopidae (Neuroptera) from the Early Cretaceous Yixian Formation of China. Zootaxa 2523: 50–56.
10. Liu XG, Hayashi F, Yang D (2009) Notes on the genus *Protohermes* van der Weele (Megaloptera: Corydalidae) from Vietnam, with description of two new species. Zootaxa 2146: 22–34.
11. Willmann R (1990) The phylogenetic position of the Rhachiberothinae and the basal sister-group relationships within the Mantispidae (Neuroptera). Syst Entomol 15: 253–265.
12. Makarkin VN, Tshistjakov YA (2009) The Dilaridae (Neuroptera): poorly known «pleasing» lacewings. Eversmannia 19–20: 36–47. (In Russian, English abstract).

13. Aspöck U, Aspöck H (2008) Phylogenetic relevance of the genital sclerites of Neuropterida (Insecta: Holometabola). Syst Entomol 33: 97–127.
14. Oswald JD (1993a) Revision and cladistic analysis of the world genera of the family Hemerobiidae (Insecta: Neuroptera). J N Y Entomol Soc 101: 143–299.
15. Killington FJ (1936) A monograph of the British Neuroptera. Vol. 1. London: Ray Society. xix + 269 p.
16. Vshivkova TS, Makarkin VN (2010) Ultrastructural morphology of leg cuticle derivatives useful for phylogenetic study of Neuropterida (Insecta: Megaloptera, Neuroptera): preliminary report. In: Devetak D, Lipovšek S, Arnett AE, eds. Proceedings of the Tenth International Symposium on Neuropterology, Piran, Slovenia, 2008. Maribor, Slovenia. pp. 278–300.
17. Adams PA, Penny ND (1992) New genera of Nothochrysinae from South America (Neuroptera: Chrysopidae). Pan-Pac Entomol 68: 216–221.
18. Riek EF (1967) Structures of unknown, possibly stridulatory, function of the wings and body of Neuroptera; with an appendix on other endopterygote orders. Aust J Zool 15: 337–348.
19. Adams PA (1958) The relationship of the Protoperlaria and the Entopterygota. Psyche 65: 115–127.
20. Tjeder B (1992) The Ascalaphidae of the Afrotropical Region (Neuroptera). 1. External morphology and bionomics of the family Ascalaphidae, and taxonomy of the subfamily Haplogleniinae including the tribes Proctolyrini n. tribe, Melambrotini n. tribe, Campylophlebini n. tribe, Tmesibasini n. tribe, Allocormodini n. tribe, and Ululomyiini n. tribe of Ascalaphidae. Entomol Scand, Suppl 41: 3–169.
21. Wang Y, Liu Z, Ren D, Shih C (2011) New Middle Jurassic kempynin osmylid lacewings from China. Acta Palaeontol Pol 56: 865–869.
22. Martynov AV (1925[1924]) On the facetic organs on the wings of insects. Trudy Leningrad Obshch Estestvoispyt 54(2): 3–24. (In Russian).
23. Londt JGH (1974) 'Corneus spots' in insects. J Entomol Soc South Afr 37: 5–14.
24. Khramov AV (2011) Two new lacewings (Neuroptera) from the Upper Jurassic locality Shar-Teg (Mongolia). Paleontol J 45(2): 174–178.
25. Makarkin VN (1997a) Fossil Neuroptera of the Lower Cretaceous of Baisa, East Siberia. Part 3. Chrysopidae. Spixiana 20: 107–118.
26. Oswald JD (1994) A new phylogenetically basal subfamily of brown lacewings from Chile (Neuroptera: Hemerobiidae). Entomol Scand 25: 295–302.
27. Makarkin VN, Archibald SB, Oswald JD (2003) New Early Eocene brown lacewings from western North America (Neuroptera: Hemerobiidae). Can Entomol 135: 637–653.
28. Engel MS, Huang DY, Lin QB (2011) A new genus and species of Aetheogrammatidae from the Jurassic of Inner Mongolia, China (Neuroptera). J Kansas Entomol Soc 84: 315–319.
29. Withycombe CL (1922) The wing venation of the Coniopterygidae. Entomologist 55: 224–225.
30. Nel A, Delclòs X, Hutin A (2005) Mesozoic chrysopid-like Planippenia: a phylogenetic approach (Insecta: Neuroptera). Ann Soc Entomol Fr 41: 29–68.
31. Oswald JD (1993b) Phylogeny, taxonomy, and biogeography of extant silky lacewings (Insecta: Neuroptera: Psychopsidae). Mem Am Entomol Soc 40: iii+1–65.
32. Menon F, Makarkin VN (2008) New fossil lacewings and antlions (Insecta, Neuroptera) from the Lower Cretaceous Crato Formation of Brazil. Palaeontology 51: 149–162.
33. Peng YY, Makarkin VN, Wang XD, Ren D (2011) A new fossil silky lacewing genus (Neuroptera: Psychopsidae) from the Early Cretaceous Yixian Formation of China. ZooKeys 130: 217–228.

34. Makarkin VN (1997b) Fossil Neuroptera of the Lower Cretaceous of Baisa, East Siberia. Part 4. Psychopsidae. Beitr Entomol 47: 489–492.
35. Ren D (2002) A new lacewing family (Neuroptera) from the Middle Jurassic of Inner Mongolia, China. Entomol Sin 9(4): 53–67.
36. Makarkin VN, Yang Q, Ren D. in press. A new basal osmylid neuropteran insect from the Middle Jurassic of China linking Osmylidae to the Permian-Triassic Archeosmylidae. Acta Palaeontol Pol: doi: http://dx.doi.org/10.4202/app.2011.0018
37. Martins-Neto RG (2003[2002]) The Santana Formation Paleoentomofauna reviewed. Part I - Neuropteroida (Neuroptera and Raphidioptera): systematic and phylogeny, with description of new taxa. Acta Geol Leopold 25(55): 35–66.
38. New TR (1981) A revision of the Australian Nymphidae (Insecta: Neuroptera). Aust J Zool 29: 707–750.
39. Engel MS, Grimaldi DA (2008) Diverse Neuropterida in Cretaceous amber, with particular reference to the paleofauna of Myanmar (Insecta). Nova Suppl Entomol 20: 1–86.
40. Yang Q, Makarkin VN, Ren D (2011) Two interesting new genus of Kalligrammatidae (Neuroptera) from the Middle Jurassic of Daohugou, China. Zootaxa 2873: 60–68.
41. New TR (1983) Revision of the osmylid subfamilies Porisminae and Eidoporisminae (Insecta: Neuroptera). Aust J Zool 31: 763–770.
42. Jepson JE, Makarkin VN, Coram RA (2012) Lacewings (Insecta: Neuroptera) from the Lower Cretaceous Purbeck Limestone Group of southern England. Cretaceous Res 34: 31–47.
43. Makarkin VN, Menon F (2005) New species of the Mesochrysopidae (Insecta, Neuroptera) from the Crato Formation of Brazil (Lower Cretaceous), with taxonomic treatments of the family. Cretaceous Res 26: 801–812.
44. Martins-Neto RG. Vulcano MA (1989) Neurópteros (Insecta: Planipennia) da Formação Santana (Cretáceo Inferior), Bacia do Araripe, Nordeste do Brasil. I - Família Chrysopidae. An Acad Bras Cienc 60: 189–201.
45. Henriksen KL (1922) Eocene insects from Denmark. Danmarks Geol Undersøgelse (2) 37: 1–36.
46. Jarzembowski EA (1980) Fossil insects from the Bembridge Marls, Palaeogene of the Isle of Wight, southern England. Bull Brit Mus Nat Hist (Geol) 33: 237–293.
47. Nakahara W (1958) The Neurorthinae, a new subfamily of the Sisyridae (Neuroptera). Mushi 32: 19–32.
48. Panfilov DV (1980) New representatives of lacewings (Neuroptera) from the Jurassic of Karatau. In: Dolin VG, Panfilov DV, Ponomarenko AG, Pritykina LN. Fossil insects of the Mesozoic. Kiev: Naukova Dumka. pp. 82–111. (In Russian).
49. Makarkin VN (2010) New psychopsoid Neuroptera from the Lower Cretaceous of Baissa, Transbaikalia. Ann Soc Entomol Fr 46: 254–261.
50. Carpenter FM (1943) Osmylidae of the Florissant shales, Colorado (Insecta-Neuroptera). Am J Sci 241: 753–760.
51. Poivre, C. 1978. Morphologie externe comparée de *Gerstaeckerella gigantea* Enderlein [Planipennia, Mantispidae]. Ann Soc Entomol Fr 14: 191–206.
52. Novokshonov VG (1996) Systematic position of some Upper Permian Myrmeleontida (=Neuroptera; Insecta). Paleontol J 30: 38–45.
54. Winterton S, Makarkin VN (2010) Phylogeny of moth lacewings and giant lacewings (Neuroptera: Ithonidae, Polystoechotidae) by using DNA sequence data, morphology, and fossils. Ann Entomol Soc Am 103: 511–522.
55. Ren D, Yin JC (2003) New 'Osmylid-like' fossil Neuroptera from the Middle Jurassic of Inner Mongolia, China. J N Y Entomol Soc 111: 1–11.

56. Engel MS (2002) A new dustywing (Neuroptera: Coniopterygidae) in Turonian amber from New Jersey, with a reassessment of *Glaesoconis* in Neoconian amber from Lebanon. J Kansas Entomol Soc 75: 38–42.
57. Béthoux O, Jarzembowski EA (2010) New basal neopterans from Writhlington (UK, Pennsylvanian). Alavesia 3: 87–96.
58. Vilesov AP (1995) Permian lacewings (Insecta: Myrmeleontida) from the Chekarda locality (Ural). Paleontol J 29(2): 115–129.
59. Makarkin VN (1999) Fossil Neuroptera of the Lower Cretaceous of Baisa, East Siberia. Part 6. Mesithonidae (Insecta). Neues Jahrb Geol Paläontol Monatshefte 1999(12): 705–712.
60. Penny ND (1994) A new species of *Nallachius* (Neuroptera: Dilaridae) from Costa Rica. Pan-Pac Entomol 70: 309–312.
61. Adams PA (1967) A review of the Mesochrysinae and Nothochrysinae (Neuroptera: Chrysopidae). Bull Mus Comp Zool 135: 215–238.
62. Tjeder B (1967) Neuroptera-Planipennia. The Lace-wings of Southern Africa. 6. Family Nemopteridae. In: Hanström B, Brinck P, Rudebec G, eds. South African Animal Life. Vol. 13. Uppsala: Almqvist & Wiksells Boktryckri Ab. pp. 290–501.
63. Oswald JD (1996) A new brachypterous *Nusulala* species from Costa Rica, with comments on the evolution of flightlessness in brown lacewings (Neuroptera: Hemerobiidae). Syst Entomol 21: 343–352.
64. Pantaleoni RA, Letardi A (1996) A remarkable brachypterous female of Dilaridae (*Dilar parthenopaeus* Costa ?) (Neuroptera). In: XX International Congress of Entomology, Firenze, Italy, August 25-31 1996. Proceedings. p. 01-242
65. Ren D, Makarkin VN (2009) Ascalochrysidae – a new lacewing family from the Mesozoic of China (Insecta: Neuroptera: Chrysopoidea). Cretaceous Res 30: 1217–1222.
66. Novokshonov VG (1997) Early Evolution of Scorpionflies (Insecta: Panorpida). Moscow: Nauka Press. 140 p. (In Russian).
