# Supplementary material for: A Remarkable New Family of Jurassic Insects (Neuroptera) with Primitive Wing Venation and Its Phylogenetic Position in Neuropterida
Source: PLoS One. 2012 Sep 18;7(9):e44762. doi: 10.1371/journal.pone.0044762 (PMC3445537; doi:10.1371/journal.pone.0044762)
Supplement: Table S2 — Accession numbers of gene sequences for Neuropterida exemplars retrieved from Genbank. (PDF) [file pone.0044762.s002.pdf]

A Remarkable New Family of Jurassic Insects (Neuroptera) with Primitive Wing Venation and its Phylogenetic Position in Neuropterida

Qiang Yang, Vladimir N. Makarkin, Shaun Winterton, Alexander V. Khramov, and Dong Ren

Table S2. Accession numbers of gene sequences for Neuropterida exemplars retrieved from Genbank (http://www.ncbi.nih.gov/). Voucher numbers identify individual specimens deposited in the California Academy of Sciences, San Francisco, USA.

			**Genbank accession**			
**ORDER/ family**	**Species**	**Specimen voucher number**	**16S**	**COI**	**18S**	**CAD**
**MEGALOPTERA**						
**Corydalidae**	*Nigronia serricornis* (Say)	CASENT8092157	EU734881	EU839748	EU815263	EU860133
**Sialidae**	*Sialis* nr. *mohri* Ross	CASENT8092163	EU734903	EU839769	EU815286	EU860154
**RAPHIDIOPTERA**						
**Raphidiidae**	*Mongoloraphidia martynovae* Aspöck & Aspöck	CASENT8092164	EU734870	EU839738	EU815252	EU860123
**NEUROPTERA**						
**Ascalaphidae**	*Libelloides longicornis* (Linnaeus)	CASENT8092187	EU734868	EU839736	EU815250	EU860121
**Berothidae**	*Lomamyia banksi* Carpenter	CASENT8092200	EU734869	EU839737	EU815251	EU860122
**Chrysopidae**	*Nothochrysa californica* Banks	CASC205	DQ399283	DQ414505	EU815265	EU860135
**Coniopterygidae**	*Cryptoscenea* nr. *obscurior* Meinander	CASENT8092175	EU734860	EU839730	EU815241	EU860112
**Dilaridae**	*Nallachius pulchellus* (Banks)	CASENT8092216	EU734875	EU839743	EU815257	EU860128
**Hemerobiidae**	*Notiobiella viridis* Tillyard	CASENT8092205	EU734883	EU839750	EU815266	EU860136
**Ithonidae**	*Ithone fulva* Tillyard	CASENT8092184	EU734865	EU839734	EU815247	EU860118
**Mantispidae**	*Ditaxis biseriata* (Westwood)	CASENT8092194	EU734862	EU839732	EU815243	EU860114
**Myrmeleontidae**	*Stilbopteryx costalis* Newman	CASENT8092178	EU734908	EU839773	EU815291	EU860159
**Nemopteridae**	*Chasmoptera hutti* (Westwood)	CASENT8092192	EU734851	EU839723	EU815232	EU860106
**Nevrorthidae**	*Austroneurorthus brunneipennis* (Esben-Petersen)	CASENT8092189	EU734848	EU839720	EU815229	EU860103
**Nymphidae**	*Nymphes myrmeleonoides* Leach	CASENT8092181	EU734884	EU839751	EU815268	EU860137
**Osmylidae**	*Kempynus kimminsi* New	CASC200	EU734867	DQ515501	EU815249	EU860120
**Psychopsidae**	*Psychopsis margarita* Tillyard	CASENT8092209	EU734897	EU839764	EU815280	EU860149
**Sisyridae**	*Sisyra vicaria* (Walker)	CASENT8092169	EU734904	EU839770	EU815287	EU860155
