# Supplementary material for: A Remarkable New Family of Jurassic Insects (Neuroptera) with Primitive Wing Venation and Its Phylogenetic Position in Neuropterida
Source: PLoS One. 2012 Sep 18;7(9):e44762. doi: 10.1371/journal.pone.0044762 (PMC3445537; doi:10.1371/journal.pone.0044762)
Supplement: Table S3 — Morphological scoring for selected Neuropterida families. (PDF) [file pone.0044762.s003.pdf]

A Remarkable New Family of Jurassic Insects (Neuroptera) with Primitive Wing Venation and its Phylogenetic Position in Neuropterida

Qiang Yang, Vladimir N. Makarkin, Shaun Winterton, Alexander V. Khramov, and Dong Ren

Table S3. **Morphological scoring for selected Neuropterida families**. Character states: '?', unknown; '-', inapplicable; '0?', '1?', poorly visible (mostly in fossil taxa), but most probable.

		1	2	3	4	5	6	7	8	9	10	11	12	13	14	15	16	17	18	19	20	21	22	23	24	25	26	27	28	29	30	31	32	33	34	35	36	37	38	39	40	41	42	43	44
1	Aetheogrammatidae	?	1	0	?	?	0	?	?	?	?	?	1?	1	0	3	0	0	3	2	0	1	2	1	0	0	3	0	0	0	0	0	1	0	0	0	?	?	?	?	0	0?	1	2	0
2	Archeosmylidae	?	?	?	?	?	?	?	?	?	?	0	0	?	1	1	0	0	0	0	0	1	0	0	1	0	1	2	0	0	0	0	0	0	0	1	2	2	2	?	0	0	0	?	0
3	Ascalaphidae	1	0	0	0	1	0	0	0	1	1	1	1	1	1	3	1	0	3	0	0	1	0	2	2	0	2	0	1	0	-	-	1	0	1	-	2	0	0	1	0	0	1	2	0
4	Ascalochrysidae	?	?	?	?	?	?	?	?	?	?	0	1	1	1	1	?	0	3	1	0	1	1	2?	1	0	2	0	?	1	?	?	?	0	0	?	?	?	?	?	0	0	1	0	1
5	Babinskaiidae	?	?	?	?	?	0	0	?	?	?	0	1	1	1	1	1	0	3	0	0	1	0	2	3	0	0	0	0	0	-	-	1	0	1?	-	2?	0	0	1?	0	0	1?	2	0
6	Berothidae	1	0	0	0	1	0	0	0	1	0	0	0	1	0	1	0	0	0	1	0	0	0	0	1	0	0	3	0	0	0	0	0	0	0	1	1	1	2	0	0	0	0	1	1
	Brongniartiellidae	?	?	?	?	?	?	?	?	?	?	0	0	1?	1	3	0	1	1	2	0	0	0	1?	0	0	1	1	0	0	0	1	1	0	0	2	1	1	?	?	0	?	?	1	0
8	Chrysopidae	1	0	0	2	1	0	0	0	1	1	0	1	1	0	2	0	0	0	0	0	1	0	1	1	0	1	3	0	0	1	0	0	0	0	1	1	0	0	0	0	0	1	1	0
9	Coniopterygidae	1	0	1	0	1	0	0	0	1	0	0	1	1	0	0	0	0	4	2	0	1	0	0	2	0	0	0	0	0	0	1	0	0	0	3	0	0	1	0	0	0	1	1	0
10	Dilaridae	1	0	0	0	1	0	0	1	-	0	0	0	0	0	1	0	0	0	1	0	0	1	0	1	0	1	0	0	0	0	0	0	0	0	1	2	2	2	0	0	0	0	2	0
11	Grammolingiidae	?	?	?	?	?	0	0	?	?	?	0?	0	1?	0	3	0	0	3	2	0	1	2	1	0	0	3	0	0	0	0	0	1	0	0	1	1	2	2	?	0	0	1	2	0
12	Hemerobiidae	1	0	0	1	1	0	0	0	0	0	0	0	1	0	1	0	1	1	0	0	0	0	0	1	1	1	2	0	0	0	0	0	0	0	1	1	1	0	0	0	0	0	1	0
13	Ithonidae	1	0	0	0	1	0	0	0	0	0	0	0	0	0	2	0	1	0	1	0	0	0	0	1	0	1	0	0	0	0	1	0	0	0	1	1	1	2	0	0	1	0	1	0
14	Kalligrammatidae	?	1	0	?	?	0	0	0?	?	?	1	0	1	1	3	0	1	1	2	0	0	2	1	0	0	3	0	0	0	0	0	1	0	0	0	1	1	2	?	0	0	1	1	0
15	Mantispidae	1	0	0	0	1	1	1	0	1	1	0	0	1	0	2	0	0	0	1	0	0	0	0	1	0	1	3	0	1	0	0	0	0	0	1	2	1	1	0	0	0	0	1	0
16	Mesochrysopidae	?	?	?	0	1?	0	0	0	?	?	0	1	1	1	2	1	0	3	0	0	1	0	1	1	0	2	2	0	1	1	1	1	0	0	1	2	0	0	1	0	0	1	1	1
17	Myrmeleontidae	1	0	0	0	1	0	0	0	1	1	0	1	1	1	3	1	0	3	0	0	1	0	2	2	0	2	0	1	0	-	-	1	0	1	-	3	0	0	1	0	0	1	2	0
18	Nemopteridae	1	0	0	1	1	0	0	0	1	1	0	1	1	1	2	0	0	3	0	0	1	0	2	2	0	1	2	0	0	-	-	1	0	1	-	3	0	0	1	1	0	1	2	0
19	Nevrorthidae	1	0	0	0	1	0	0	0	1	0	0	0	0	0	1	0	0	0	1	0	0	0	0	0	0	1	3	0	0	0	0	0	0	0	2	0	2	2	0	0	0	0	0	0
20	Nymphidae	1	0	0	?	1	0	0	0	1	1	0	0	1	1	2	1	0	3	0	0	0	0	2	0	0	3	0	0	0	0	1	1	0	0	2	3	1	0	1	0	0	1	2	0
21	Osmylidae	1	0	0	2	0	0	0	0	0	0	0	0	0	1	2	0	0	0	1	0	1	0	0	1	0	1	2	0	0	0	0	0	0	0	1	2	2	2	0	0	0	0	2	0
22	Osmylopsychopidae	?	?	?	?	?	?	?	?	?	?	?	0	0	1	2	0	1	1	1	0	0	0	0	1	1	0	0	0	0	0	0	1?	0	0	1	1	1	1	?	0	?	?	?	?
23	Palaeoleontidae	?	0	0	?	?	0	0	0	?	?	0	1	1	1	3	1	0	3	1	0	1	0	2	0	0	2	0	0	0	-	-	1	0	1	-	3	0	0	1?	0	0	1	?1	0
24	Panfiloviidae	?	?	?	0	?	0	0	?	?	?	0	0	1	0	3	0	0	3	1	0	0	2	0	1	0	3	0	0	0	0	1	1	0	0	2	2	2	2	0?	0	0	0	2	0
25	Parakseneuridae	?	0	0	?	?	0	0	?	?	?	1	0	1	0	3	0	1	1	1	0	0	1	0	1	0	2	0	0	0	0	1	1	0	0	0	1	1	1	0?	0	1	0	1	0
26	Permithonidae	?	0	0	?	?	0	0	0	?	?	0	0	0	0	1	0	0	0	1	0	0	0	0	2	0	1	1	0	0	0	0	0	0	0	1	0	0	2	?	0	0	0	0	0
27	Prohemerobiidae	?	?	?	?	?	?	?	?	?	?	0	0	0	0	1	0	1	0	1	0	0	0	0	1	0	0	2	0	0	0	0	?	0	0	1	1	1	1	?	0	?	0	?	0
28	Psychopsidae	1	0	0	0	1	0	0	0	0	0	0	0	0	0	2	0	1	2	2	0	0	1	0	0	0	1	2	0	0	0	0	1	0	0	2	2	2	2	0	0	1	1	2	0
29	Saucrosmylidae	?	?	?	?	?	0?	0?	0	?	?	?	0	?	1	3	0	0	0	0	0	0	0	0	0	0	3	0	0	0	0	2	1	0	0	2	2	2	2	?	0	0	1	2	0
30	Sisyridae	1	0	1	1	1	0	0	0	1	0	0	0	1	1	0	0	0	0	2	0	1	0	0	0	0	0	3	0	0	0	0	0	0	0	1	0	0	2	0	0	0	0	?	0
31	Raphidiidae	0	0	0	1?	0	0	0	1	0	1	0	1	1	0	2	0	0	0	2	1	1	0	0	3	0	0	2	0	1	0	1	-	1	0	1	0	0	0	0	0	0	0	1	0
32	Corydalidae	0	0	0	0	0	0	0	0	0	0	0	1	0	1	3	0	0	0	1	0	1	0	0	2	0	0	2	0	0	0	0	0	0	0	1	0	2	0	0	0	0	0	1	0
33	Sialidae	0	0	0	0	1	0	0	0	0	0	1	1	1	1	1	0	0	0	1	0	1	0	0	2	0	0	3	0	0	0	0	-	1	0	0	0	0	1	0	0	0	1	1	0
